# Molecular Determinants of Ligand Selectivity in Diverse Ionotropic Glutamate Receptors

**DOI:** 10.1021/acs.biochem.6c00443

**Published:** 2026-09-26

**Authors:** Yuhong Wang, Célia Jacquot, Nafthaniél Juliano Werleman, Timothy Lynagh

**Affiliations:** Michael Sars Centre, 1658 University of Bergen, Bergen 5008, Norway

## Abstract

Ionotropic glutamate receptors (iGluRs) mediate rapid signals between neurons by binding extracellular neurotransmitters and conducting ionic current across the cell membrane. Animal iGluRs can be divided into clades of phylogenetically related receptors, only some of which are present in mammals. Despite their name, many receptors are selectively activated by ligands other than glutamate, and despite high-resolution structural data on most mammalian iGluRs, the molecular basis for ligand selectivity in most clades is not clear. We questioned whether closely related iGluRs within phylogenetic clades share similar ligand selectivity profiles by testing several receptors from bilaterians, cnidarians, a placozoan, and a ctenophore, and by probing the molecular basis of ligand selectivity in iGluRs from different clades with site-directed mutagenesis. We identified glutamate-, glycine-, and GABA-gated channels from both the AKDF clade and the Epsilon clade, and a selectively aspartate-gated channel from the Epsilon clade. We show that in some cases, even very closely related channels from within the same clade and from the same animal species have diverged functionally, leading to channels gated by different agonists. Although we find special roles in ligand selectivity for glycine, threonine, and leucine residues in the “αF-GST” and “β10/αI-LE” motifs (mammalian AMPA receptor nomenclature), our results in diverse iGluRs warn that the effects of mutations, and thus the roles of these residues, are not always easy to predict.

## Graphical abstract

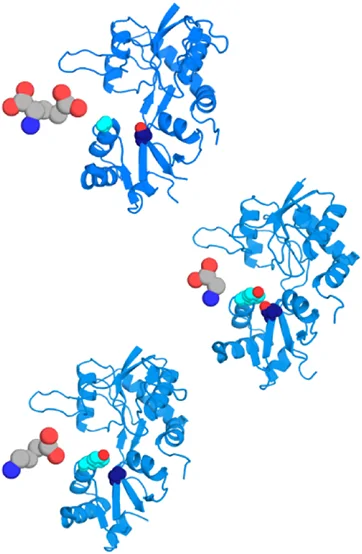

## Introduction

In most chemical synapses of the central nervous system, the membrane of the postsynaptic cell contains ionotropic glutamate receptors (iGluRs).^1–3^ iGluRs are tetrameric proteins in which four homologous subunits each contain an extracellular ligand-binding domain (LBD) and together form a central, membrane-spanning ion channel. The binding of neurotransmitter agonists, typically glutamate, to the LBDs rapidly opens the channel, leading to Na^+^, K^+^, and sometimes Ca^2+^ currents across the cell membrane, and thus to membrane depolarization, in neurons.^2^ iGluRs predate animals and occur in numerous organisms without neurons.^4,5^

Two classes of iGluRs predominate in the vertebrate brain, α-amino-3-hydroxy-5-methyl-4-isoxazolepropionate-sensitive iGluRs (AMPARs) and *N*-methyl-d-aspartate-sensitive iGluRs (NMDARs).^6,7^ Both are activated by synaptically released glutamate, but NMDARs also require a coagonist, glycine or d-serine, binding to two subunits in the tetramer.^2^ Another class of iGluR found in vertebrates and other complex bilaterians, Delta, also includes receptors for glycine, d-serine, and even γ-aminobutyric acid (GABA).^8,9^ Indeed, the iGluR epithet belies substantial diversity in LBD function, and outside of the classical vertebrate iGluR classes, the diversity is even greater. Several plant iGluRs are best activated by methionine.^5^ Insects have a number of odor-sensing ionotropic receptors (IRs) that evolved via a lineage-specific radiation of iGluR genes.^10^ Numerous early-diverging animal lineages, including Ctenophora (comb jellies) and Placozoa (thin, film-like animals), have iGluRs that are potently activated by glycine.^11–13^ Such glycine-gated receptors are found both in the “AKDF” clade of iGluRs, so named because it includes AMPA, kainate, Delta, and “Phi” iGluRs, and in the more distantly related “Epsilon” clade of iGluRs.^14^

Curiously, the clades of phylogenetically related genes that include glycine- and GABA-gated iGluRs also include glutamate-gated iGluRs.^9,11^ Even within the same animal, closely related iGluRs from the same phylogenetic clade may differ in their ligand preference.^11^ This suggests that iGluRs are evolutionarily pliable, in that iGluRs can easily undergo “ligand switching” to change their agonist preference. Given the role of these transmitters and receptors in fast signaling between neurons, ligand switching in iGluR genes could be linked to the evolution of the nervous system.^15^ However, without experimental data on diverse iGluRs, it is difficult to confidently assign agonist preference to iGluR genes and thus map the evolution of transmitter/receptor systems. Also, sequence-based or computational predictions of function are difficult when only a few mutations are required to produce a large functional change, as inferred from closely related receptors with different ligand preferences.

The first high-resolution structures of iGluRs offered a tentative explanation for ligand selectivity among iGluRs. Glycine-binding NMDAR LBDs (in the GluN1 subunit) and glutamate-binding AMPAR and NMDAR (in the GluN2 subunit) LBDs all show a similar, polar upper lobe, suitable for binding the α-carboxylate and α-amine groups that are conserved among amino acid ligands.^16,17^ But in the lower lobe, GluN1 subunit LBDs have a large tryptophan side chain, which may prevent access by larger agonists, whereas in glutamate-binding AMPAR and GluN2 subunits the equivalent residue is a smaller or more flexible leucine or methionine.^16–18^ However, experimental verification of the role of this residue in ligand selectivity is lacking, and recent work on a placozoan iGluR suggests that other, vicinal amino acid residues may drive ligand selectivity via polarity and volume in the LBD.^13^

Here, we sought to address two questions experimentally. First, we sought further evidence as to whether iGluRs within phylogenetic clades share similar ligand selectivity profiles or whether even closely related genes have diverged from each other functionally. Second, we questioned the molecular basis of ligand selectivity in several diverse iGluRs through experiments aimed at converting preference from one ligand to another by mutagenesis. We focused on the AKDF and Epsilon clades because both are known to include experimentally tractable homomers and to contain closely related receptors with different agonist preferences, providing an opportunity to investigate ligand switching during iGluR evolution. We find that many clades of iGluRs include receptors for different agonists, and that certain amino acid residues contribute to ligand selectivity in several, but not all, diverse receptors, presumably because of diversity elsewhere in the ligand-binding site—even among closely related receptors.

## Materials and Methods

### Molecular Phylogenetics

iGluR amino acid sequences were sought from species based on the diversity of metazoan lineages and the databases available at the time of the work, ultimately from three ctenophores, two poriferans, three placozoans, four cnidarians, and among bilaterians: eight deuterostomes, 12 protostomes, and five xenacoelomorphs (Table S1). Sequences were retrieved from typical public databases, including Ensembl, NCBI/GenBank, and KEGG, as well as more specific sources^19–29^ listed in Table S1. Depending on the database, a BLASTP or TBLASTN search^30^ was performed to find sequences. For each species, multiple searches were performed to ensure we retrieved diverse iGluR sequences: one using *Rattus norvegicus* GluA2 as a template, one using *Nematostella vectensis* 5500937 (epsilon); one using *R. norvegicus* GluN1; and one using *Oscarella carmela* comp20375_c0_seq1 (representing maximally diverse metazoan iGluRs according to previous work^14^). Two *Arabidopsis thaliana* (plant) GLR sequences, NP_187061.1 and NP_001030971.1 from NCBI/GenBank, were included as an outgroup for the phylogenetic tree. Amino acid sequences were aligned within Geneious Prime software using the MAFFT v7.490 ^31^ FFT-NS-i (maximum 2 cycles) algorithm, for a mix of speed and accuracy, and the BLOSUM62 scoring matrix^32^ to cope with the high diversity that occurs among iGluRs. Sequences from the same species that were >95% identical to others were removed to avoid excessive redundant sequences branching together. Aligned sequences were manually compared with well-characterized receptors such as *Rattus norvegicus* GluA2 to check for the presence of essential structural and functional domains, especially the LBD and the channel-forming third transmembrane segment M3 (Figure S1), and sequences without these were removed. (For example, rat GluA2 has an upper LBD PLTITLVR and an M3 SYTANLAAF motif; a sequence with upper LBD PLTITKER and M3 VYIGAYINH was kept; and a sequence with upper LBD RIQLDTYAR (insertion and low homology) and M3 ----IQGAW (numerous gaps) was discarded.). When several mostly identical sequences, e.g., transcripts of the same gene, were found, the most complete sequence, in terms of the presence of typical iGluR structural domains, was retained. We edited some apparently short sequences to make them more complete by finding similar overlapping transcripts or predictions; e.g., *Branchiostoma belcheri* XP_019645125.1 and XP_035694464.1 were nearly identical in a large overlapping region, but the former was more complete (homology with other iGluRs) in the C-terminal half and the latter was more complete in the N-terminal half (Table S2). Sequences that were more than 1500 or fewer than 350 amino acids long were removed to avoid confounding the alignment. All remaining sequences were realigned, and the final alignment contained 460 sequences with 5952 aligned positions (including gaps). After manual trimming of the alignment to remove highly variable regions and regions with gaps in numerous sequences, the alignment contained 694 amino acid positions. This trimmed alignment was used to generate a maximum likelihood tree with IQ-TREE v2.2 ^33^ with an LG + I + G4 substitution model^34^ selected by IQ-TREE using the Bayesian information criterion and with SH-aLRT^35^ and ultrafast bootstrap^36^ branch support. The full tree is shown in Figure S2. The untrimmed and trimmed alignments and the tree are freely available for download at https://zenodo.org/records/22765788.

### Molecular Biology

Genes were selected from throughout the AKDF and Epsilon branches of the iGluR tree for heterologous expression and functional characterization based on (1) sampling the diversity of genes throughout these branches and (2) the likelihood of heterologous expression as assessed through the presence of typical iGluR features and signal peptides (Figure S1,S2).^37^ For sequences that we selected based on phylogenetic position but were obviously incomplete or had no apparent signal peptide, we sought the same sequence or gene in another database and/or incorporated N-terminal sequence from another homologous receptor, as shown previously, e.g., *Branchiostoma lancelatum* iGluRs (Table S2).^14^ Generally, invertebrate coding sequences were optimized for expression in *Xenopus laevis* using the online iCodon tool^38^ (except for cnidarian sequences, which showed higher functional expression from nonoptimized cDNAs in preliminary experiments; Figure S3A), a Kozak consensus-like sequence was added,^39^ and sequences were silently mutated to remove any unwanted restriction sites, if present. Final cDNA sequences are shown in Table S2. These were commercially synthesized (GenScript Biotech and Synbio Technologies) and subcloned into a modified pSP64-poly­(A) vector containing *Xenopus laevis* β-globin 5′ and 3′ untranslated regions and a poly­(A) tail.^12^ The plasmid for *Rattus norvegicus* GluA2 (G/flip/Q isoform) in the pRK5 vector was a gift from Dr. David MacLean (University of Rochester Medical Center, NY, USA).

Site-directed mutagenesis was performed via PCR with partially overlapping mutagenic primers^40^ and Phusion Hot Start II DNA Polymerase (Thermo Fisher Scientific). All coding sequences were confirmed by Sanger sequencing (Genewiz). Plasmids were linearized at restriction sites downstream of the poly­(A) tail, when present, or downstream of the coding sequence when no poly­(A) tail was present, with *Hin*dIII (rat GluA2) or *Eco*RI (all others) (Thermo Fisher Scientific), and then purified using DNA Clean & Concentrator-5 columns (Zymo Research). Linearized DNA was used for RNA transcription with the SP6 or T7 mMESSAGE mMACHINE Transcription Kit (Thermo Fisher Scientific) for 2 h at 37 °C, followed by 15 min with DNase I at 37 °C to digest remaining template DNA. RNA was then purified with RNA Clean & Concentrator-5 (Zymo Research) or RNeasy kits (Qiagen).

### Heterologous Expression and Electrophysiology

Defolliculated *Xenopus laevis* stage V/VI oocytes were purchased from Ecocyte Bioscience (Germany) and shipped (for ∼24 h) in modified Barth’s solution (Ecocyte Bioscience). Oocytes were then stored at 18 °C in 50% (in water) Leibovitz’s L-15 medium (Gibco) containing an additional 0.25 mg/mL gentamicin, 1 mM l-glutamate, and 15 mM HEPES, pH 7.6, in glass Petri dishes or in 35 × 10-mm cell culture plastic dishes, sometimes with gentle shaking to prevent oocytes from flattening on the bottom of the dish. Oocytes were injected with 10 ng cRNA (iGluRs from *Rattus norvegicus* and *Branchiostoma belcheri*) or 20–40 ng cRNA (iGluRs from other animals) using a Nanoliter2010 microinjector (World Precision Instruments). Oocytes were then incubated in supplemented L-15 medium at 18 °C for 1–5 days, depending on levels of expression indicated by ligand-gated currents in preliminary experiments.

For each two-electrode voltage-clamp recording, a single oocyte was placed in an RC-3Z bath (Warner Instruments) and continuously perfused with ND96 bath solution containing 96 mM NaCl, 2 mM KCl, 1.8 mM CaCl_2_, 1 mM MgCl_2_, 5 mM HEPES, pH 7.5, adjusted with NaOH. The oocyte was impaled with borosilicate micropipettes filled with 3 M KCl and typically clamped at a holding potential of −80 mV (OC-725D amplifier, Warner Instruments; Axon Digidata 1550B digitizer, Molecular Devices). Currents were recorded with pClamp 11.1 (Molecular Devices), sampling at 1000 Hz and filtered at 200 Hz. Amino acid ligands (monosodium salts for glutamate and aspartate) were prepared as 1 M stocks in 96 mM NaCl, 2 mM KCl, and 5 mM HEPES at pH 7.5 and stored at −20 °C before being diluted into ND96 on the day of experiments. Bath solutions were exchanged with a VCS-8 pinch valve control perfusion system (Warner Instruments).

### Data Analysis

Peak current amplitudes were measured in Clampfit 11.1 (Molecular Devices) and entered into GraphPad Prism 10 (Dotmatics) for analysis. We treated peak current amplitude activated by various potential agonists at each receptor as a measure of that receptor’s ligand selectivity. This is not a direct measure of which agonist binds that receptor with the highest affinity or which agonist best opens the channel in that receptor, as rapid desensitization of some iGluRs masks maximal current amplitude in macroscopic recordings of thousands of receptors in relatively large oocytes where solution exchange is relatively slow. These receptor properties can only be dissected with lengthy electrophysiological studies at the microscopic level of relatively few receptors with fast solution exchange. We pursued our experiments with whole oocytes, however, where expression of many receptors enabled us to test more ligands on more receptors. We therefore analyzed relative agonist efficacy as follows. As shown in most figures, relative peak current amplitudes of different ligands (10 mM) were normalized to the peak current response of the most efficacious agonist (expressed as a fraction) at each oocyte/recording, and the mean ± SD are plotted; absolute current in μA (mean ± SD) is shown for the strongest agonist in figures and for all agonists in Table 1. For concentration–response experiments and for calculating EC_50_ values, concentration-dependent peak current amplitudes were normalized to the maximum peak current amplitude for that agonist and plotted against ligand concentration for each oocyte/recording using the 4-parameter nonlinear regression in GraphPad Prism 10; mean ± SD from several oocytes are reported in the main text; graphed concentration–response data are instead normalized to the maximum current amplitude of the preferred agonist at that receptor, and curves in graphs are fit to the already-averaged data points for visualization, not statistical analysis. We generally treated relative agonist efficacy (rather than potency) as the measure of selectivity, but this was generally consistent with potency for the ligands we considered, with just one exception, described in the relevant results section. When comparing ligand selectivity (relative agonist efficacies) of wild-type (WT) and mutant receptors, we did not perform statistical analyses: if WT receptors showed, e.g., no glycine-gated current and mutant receptors showed large glycine-gated currents, this was clear evidence of a change in relative agonist efficacy. When comparing agonist potency between WT and mutants, EC_50_ values were compared using one-way ANOVA with Tukey’s multiple comparisons test.

**Table 1:** Peak Current Amplitudes

	Peak current amplitude in response to indicated ligand (in μA, mean ± SD, n oocytes in brackets)^a^					
	Glutamate	Glycine	d-serine	Alanine	Aspartate	GABA
AKDF iGluRs						
*Rat* GluA2	0.85 ± 0.59 (5)	0.02 ± 0.02 (5)	0.04 ± 0.06 (5)	0 (4)	0 (3)	0 (5)
*Lingula* XP_013416771.1	12.4 ± 10.1 (5)	0.16 ± 0.19 (5)	1.00 ± 1.60 (5)	0.95 ± 1.70 (5)	0.27 ± 0.13 (5)	0.03 ± 0.02 (5)
*Hofstenia* 98026516	0.14 ± 0.08 (8)	0 (5)	0 (5)	0 (5)	0.08 ± 0.01 (5)	0 (5)
*Acanthaster* GluD	1.28 ± 0.48 (6)	0.25 ± 0.25 (6)	0.61 ± 0.55 (6)	1.34 ± 0.85 (6)	0.29 ± 0.23 (6)	9.31 ± 4.47 (6)
*Trichoplax* 19383^#^	0.81 ± 0.58 (5)	12.01 ± 5.10 (6)	10.36 ± 2.87 (5)	10.54 ± 4.40 (5)	0.39 ± 0.12 (5)	10.60 ± 4.38 (5)
*Nematostella* 5513056	0.18 ± 0.17 (8)	0.01 ± 0.00 (5)	0.01 ± 0.01 (5)	0.02 ± 0.01 (5)	0.09 ± 0.05 (7)	0.03 ± 0.05 (3)
*Nematostella* 5503753	0.01 ± 0.01 (5)	0.02 ± 0.01 (10)	0.01 ± 0.00 (7)	0.05 ± 0.02 (7)	0.01 ± 0.01 (5)	0.00 ± 0.00 (3)
*Exaiptasia* 110238381	0.10 ± 0.07 (7)	0.010 ± 0.00 (4)	0.01 ± 0.01 (4)	0.01 ± 0.01 (4)	0.03 ± 0.01 (4)	0.00 ± 0.00 (3)
Epsilon iGluRs						
*Branchiostoma* XP_019617116.1^sp^	0.49 ± 0.16 (6)	4.88 ± 2.35 (6)	2.35 ± 1.92 (6)	4.94 ± 2.42 (6)	1.26 ± 1.28 (6)	3.68 ± 2.59 (6)
*Branchiostoma* XP_019616632.1^db^	0.23 ± 0.09 (7)	4.68 ± 2.51 (6)	0.72 ± 0.38 (6)	0 (6)	5.98 ± 3.39 (7)	24.48 ± 7.32 (7)
*Branchiostoma* XP_019645125.1^sp^	6.21 ± 1.03 (7)	0.03 ± 0.03 (7)	0.06 ± 0.11 (7)	0.02 ± 0.03 (7)	0.02 ± 0.03 (7)	0 (7)
*Nematostella* 5511254^db^	0.06 ± 0.01 (16)	0.01 ± 0.01 (6)	0.01 ± 0.01 (8)	0.01 ± 0.00 (6)	0.06 ± 0.01 (8)	0.01 ± 0.01 (4)
*Nematostella* 5500937	0.10 ± 0.07 (9)	0.01 ± 0.01 (4)	0.02 ± 0.01 (4)	0.01 ± 0.00 (4)	0.07 ± 0.02 (4)	0.01 ± 0.01 (3)
*Trichoplax* 18262	0 (6)	1.56 ± 1.27 (6)	8.90 ± 2.59 (6)	0 (6)	0 (6)	0 (6)
*Euplokamis* AQX17743.1	0.60 ± 0.31 (5)	0.19 ± 0.07 (5)	0.25 ± 0.27 (5)	0.40 ± 0.09 (5)	17.3 ± 8.7 (5)	1.18 ± 0.97 (5)
Uninjected oocytes	0.01 ± 0.00 (6)	0.00 ± 0.00 (6)	0.01 ± 0.00 (6)	0.00 ± 0.00 (6)	0.01 ± 0.00 (6)	0.00 ± 0.00 (6)

a The apparent relative agonist efficacy of weak agonists relative to strong agonists here may differ from the relative agonist efficacy in the figures because this table includes strong agonist amplitudes from numerous batches of experiments, whereas weak agonist amplitudes were tested in parallel with and normalized to strong agonist amplitudes within the same batch of experiments, but in fewer total batches of experiments.

### iGluR Structural Models and Ligand-Binding Domain Terminology

For visualizing iGluR ligand-binding domains (LBDs), we used a published X-ray crystal structure of the rat GluA2 AMPA receptor LBD with PDB 1FTJ;^41^ an AlphaFold 2 model of the *Acanthaster planci* GluD LBD from a previous publication;^9^ and, for other receptors analyzed in detail, we uploaded amino acid sequences to the AlphaFold 3 server,^42^ used the first of five predicted models to visualize the LBD, and we show local confidence scores^42,43^ for these models in Figure S4. We refer to substructures within the LBDs of all receptors in this study according to the names and numbers of helices and strands in rat GluA2 AMPARs,^41,44^ e.g., helix αD, strand β10, etc., although the number of helices and strands in LBDs of other receptors probably differs.

## Results

### Functional Characterization of Diverse Metazoan iGluRs

Before testing the molecular determinants of ligand discrimination in any particular iGluR, we first sought to organize metazoan iGluRs into evolutionarily related clades using phylogenetic analysis, as others have done before.^13–15,45^ Our gene tree yielded the previously reported iGluR clades: Lambda, exclusive to Porifera (sponges); NMDA, found only in Cnidaria (sea anemones, jellyfish, etc.) and Bilateria (e.g., vertebrates, insects, mollusks); Epsilon, found in each of the five major metazoan lineages; and AKDF, a branch named after AMPA, kainate, Delta, Phi, and other uncharacterized “AKDF” iGluRs contained therein (Figure 1A, Figure S2).^14,45^

**Figure 1:**
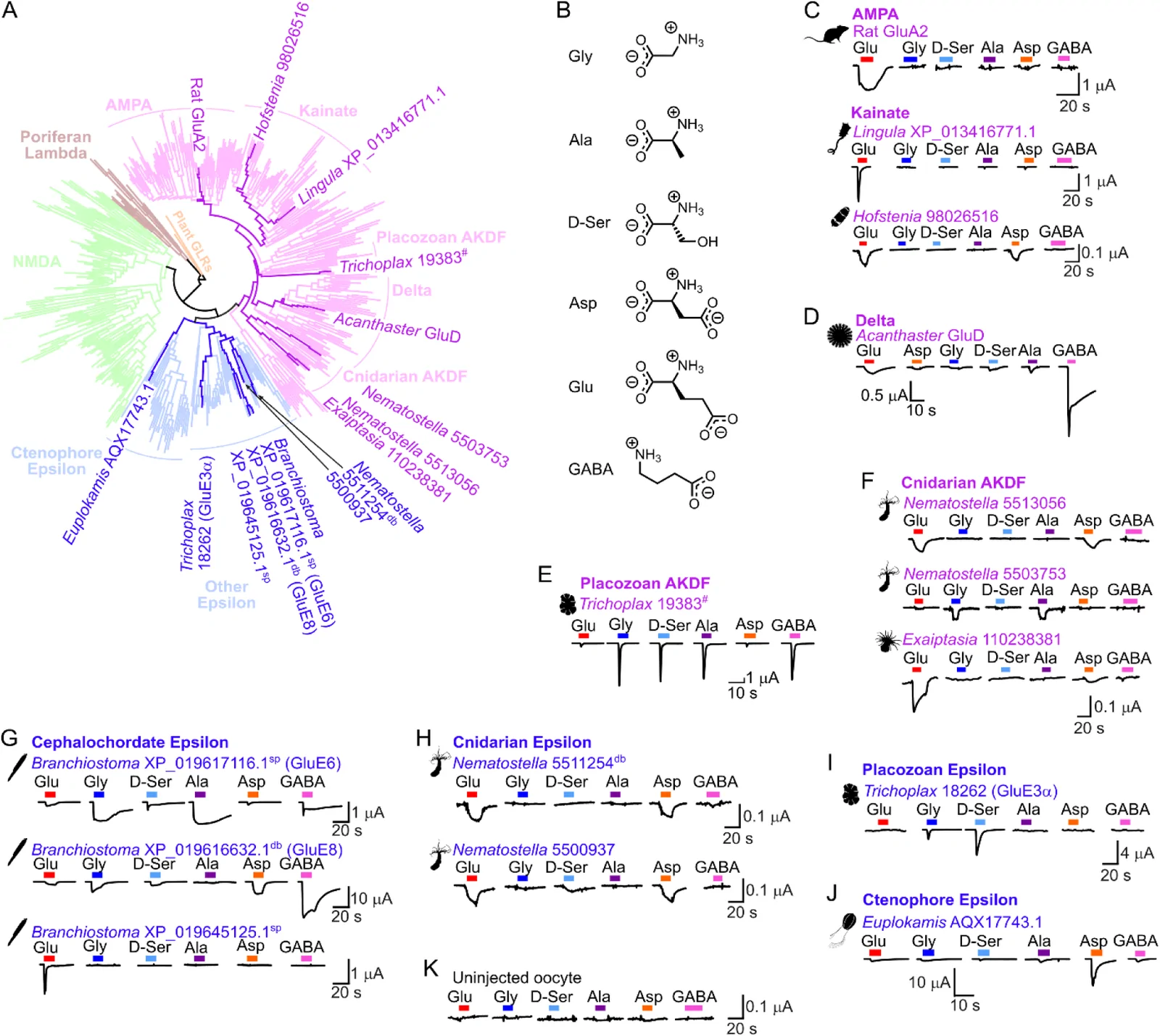
**Functional characterization of diverse iGluRs. A**, stylized animal iGluR gene tree (full tree including branch support in Figure S2; details in *Materials and Methods*). Bold branches/tips in the tree and corresponding gene names are the lineages/genes selected for study here. Selected branches are highlighted by arcs and named (“AMPA”, “Ctenophore Epsilon”, etc.) according to previous work.^14,45^ **B**, cartoon structures of potential amino acid ligands tested. L-amino acids unless indicated. Zwitterionic forms (at neutral pH) are shown. Most carbon and hydrogen atoms are not shown or labeled. **C–K**, example two-electrode voltage-clamp recordings of currents in oocytes injected with the indicated iGluR RNAs (names from selected previous studies shown in brackets^13,14^ ). ^sp, db^ Certain cDNA sequences were designed to include signal peptides from other receptors to enhance expression (sp) or were based on transcripts from databases other than the one we used to identify and name the receptor (db), detailed in Figure S1 and Table S2. ^#^ The *Trichoplax adhaerens* mutant 19383 receptor we used is activated by the same ligands that inhibit the constitutively active wild-type 19383 receptor (Figure S3D).

To establish if iGluRs within distinct gene clades show conservation of ligand recognition, we selected iGluR genes from throughout the AKDF and Epsilon branches with coding sequences in databases that seemed relatively complete (bold-colored branches and tips in Figure 1A), attempted to express them heterologously in *Xenopus laevis* oocytes, and measured currents using two-electrode voltage clamp in response to six amino acids as potential agonists (Figure 1B). This enabled a qualitative assessment of ligand selectivity.

Within the AKDF clade, we expressed kainate receptor genes from the spiralian bilaterian *Lingula anatina* and the xenacoelomorph bilaterian *Hofstenia miamia*; a Delta receptor from the echinoderm bilaterian *Acanthaster planci*; an AKDF gene from the placozoan *Trichoplax adhaerens*; and three AKDF genes from the cnidarians *Nematostella vectensis* and *Exaiptasia diaphana* (Figure 1A,C–F; Figure S3). As with closely related AMPA receptors, both kainate receptors we tested were best activated by glutamate (Figure 1C), with smaller currents activated by aspartate (relative aspartate-gated current amplitude of 4 ± 1% and 76 ± 10% for *Lingula* and *Hofstenia* kainate receptors, respectively, n = 5 or 8, Figure S3B). Absolute maximum currents, in μA, are shown in Table 1.

The *Acanthaster* Delta receptor was most strongly activated by GABA, as recently reported in a study of the Delta branch,^9^ followed by glutamate and then alanine (Figure 1D, Figure S3C). *Trichoplax* TRIADDRAFT_19383^#^ (^#^ denoting a mutant that facilitates ligand-gated currents, Figure S3D) was most sensitive to glycine, d-serine, and alanine, as reported recently,^12^ and also showed large GABA-gated currents (Figure 1E, Figure S3D). Oocytes injected with *Nematostella and Exaiptasia* AKDF RNAs showed relatively small currents in response to the tested ligands (Table 1), perhaps indicating low surface expression, but we observed glutamate-gated currents with *Nematostella* 5513056 and *Exaiptasia* 110238381, and glycine- and alanine-gated currents with *Nematostella* 5503753 (Figure 1F, Figure S3E). Again, the glutamate-gated channels were also activated by aspartate (relative current amplitudes of 94 ± 16% (n = 7) in *Nematostella* 5513056 and 21 ± 6% (n = 4) in *Exaiptasia* 110238381).

Across the Epsilon branch, we expressed three genes from the cephalochordate *Branchiostoma belcheri*, two genes from the cnidarian *Nematostella vectensis*, one gene from the placozoan *Trichoplax adhaerens*, and one gene from the ctenophore *Euplokamis dunlapae* (Figure 1A,G-J; Figure S3). The three *Branchiostoma belcheri* genes are paralogues specific to cephalochordates that diverged recently and are closely related (see the three *Branchiostoma* genes at the bottom of Figure 1A). Yet, remarkably, we observed different ligand selectivity profiles for all three receptors. *Branchiostoma* XP_019617116.1^sp^ and XP_019616632.1^db^, or *Branchiostoma* “GluE6” and “GluE8” according to a previous study,^14^ share 73% amino acid sequence identity (^sp^ and^db^ denoting modifications for successful expression; Figure S1 and Table S2). But whereas XP_019617116.1^sp^ was best activated by alanine and glycine, with smaller currents activated by GABA (67 ± 20%), d-serine (40 ± 20%), aspartate (22 ± 17%), and glutamate (17 ± 11%; all n = 6), XP_019616632.1^db^ was best activated by GABA, with alanine activating no current (0%, n = 6), and the other ligands activating relative currents of 3 ± 1% (glutamate, n = 7) to 30 ± 4% (aspartate, n = 7; Figure 1G; Figure S3F). In even greater contrast, XP_019645125.1^sp^ (not apparent in a previous *Branchiostoma* study^14^), which shares 40–43% identity with the other two receptors, was selectively activated by glutamate, with other compounds activating no current at all (n = 7; Figure 1G; Figure S3F). A previous study showed that *Branchiostoma lanceolatum* GluE1, with 33–37% sequence identity to those we tested, was selective for glycine over alanine, d-serine, and glutamate.^14^

The two cnidarian Epsilon receptors we tested, *Nematostella* 5511254^db^ and 5500937, both showed small responses to glutamate (0.06 ± 0.01 μA, n = 16; and 0.09 ± 0.07 μA, n = 9, respectively) and aspartate (0.06 ± 0.01 μA, n = 4; and 0.07 ± 0.02 μA, n = 6, respectively), and 5500937 also responded to d-serine (current amplitude of 21 ± 12% relative to glutamate (n = 7); Figure 1H, Figure S3G). Placozoan Epsilon receptor, *Trichoplax* TRIADDRAFT_18262, or GluE3α, according to recent nomenclature,^13^ was activated most efficaciously by d-serine, with smaller currents activated by glycine (17 ± 10%, n = 6; Figure 1I, Figure S3H). This loosely reflects the profile of the recently described *Trichoplax* Epsilon GluE1αA receptor^13^ (TRIADDRAFT_3218 in the database we used^27^), despite only 31% amino acid sequence identity between the two *Trichoplax* receptors. Finally, we also tested the activity of a receptor from the ctenophore-specific branch of the Epsilon family, *Euplokamis* AQX17743.1. In contrast to all other receptors we tested, this *Euplokamis* Epsilon receptor was selectively activated by aspartate, with relative current amplitudes for other agonists of just 4 ± 1% (glutamate), 1.3 ± 0.7% (glycine), and 3 ± 1% (alanine; all n = 5; Figure 1J, Figure S3I).

### Potential Molecular Determinants of Ligand Selectivity

These results substantially broaden our knowledge of the activity of iGluRs with diverse sequences, potentially helping us link sequence to function and address the question of the molecular determinants of ligand selectivity. We therefore aligned amino acid sequences of putative LBD segments, using knowledge from our results and those of others’ results. We restricted our alignment to a representative set of functionally characterized AKDF and Epsilon receptors, selected to broadly sample these clades rather than provide an exhaustive alignment of all available iGluR sequences, as well as three well-known NMDA receptor subunits for reference.

By homology with high-resolution structures of well-known iGluRs, such as the rat GluA2 AMPAR, amino acid residues from the upper lobe of the LBD that could interact with agonists include the side chain of Y471 (GluA2 numbering), the main chain of P499, the polar side chain of T501, and the basic side chain of R506 (Figure 2A,B). Considering our alignment of novel receptors, there are glutamate-, glycine-, and GABA-selective receptors with a Y or F at the Y471 position; glutamate-, glycine-, and d-serine-selective receptors with P, S, or nonpolar side chains at the P499 position; and there are glutamate-selective and glycine- or GABA-selective channels containing polar or nonpolar side chains at the T501 position (Figure 2A). The large, basic R506 residue is absolutely conserved among receptors discussed here (Figure 2A). Thus, there is no obvious link between amino acid identity in the upper lobe and ligand preference, suggesting that upper lobe residues are unlikely to contribute to ligand selectivity, as suggested previously.^14,17^ In the “hinge region” at the rear of the ligand-binding site is the Y753 side chain (Figure 2B), recently implicated in ligand selectivity in a placozoan Epsilon iGluR.^13^ However, there are glutamate-selective receptors with a Y or V in this position and glycine-selective receptors with a Y or F in this position (Figure 2A), suggesting this residue is not a principal determinant of ligand selectivity.

**Figure 2:**
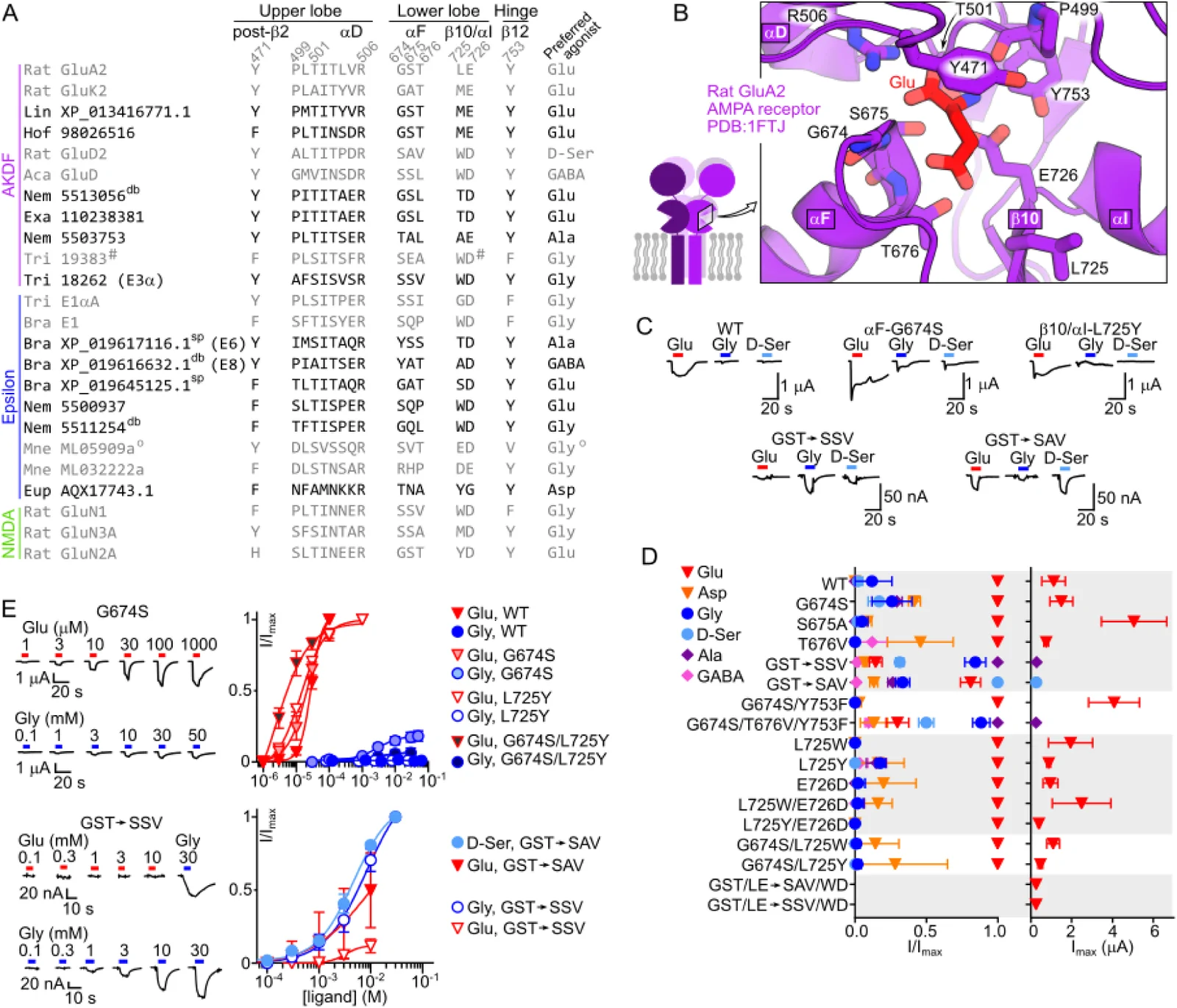
**Potential determinants of ligand selectivity in diverse iGluRs. A**, amino acid sequence alignment of segments of putative ligand-binding domains (LBDs) from selected iGluRs (names in brackets refer to nomenclature used elsewhere^13,14^ ). Structural elements and numbering (top) are from rat GluA2.^41 sp, db^ Certain cDNA sequences were designed to include signal peptides from other receptors to enhance expression (sp) or were based on transcripts from databases other than the one we used to identify and name the receptor (db), detailed in Figure S1 and Table S2. ^#^ The *Trichoplax adhaerens* wild-type (WT) 19383 receptor has a β/10-αI WY motif and is inhibited by glycine, whereas the mutant 19383 receptor we used has a β/10-αI WD motif and is activated by glycine.^12^ ^o^ *Mnemiopsis leidyi* 05909a is activated 25-fold more potently by glycine but more efficaciously by glutamate.^11^ **B**, magnified view of the glutamate (red) binding site in the X-ray crystal structure of the excised rat GluA2 AMPA receptor LBD (purple).^41^ **C**, ligand (10 mM) -gated currents in oocytes expressing the indicated WT and mutant rat GluA2 AMPA receptors. **D**, *left*, ligand (10 mM) -gated current amplitude normalized to that of glutamate at WT and mutant rat GluA2 AMPA receptors. *Right*, absolute maximum glutamate-gated current amplitude. **E**, example currents and mean (±SD, n = 4–5) normalized currents in response to increasing ligand concentrations.

Lower lobe residues that can interact with ligands include the GST motif at the top of α-helix F (“αF-GST”) and the LE motif between β-strand 10 and α-helix I (“β10/αI-LE”, Figure 2B). At the first position in αF-GST, all glutamate-selective receptors in our alignment have a small glycine residue (G674 in rat GluA2; Figure 2A). In contrast, none of the glycine- and other ligand-selective receptors have G at this position, with S and, more rarely, Y or A residues instead (Figure 2A). The β10/αI-LE motif is less obviously correlated with ligand selectivity, as e.g., glutamate-selective receptors have LE, ME, YD, ED, and TD; an alanine-selective receptor has TD; and glycine-selective receptors have WD, MD, WY, and DE (Figure 2A).

As the αF-GST sequence shows some correlation with ligand selectivity, we tested whether mutating the glutamate-selective rat GluA2 AMPA receptor to reflect glycine-selective receptors in this motif could affect its activity. WT receptors showed large responses to glutamate and no responses to glycine or d-serine, whereas receptors containing the single αF-G674S substitution showed large responses to glutamate and noticeable responses to glycine and d-serine (relative glycine- and d-serine-gated current amplitudes were 33 ± 14% and 24 ± 5%, respectively (n = 5; Figure 2C,D)). The β10/αI-L725Y substitution, but not the β10/αI-L725W substitution, also caused an increase in relative glycine-gated current amplitude, although to a lesser extent than αF-G674S (Figure 2C,D). We reasoned that a combination of αF-G674S and β10/αI-L725Y substitutions might further alter agonist selectivity, but glycine-gated currents in double-mutant αF-G674S/L725Y receptors were no greater and no more potent than in single-mutant αF-G674S receptors (Figure 2D,E). We also combined a hinge-region Y753F mutation with the αF-G674S mutation, as the converse F**→**Y mutation at this position in a *Trichoplax adhaerens* GluE3αA iGluR further increased the glutamate activity induced by other mutations,^13^ but this double-mutant AMPA receptor showed similar glutamate selectivity to WT (Figure 2D).

Replacing the glutamate-selective αF-GST motif with typical glycine-selective motifs via double- or triple-mutant αF-GST**→**SSV and αF-GST**→**SAV constructs drastically reduced current amplitude, although small ligand-gated currents were still measurable (Figure 2C,D). Remarkably, alanine activated the largest currents in αF-GST**→**SSV (*I*_max_ = 55 ± 36 nA, n = 8), with glycine and glutamate activating 84 ± 7% and 15 ± 4% relative current, respectively (both n = 4). d-serine activated the largest currents in αF-GST**→**SAV (*I*_max_ = 39 ± 18 nA), with glycine and glutamate activating 33 ± 5% and 81 ± 7% relative current, respectively (both n = 4). Unfortunately, when we replaced both αF and β10/αI segments with equivalents from glycine-selective receptors, oocytes injected with these quadruple- or quintuple-mutant receptor mRNAs showed no responses to any ligands (Figure 2D). The addition of the hinge-region Y753F mutation did not further alter the modest glycine and alanine selectivity of the αF-GST**→**SSV mutant (Figure 2D,E).

To test whether altered ligand selectivity in certain mutants was due to decreased glutamate and increased glycine potency, we performed concentration–response experiments with selected mutants. To our surprise, glutamate potency was not significantly different from WT in αF-G674S receptors (EC_50_ values: WT, 27.3 ± 1.8 μM, n = 6; G674S, 19.8 ± 3.2 μM, n = 5; *p* = 0.50) and was actually increased in αF-G674S/L725Y receptors (EC_50_ 6.4 ± 1.7 μM, n = 3; *p* = 0.004; Figure 2E). In contrast, αF-GST**→**SSV and αF-GST**→**SAV receptors both showed drastically reduced glutamate potency (Figure 2E; it was difficult to calculate EC_50_ as we did not see saturation even with 10 mM glutamate) together with increased glycine and d-serine potency (glycine EC_50_ for GST**→**SSV, 8.6 ± 2.5 mM; d-serine EC_50_ for GST**→**SAV, 4.7 ± 0.6 mM; both n = 4). Together, these results indicate that in rat AMPA receptors, the αF-GST segment contributes more than the β10/αI-LE segment to receptor preference for glutamate, that αF-T676 is more important than αF-G674 for glutamate potency, and that αF-G674, more so than αF-T676, seems to prevent glycine activity without necessarily driving glutamate potency.

### Determinants of Ligand Selectivity in Diverse Receptors from the AKDF Clade

Seeking to establish if ligand selectivity in other AKDF iGluRs relies on the same amino acid residues as in AMPA receptors, we tested the effects of similar substitutions in receptors from different parts of the AKDF branch. The *Acanthaster planci* GluD receptor is best activated by GABA and has αF-SSL and β10/αI-WD motifs in the lower lobe (Figure 3A). Replacing the first residue in the αF-SSL segment with the G residue from glutamate-selective receptors via the αF-S712G mutation drastically reduced maximum current amplitude but, unlike the converse substitution in AMPARs, it had little, if any, effect on the ligand selectivity profile (Figure 3A′,A″). The αF-L714T mutation in this motif also caused no increase in glutamate activity, with GABA selectivity ostensibly even greater than WT in this mutant (Figure 3A′,A″). In contrast to AMPARs, these mutations in the αF segment again tended to reduce potency of the preferred ligand (GABA EC_50_ values: WT 29 ± 11 μM, n = 4; αF-S712G 240 ± 48 μM, n = 4, *p* = 0.083; αF-L714T 670 ± 130 μM, n = 5, *p* < 0.001). Mutations in the β10/αI segment also reduced maximum current amplitude and GABA potency without altering the ligand selectivity profile (Figure 3A″,A‴; β10/αI-W762L GABA EC_50_ 1.1 ± 0.01 mM, n = 4, *p* < 0.001). We were unable to measure the combined effects of these mutations in the *Acanthaster* GluD iGluR, as we observed no ligand-gated currents in oocytes injected with double-mutant and quadruple-mutant receptor RNA (Figure 3A″).

**Figure 3:**
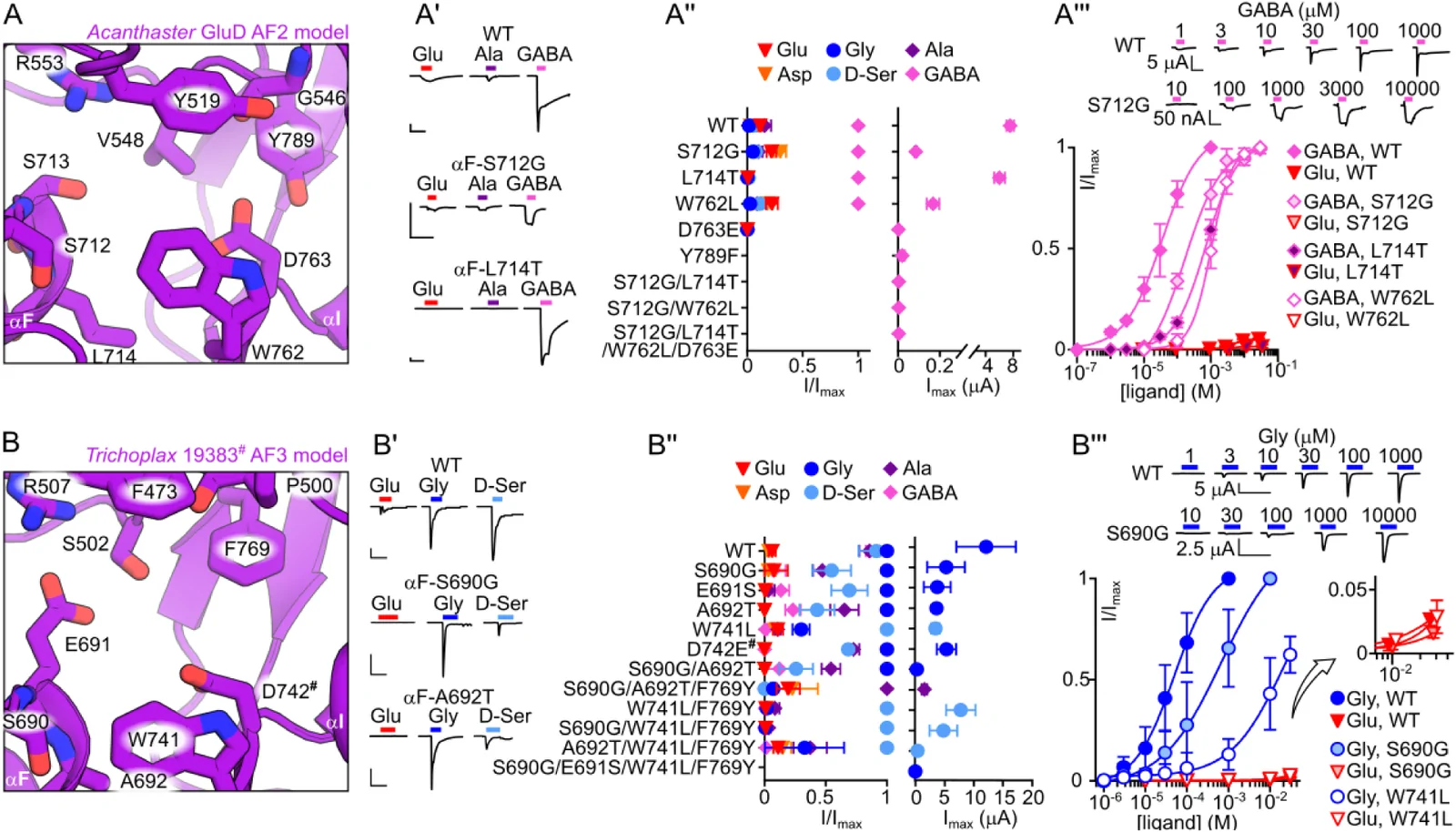
**Determinants of ligand selectivity in diverse AKDF iGluRs. A**,**B**, AlphaFold models of *Acanthaster planci* GluD receptor and *Trichoplax adhaerens* 19383 ligand-gated mutant^#^ receptor (Figure S3D) showing selected side chains and main chains in the ligand-binding domain. **A’**,**B’**, ligand (10 mM)-gated currents in oocytes expressing the indicated WT or mutant receptors. Scale bars: **A’**, x, 10 s; y, 0.25 μA; **B’**, x, 10 s; y, 1 μA. **A″,B″**, ligand (10 mM) -gated current amplitude normalized to that of the most efficacious ligand at WT and mutant receptors (left graph), and absolute, maximum ligand-gated current amplitude (right graph). For receptors where maximum current amplitude was close to zero, no relative current amplitudes could be measured. **A‴**,**B‴**, Mean (±SD, n = 3–6) normalized currents in response to increasing ligand concentrations (normalized to *I*_max_ for each construct; although glycine was more potent than d-serine at *Trichoplax* 19383^#^, d-serine was often more efficacious,^12^ especially at the W741L mutant).

The *Trichoplax adhaerens* 19383^#^ receptor, in which glycine, d-serine, and alanine activate large currents, has lower-lobe αF-SEA and β10/αI-WD residues (Figure 3B). Replacing the first residue of the αF-SEA segment with the equivalent residue from most glutamate-sensitive AKDF receptors via the αF-S690G mutation caused no increase in glutamate activity and thus no major change to the ligand selectivity profile, although there may have been a small preference for glycine over d-serine and alanine (Figure 3B′,B″). In fact, none of the lower-lobe substitutions that we made, including the αF-S690G/A692T double mutant, caused a large increase in relative glutamate activity in the AKDF-19383^#^ receptor, although αF-E691S and β10/αI-W741L substitutions decreased alanine and/or glycine activity (Figure 3B″). However, in the triple mutant that contained αF-S690G/A692T and hinge-F769Y substitutions, although currents were generally small and alanine remained a much stronger agonist than glutamate, glutamate activated larger currents than glycine and d-serine, indicating a substantial change in the ligand selectivity profile (Figure 3B″). In contrast to rat GluA2 AMPA receptors and similar to *Acanthaster* GluD receptors, we found that lower-lobe mutations reduced cognate agonist potency in *Trichoplax* 19383^#^ receptors without drastically altering the ligand selectivity profile (Figure 3B‴).

### Determinants of Ligand Selectivity in Receptors from the Epsilon Clade

We next questioned whether LBD lower lobe residues contribute to ligand selectivity in selected receptors from the Epsilon branch of iGluRs. Focusing this time on large functional differences among closely related receptors, we tested the glycine/alanine-, GABA-, and glutamate-selective Epsilon receptors from the chordate *Branchiostoma belcheri*.

The *Branchiostoma* XP_019617116.1 receptor (“GluE6” in a previous phylogenetic study^14^), best activated by glycine and alanine but with GABA activating 64 ± 16% current (n = 10), has relatively unusual αF-YSS and β10/αI-TD motifs (Figure 4A), and is 76% identical to GABA-selective XP_019616632.1 and 44% identical to glutamate-selective XP_019645125.1, excluding the signal peptide and intracellular C terminus. In XP_019617116.1, the αF-Y699G substitution drastically altered the selectivity profile, reducing maximal current amplitude but causing relatively large glutamate- and GABA-gated currents and only small glycine- and alanine-gated currents (Figure 4A’,A″). Adding the αF-S701T substitution to αF-Y699G to reflect the T residue found in XP_019616632.1, XP_019645125.1, and many other glutamate-selective receptors did not lead to a predictable change in ligand selectivity, as the XP_019617116.1 αF-Y699G/S701T double mutant responded most strongly to d-serine, not GABA or glutamate (Figure 4A″). Most surprisingly, the αF-Y699S single mutant, designed to reflect numerous glycine-selective receptors, showed even greater glutamate selectivity than the αF-Y699G mutant (Figure 4A’,A″). When combined with the β10/αI-T748W substitution and/or the hinge-region Y766F substitution in Y699S/Y748W and Y699S/T748W/Y766F mutants, also designed to reflect various glycine-selective receptors, the glutamate activity was drastically reduced or abolished (Figure 4A″), offering the first evidence in our study that large side chains at the β10/αI position decrease glutamate activity.

**Figure 4:**
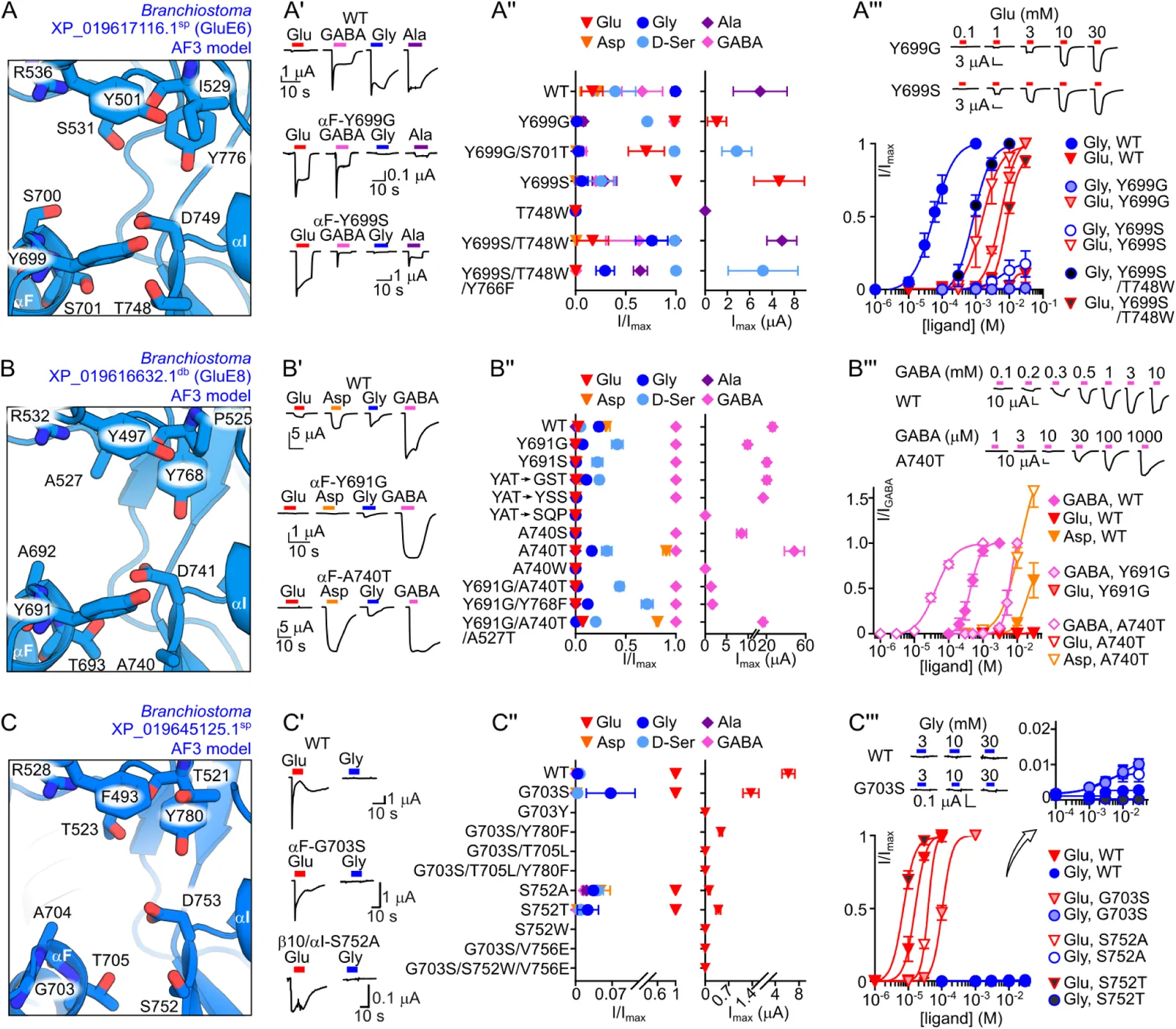
**Determinants of ligand selectivity in** *Branchiostoma* **Epsilon iGluRs. A-C**, AlphaFold3 models of the indicated *Branchiostoma belcheri* receptor ligand-binding domains showing selected side chains and main chains. **A’**,**B’**,**C’**, ligand (10 mM) -gated currents in oocytes expressing the indicated wild-type (WT) or mutant receptors. **A’’**,**B’’**,**C’’**, relative ligand (10 mM) -gated current amplitude normalized to that of the most efficacious ligand at WT and mutant receptors (left graph) and absolute maximum ligand-gated current amplitude (right graph). For receptors where the maximum current amplitude was close to zero, no relative current amplitudes could be measured. **A‴**,**B‴**,**C‴**, example currents and mean (±SD; n = 4–5) of normalized currents in response to increasing ligand concentrations. ^sp, db^ Certain cDNA sequences were designed to include signal peptides from other receptors to enhance expression (sp) or were based on transcripts from databases other than the one we used to identify and name the receptor (db), as detailed in Figure S1 and Table S2.

Concentration–response experiments showed that XP_019617116.1 αF-Y699G/S substitutions drastically reduced glycine potency and that the S substitution here caused greater glutamate potency than the G substitution (glutamate EC_50_ values: 6 ± 1.8 mM (n = 5) and 1.8 ± 0.9 mM (n = 5), respectively). This offers further tentative evidence that the glycine residue here, although linked to glutamate *activity*, is not necessarily better than serine for glutamate *potency*. Adding the β10/αI-T748W substitution to the αF-Y699S mutant increased glycine potency 5-fold and decreased glutamate potency 5-fold (glycine EC_50_ values: Y699S, 5.2 ± 1.1 mM (n = 3) and Y699S/T748W, 0.9 ± 0.2 mM (n = 4; *p* < 0.001); glutamate EC_50_ values: Y699S, 1.8 ± 0.9 mM (n = 5) and Y699S/T748W, 8.3 ± 0.5 mM (n = 4; *p* < 0.001)), indicating that a large residue here can fine-tune ligand potency.

The GABA-selective receptor *from Branchiostoma,* XP_019616632.1 (“GluE8” in a previous phylogenetic study^14^) shares the unusual Y residue and has αF-YAT and β10/αI-AD motifs (Figure 4B). Here, replacing the αF-YAT Y residue with the G found in most glutamate-selective receptors or the S found in most glycine- or d-serine-selective receptors did not decrease GABA selectivity and decreased the relative amplitude of currents gated by some other ligands, including glutamate (Figure 4B’,B″). Even the double αF-YAT**→**YSS and **→**GST mutations, reflecting closely related alanine/glycine-selective and distantly related glutamate-selective receptors, respectively, did not lead to a decrease in GABA selectivity (Figure 4B″). We tried to test what effect an SQP motif here would have on ligand selectivity, given the presence of this motif in the glycine-selective GluE1 receptor of *Branchiostoma lanceolatum* ^14^ and in the glutamate-selective *Nematostella* 5500937 receptor (Figure 2A), but we observed no ligand-gated currents at all in oocytes injected with XP_019616632.1 αF-YAT**→**SQP mutant RNA (Figure 4B″). Regarding the β10/αI-AD motif, we mutated the A residue to S and T to reflect glutamate-selective XP_019645125.1 and alanine/glycine-selective XP_019617116.1 receptors, respectively. Although A740S-expressing oocytes showed smaller and A740T-expressing oocytes showed larger GABA-gated currents than WT-expressing oocytes, GABA remained the most efficacious agonist at both mutants (Figure 4B″). However, the ligand selectivity profile at A740T receptors was noticeably altered, with aspartate—not glycine/alanine as anticipated—activating currents close in amplitude to those of GABA (Figure 4B’,B″). At a higher concentration of 30 mM, aspartate activated currents larger than GABA (Figure 4B″), although at this concentration of aspartate, inward currents could be larger due to additional Na^+^ in the extracellular solution because of the Na^+^/aspartate salt. Surprised that mutant XP_019616632.1 receptors that contain αF-GAT and -GST motifs and are so closely related to a glutamate-gated channel would not show any glutamate activity, we also considered the nonpolar A527 residue in the upper lobe of XP_019616632.1 (Figure 4B), which is a polar residue in most glutamate-selective receptors, with exceptions such as vertebrate kainate receptors (Figure 2A, T501 in rat GluA2). But even triple mutant A527T/Y691G/A740T receptors, essentially containing an αF-GAT motif and two other substitutions, remained strongly selective for GABA over glutamate, glycine, and d-serine (Figure 4B″).

Consistent with XP_019617116.1 results, the XP_019616632.1 αF–Y691G substitution decreased glycine- and alanine-gated current amplitude, but in contrast to XP_019617116.1 results, the substitution did not increase glutamate activity, and GABA remained the greatest agonist (Figure 4B’,B″). GABA potency was reduced by the substitution, however (GABA EC_50_ WT 437 ± 47 μM, n = 6; Y691G, 4.9 ± 1.4 mM, n = 5; *p* < 0.001). The β10/αI-A740T substitution increased GABA potency ∼ 10-fold, although not significantly different from WT by one-way ANOVA (GABA EC_50_ for A740T, 44 ± 6 μM, n = 4; *p* = 0.880), tentatively implicating side-chain position here in ligand potency again. Effects of the β10/αI-A740T substitution on glutamate and aspartate potency were difficult to measure due to inactivity and low potency, respectively (Figure 4B‴, could not fit curves or calculate EC_50_).

Finally, we investigated the functional role of equivalent residues in the glutamate-selective XP_019645125.1 receptor (this gene was not apparent in previous *Branchiostoma* work^14^). Like XP_019616632.1 and unlike XP_019617116.1, substitution of the primary αF motif residue had little effect on ligand activity, although the αF-G703S mutation did cause a very small increase in glycine activity (Figure 4C’-C‴). Oocytes injected with RNA encoding the αF-G703Y mutant, or any double mutants incorporating the αF-G703S mutation, showed no ligand-gated currents whatsoever (Figure 4C’). β10/αI-S752A and -S752T single-mutant receptors seemed to show slight increases in glycine-gated relative current amplitude, although glutamate was still the most efficacious ligand and maximum current amplitude was greatly reduced in these mutants, making the small increase in relative glycine activity questionable (Figure 4C’,4C″). Glycine potency was difficult to assess for each mutant tested, but we could measure glutamate potency (Figure 4C‴). This showed that the αF-G703S mutation decreased glutamate potency (glutamate EC_50_ WT 27 ± 13 μM, n = 6; G703S 104 ± 7 μM, n = 4; *p* < 0.001), in contrast to the increased glutamate potency with the homologous substitution in rat AMPARs (Figure 2E). The β10/αI-S752A and -S752T mutations caused an insignificant decrease (EC_50_ 35 ± 5 μM, n = 4, *p* = 0.479) and a significant increase (EC_50_ 8 ± 2 μM, n = 4, *p* = 0.016) in glutamate potency, respectively, again suggesting a correlation between the size of the primary β10/αI residue and glutamate potency, as in XP_019617116.1. We could not measure any activity with the less subtle β10/αI-S752W substitution.

## Discussion

### Context and Limitations

Previous studies have shown that iGluR genes are found throughout the five major lineages of animals, that iGluR genes can be divided into phylogenetically related clades that have been lost or conserved differently in different animal lineages, that some iGluRs are activated by amino acid ligands other than glutamate, and that mutations to ligand-binding residues, as evidenced by mammalian iGluR X-ray structures, can drastically alter agonist activity. We questioned whether clades of closely related iGluRs share similar ligand selectivity profiles and probed the molecular basis of ligand selectivity in iGluRs from different clades.

We identified glutamate-, glycine-, and GABA-gated channels from both the AKDF clade and the Epsilon clade, and a selectively aspartate-gated channel from the Epsilon clade. We show that in some cases, even very closely related channels within the same clade and from the same animal species have diverged functionally, leading to channels gated by different agonists. By revealing the function of WT receptors that diverge substantially from classical (mammalian) iGluRs in amino acid identity in the lower lobe of the LBD, with e.g., noncanonical αF-YAS and β10/αI-AD or -YG motifs, our results shed new light on the molecular basis of ligand recognition. While our mutagenesis results across diverse iGluRs confirm that certain residues play key roles in ligand selectivity, these results warn that the effects of mutations, and thus the roles of these residues, are not always easy to predict.

Our experiments with whole oocytes, which can express many receptors, enabled us to express numerous novel receptors and establish qualitative ligand selectivity profiles. We acknowledge that, especially for iGluRs that can desensitize quickly, quantitative measurement of ligand selectivity, i.e., agonist binding affinity and agonist efficacy, would require fast solution exchange and/or recordings from relatively few channels. Gratifyingly, WT AMPARs in our experiments showed the expected selectivity for glutamate over glycine, based on years of study of this receptor.

### Diverse Amino Acid Ligands Activate Animal iGluRs

Our results add to the list of glutamate- and glycine-gated iGluRs established for Bilateria, Placozoa, and the earliest-branching animal lineage, Ctenophora,^11–14^ by identifying both glycine- and glutamate-gated channels in Cnidaria. Cnidaria and Bilateria likely diverged from a common ancestor with neurons, and whereas cnidarian nervous systems are generally dispersed networks of neurons connected by bidirectional synapses, bilaterians evolved unidirectional synapses and centralized nervous systems.^46,47^ Our work, together with that of others, now establishes that both lineages have glycine- and glutamate-selective AKDF iGluRs for potential excitatory signaling. Concluding that such excitatory glycine and glutamate receptors were present in the neurons of the ancestral planulozoan is difficult, however, as iGluR ligand preference seems to “switch” numerous times within individual lineages, as reflected by differing ligand selectivity among closely related sea anemone AKDF iGluRs and among closely related cephalochordate Epsilon iGluRs (below). Although many cnidarian Epsilon iGluRs remain to be tested, the two we tested appear glutamate-selective. A recent study identified a chloride-permeable pentameric ligand-gated ion channel in *Nematostella vectensis*,^48^ and thus glutamate could mediate both excitatory and inhibitory signaling in cnidarians. In a single-cell RNA-sequencing study of *Nematostella*, AKDF iGluR 5513056 was expressed in neurons, whereas expression of the other genes we tested was below the limit of detection in all cell types.^49^

Our results also add to a growing list of non-glutamate/glycine amino acid-gated iGluRs. Delta iGluRs, a Bilateria-specific branch of the AKDF clade, include GABA-gated channels in numerous invertebrates and primarily glycine- and d-serine-sensitive iGluRs in vertebrates.^9,50^ Our study reveals a GABA-gated Epsilon iGluR, XP_019616632.1 (“GluE8” in a previous phylogenetic study^14^) from the cephalochordate *Branchiostoma belcheri*, suggesting that GABA could initiate excitatory postsynaptic potentials in bilaterian neurons via iGluRs from several different clades. GABA selectivity in this branch of the iGluR tree appears to have arisen relatively recently, as this small, cephalochordate-specific branch includes a relatively early-branching *Branchiostoma lanceolatum* glycine-selective receptor (GluE1), along with *Branchiostoma belcheri* glutamate-selective XP_019645125.1, GABA-selective XP_019616632.1, and glycine/alanine-selective XP_019617116.1 (Figure 1A).^14^

We identify AQX17743.1 as a selective aspartate receptor from *Euplokamis dunlapae*, an early-branching ctenophore lineage and thus from one of the very earliest branching animal lineages.^51^ Several iGluRs are sensitive to aspartate in addition to their preferred agonist, e.g., vertebrate NMDARs,^52,53^ but AQX17743.1 seems unique so far, with aspartate gating large currents with an EC_50_ of 12 μM (Figure S3I) and other ligands barely activating currents even at 10 mM. Although aspartate is present at micromolar and millimolar concentrations in brain tissue, it remains controversial whether it is loaded into presynaptic vesicles and released like other neurotransmitters in vertebrates.^54^ However, ctenophores probably rely on different proteins for loading excitatory vesicles, given their lack of vesicular glutamate transporter orthologues, raising the possibility they could utilize glutamate and aspartate differently from vertebrates.^15^ Aspartate has been experimentally detected in several ctenophore species, and, while these do not include *Euplokamis*,^55^ nervous system architecture appears similar in *Euplokamis* and other ctenophore lineages.^56^ AQX17743.1 is part of a distinct branch within the iGluR tree that also includes genes from other ctenophore species, but whether aspartate-gated channels are conserved in other species requires more experiments, as this is the only gene we tested.

We have treated all genes in our study as homotetramers, but we acknowledge that closely related genes within clades could foreseeably hetero-oligomerize. This is the case, for example, with the four vertebrate AMPAR genes, which are all expressed as homotetramers but readily form heterotetramers with the others upon coexpression.^2^ Some of the more closely related genes we have tested here, such as *Branchiostoma* Epsilon, also have several closely related paralogues and could foreseeably heteromerize, thus forming receptors for several ligands. However, it is worth noting that the four closely related vertebrate GluN2 paralogues do not heteromerize with each other and instead heteromerize only with the more distantly related GluN1. Thus, predicting which subunits heteromerize is not easy, and expressing numerous combinations to identify heteromers would be a lengthy study.

### Roles of Different Lower Lobe Residues in Ligand Selectivity in Diverse iGluRs

We aimed to identify molecular determinants of ligand selectivity in iGluRs both by measuring ligand selectivity in receptors of diverse sequence—a “natural” mutagenesis experiment—and by comparing the effects of our own site-directed mutations designed to mimic receptors for other ligands. The lower lobe residue that stands out as the most important determinant of ligand selectivity in our results is the first residue of the GST motif at the top of α-helix F “αF-GST” in glutamate-selective AMPAR and NMDAR subunits; “αF-SSV” or “αF-SAV” in glycine/d-serine-selective NMDAR subunits). This is consistent with previous hypotheses on the determinants of ligand selectivity based on analyses of characterized receptors and their amino acid sequences.^14,45^ It is also consistent with a recent, detailed experimental dissection of the placozoan glycine-selective GluE1αA iGluR, although in that study the cognate αF-SSI S**→**G substitution was only tested in combination with other mutations, e.g., αF-SSI I**→**T, which together led to large glutamate-gated currents.^13^

We show that in rat AMPARs, the G**→**S mutation induces substantial glycine activity in an otherwise glycine-insensitive receptor, although glutamate remains the more efficacious and potent agonist. More dramatically, in the cephalochordate XP_019617116.1 Epsilon iGluR with an αF-YSS motif, the Y**→**G mutation converted glycine/alanine selectivity into glutamate/GABA selectivity, practically abolishing glycine/alanine activity. However, the Y**→**S mutation in this receptor also led to glutamate selectivity, and similarly, the cnidarian WT 5500937 Epsilon iGluR with an αF-SQP motif responded to glutamate and not to glycine. Furthermore, although the AMPAR αF-GST G**→**S mutation induced glycine activity, it also slightly increased glutamate potency. Thus, a glycine residue may occur at this position in most glutamate-selective receptors, not because it is best for glutamate activity but because it is unsuitable for glycine activity. Further supporting this notion are our results that e.g., αF-SSV S**→**G mutations in glycine-selective receptors always decreased glycine potency and/or efficacy, and that the αF-GAT G**→**S mutation conferred small but noticeable glycine-gated currents in an otherwise glycine-insensitive Epsilon XP_019645125.1 receptor.

In the two other AKDF iGluRs and the one other Epsilon iGluR we tested, despite decreased potency of the original preferred ligand, αF-SSV S**→**G, αF-SEA S**→**G, αF-YAT Y**→**G, or Y**→**S mutations led to no increase in the activity of glutamate. While not inconsistent with the above notion of glycine’s reliance on, and glutamate’s “indifference” to, the first residue of the motif, this suggests that other residues must contribute substantially to ligand selectivity. This also suggests that the residues “used” by genes during evolution when “switching” their ligand preferences are not always the same—for e.g., G**→**Y, S**→**Y, or S**→**G in the case of *Branchiostoma* Epsilon iGluRs, but presumably other residues in receptors where mutations of the first residue have less effect.

Our results identify αF-GST and β10/αI-LE as two other important residues in ligand selectivity in diverse iGluRs, although our study was limited to relatively few positions. In most cases, strong effects on ligand selectivity via these positions manifested only when combined with αF-GST mutations, with the possible exception of *Branchiostoma* GABA-selective Epsilon iGluR XP_019616632.1, where the β10/αI-AD A**→**T mutation made aspartate a more efficacious agonist than GABA. In early structural studies of AMPARs and NMDARs it was suggested that moderately sized β10/αI-LE residues (like leucine or methionine) allow glutamate binding, whereas bulkier β10/αI-WD residues accommodate only the smaller glycine ligand.^17^ Indeed, the β10/αI-TD T**→**W mutation increased glycine activity in the glycine/alanine-selective Epsilon receptor XP_019617116.1. However, in the closely related but glutamate-selective Epsilon receptor XP_019645125.1, we observed a positive correlation between side-chain size and glutamate potency, as β10/αI-AD, -SD, and -TD motifs showed increasing glutamate potency. Thus, the contribution of this residue is either different between glycine-selective and glutamate-selective receptors, or it is other side-chain properties than just size that determine its effect on selectivity.

αF-GST or αF-SSV residues have long been implicated in glutamate and glycine recognition, respectively, with polar interactions between the glutamate ligand γ-carboxylate and the αF-GST hydroxyl side chain evident in AMPAR and NMDAR (GluN2) LBD X-ray structures.^16,18^ We now show that a single αF-GST T**→**V mutation has no effect on the preference for glutamate over glycine, but, when added to the αF-GST G**→**S mutation, converted the modest glycine activity of the latter to a preference for glycine. Our experiments suggest this is in large part due to decreased glutamate potency via the αF-GST T**→**V mutation, which is in strong contrast to the αF-GST G**→**S mutation in AMPARs that slightly increased glutamate potency. Curiously, the correlation between the αF-GST motif and glutamate potency does not extend to all iGluRs. In fact, even within AKDF iGluRs, αF-SSL and αF-SEA L**→**T and A**→**T mutations abolished the modest glutamate activity present in starfish Delta and placozoan AKDF receptors, and the WT cnidarian AKDF 110238381 appears strongly glutamate-selective despite a native αF-GSL motif. Thus, this residue may contribute strongly to ligand selectivity in some cases by selectively altering the potency of different ligands, but perhaps its mutation does not allow a previously inactive ligand to become an agonist.

### Outlook

By uncovering the function of diverse iGluRs, our study identifies novel ionotropic receptors in diverse animals. Although we have only tested a small number of the many iGluRs from each animal considered, this may help delineate signaling systems in different branches of the animal tree. Our results suggest caution when inferring the functional identity of ancestral complements of iGluRs, however, as “ligand switching” e.g., from a preference for glycine to a preference for glutamate or GABA, seems to occur among closely related receptors in different animal lineages independently. Our broad experimental survey, combined with mutagenesis on several diverse receptors, strongly implicates the αF-GST residue in driving ligand selectivity, with additional contributions coming from αF-GST and β10/αI-LE residues. At the αF-GST position, a glycine residue is incompatible with glycine (agonist) activity, whereas a serine residue seems compatible with glycine and glutamate agonist activity, but the potency of the two ligands and thus selectivity may be determined by identity at the αF-GST and β10/αI-LE positions. Thus, our broad study should enable future studies to dissect the mechanistic contributions of these residues to ligand affinity and channel gating, and, by offering sequence data on receptors for various ligands, help identify additional residues outside of the ligand-binding site that contribute to ligand selectivity. Such residues could potentially be found in the hinge region at the rear of the LBD,^57^ the front/outer edge of the lower lobe,^58,59^ or in less-conserved upper-lobe residues,^8^ as mutations in such sites in certain mammalian receptors can selectively alter the effect of different agonists.

## Supplementary Material



## Notes

Figure S1, Amino acid sequence alignment ; Figure S2, Animal iGluR gene tree; Figure S3, Additional experimental data; Figure S4, Structural model information; Table S1, Public databases used; Table S2, synthetic cDNA sequences (PDF)
