# Supplementary material for: Molecular Determinants of Ligand Selectivity in Diverse Ionotropic Glutamate Receptors
Source: Biochemistry. 2026 Sep 26;65(19):3116–29. doi: 10.1021/acs.biochem.6c00443 (PMC13648236; doi:10.1021/acs.biochem.6c00443)
Supplement: Supplementary file 1 [file bi6c00443_si_001.pdf]

SUPPORTING INFORMATION

Molecular determinants of ligand selectivity in diverse ionotropic glutamate receptors

Yuhong Wang[1], Célia Jacquot[1], Nafthaniél Juliano Werleman[1], Timothy Lynagh[1,*]

[1] Michael Sars Centre, University of Bergen, Bergen, 5008, Norway

* For correspondence, tim.lynagh@uib.no

Contents:

Page S2, S3 Figure S1. Amino acid sequence alignment of 15 iGluRs investigated here

Page S4-S6 Figure S2. Animal iGluR gene tree

Page S7 Figure S3. Heterologous expression and experimental characterization of diverse iGluRs

Page S8 Figure S4. New Alphafold3 models

Page S9 Table S1. Databases from which diverse iGluR sequences were retrieved

Page S10-S16 Table S2. Synthetic cDNA sequences used

Page S17-S18 Supporting Information references

```
                             Predicted signal peptide/sequence from another gene                     Extracellular N-terminal domain...
            Rat GluA2 MQ---------------KIMHISVLLS-PVLWGLIFGVSS--------NSIQIGGLF--PRGADQEYSAFRVGMVQFST-------SEFRLTPHIDNLEVANSFAVTNAFCSQFS------ 82
  Lin GluK-XP_013416771 MN-------------TVRIFGVLAGLC-LNLGTFILGQDT--------RRIKIGAVF--EGASSIEELAFKYAVNKVNSEISAT-LNGVTLEYELQKVTS-DSFQTSKKVCRLLKDG---- 91
   Hof GluK-98026516 MENRKSLLPKSAIKVRKKSLEPFGFPLAVLFCVLLLPNSVQSQTQ----QTLNIGAIFPDTSPSSLSQLAFQYAVQNANERSG----SNVKLRAFTKKIDITDSFQANKAACELLNAAEGTS 114
            Aca GluD ME-----------------IPQWIALTCLLLMYILEGVVTSERRVFP-SVIKVGVIL--EQPSSDEDEMIRLSFQHINQNGNIL--PSTRLDYTVARIASTDPFAAVKAACSMLN------ 93
       Nem 5513056 db MG---------------------IIQVCLYFTFIFWNGFSSGEDV---QQNIAGVVY--HKTDSVLFKAFKAGIKRLNEDKMLP--HNKTLEIAQFKTDGRNLLGC--DAVKKLR------ 85
       Exa 110238381 MA-----------------LGVCFIPV-VWFSILLTCVQSSLKSKGD-WQPIIGVLY--HPHDEPLYESLRQTINRINKQNTL---PVARLTTISFKTHSDKILGY--DAAKKLK------ 89
         Nem 5503753 MT------IRASWVI-RQTVMHQGPCVC-VMLMLLFHLPESASVP----SVVYIAQLG--MSPSQQPLNAVESAIRYVNKRSDLL--PSTTIKLLQETYSKDNLLQY---ALNLSS------ 97
          Tri 19383# MP-------------------ISKLVI-LLYILIFVSAEEIPIVKVNHNVIKIGVFM---EKSSIEGRTVRETIDFINKNKVYP-LQNATLTPVFQAIDKDSPYVVSRQVCQFIE------ 91
       Tri 18262 (E3α) MM---------------KPLNSSFLVI-YVFVTLLIRAPVIAVQANR-MEIPIAVLA--DGREQKFHDIIQLAADHINGDPAFA--FNIMIKLKIYNVTNYQPFLCQQEGCRAVD------ 94
 Bra XP_019617116 sp (E6) MQ---------------KIMHISVLLS-PVLWGLIFGVSS--------NSISRSALS--MSEFPAASSALKLAVEYVNENGLV---PNVILEYIENTTTALAFLDMIQHECYQAK------ 86
 Bra XP_019616632 db (E8) -------------------MAAGLVLL-LVASAASLATAE--------TTVRFGVLS--LSDFPAASSAVKLAVQYVNEKQLV---PNVTLKYIENSTASLTSLDLIKDECYQAS------ 82
    Bra XP_019645125 sp ME-----------------GTRATVLA-FLIVAMTFTTSMA-------AVARFGAIF--PTNRLRELSAMRLAVDYVNEKDIV---PGVTLQYLLSTAESLDHFEMVLKGCRQTE------ 85
         Nem 5500937 MR-----------------GWRAALRV-ALIVSLVECACVNSTQSAP-TEIKIGGIFC-QENNNHEYKAFNLAIEYINNDTSIL--PGTTLVPMTNSTKWLNAFNNIEAVTWQIF------ 93
       Nem 5511254 db MY--------------------------LPFLALFFCFSIASVKAQGKRILKIGGIF--EKENDIDDRVFRFAVDWVNANNIT---KDVVFEPVVSYASQYSSYKNIESASKLIS------ 84
         Eup AQX17743 MV---------------CMNSQKGCSLV-VILLLLFANAEPP-------EEIRIGLLS--HEGLQNVEAAFDYAIKLINEDWTVLSRTNVVGFKLKMKSAEDSPSEVVRNILDLVS------ 91
                                                                                          ...Extracellular N-terminal domain...
            Rat GluA2 -RGVYAIFGFYDKKSVNT-ITSFCGTLHVSFITP--SFP----TDGTHPFVIQMRPD---LKGALLSLIEYYQW----DKFAYLYD-SDRGLSTLQAVLDSA-AEKKWQ----VTAINVGNI 183
  Lin GluK-XP_013416771 -NGVGAIFGPMATISASH-VQSICDTLEVPHIETRWDY-----KEFKDYYSVNLHPHYTSIGKSLLDLVTHWKW----GAVTILYE-DNNGLLKLQDLLRVP-GKKKSL-QTTIRKLEAF-- 197
   Hof GluK-98026516 PSGVVAILAPPPAESAAA-VQSIGDAFEIPVLHYGQDYQ----PTSLNSLYLNLYPHHTSIGRALFDVVSYYNW----TSFALFYQ-DKLGLRRMEPLFQFP-KKPNTS----FVAVKL--- 218
            Aca GluD -TTVAALVSSTSCETSLA-LQSLANSFDVPHIIVPGEEC---LSEKHNSFTVNVRPSQVYLSEAVLDLVWWLKW----KSVSMFYD-SESAYKNVQQFLHLA-AQSEERKPLEVTLYRVDDR 204
       Nem 5513056 db -EASCIFDLSFGDERSFA----LAELLRVPMVVLRPIYG----SRWKSEFVFTMQPPEDSTAMATVNLLHYLTKESNVKKVAILAE-RKRSHLGARIFSLLR-RRERVM-P--YMMPEL--- 190
       Exa 110238381 -KSTFIFDLSFNNQYSYA----LAELLQTPMITLETMH-----DNIRNYFIFSLRPSEETVARATVDLVQYYMKFN--AQVAILVD-AKMSHLGARIFSIAR-YRNRIR-P--IMMPEA--- 191
         Nem 5503753 -YDVIALLVDAHPQPS---QQAMSAMFRIPMVTLHEAHTYTSQTQSLLQYGICVGPSGYDVTRAMNDVITHLNR----NKILVLYD-GRWRSQAMLFISRLP-SNSSYS----KMSLEI--E 203
          Tri 19383# -NGIVAIIGSLRNPQSMAIAESIFYHYTIPHLAVNSVLH--SKYIGSESYSFHLKPSKLAELEKIFTILEIHEY----RRIAVLYD-QTCDSGELVSVLQLA-HRREVN----VQLEEMSDW 200
       Tri 18262 (E3α) -DKVWAIIGPLTSSDVEA-VQYMATSLRVPQVAPIATYP-GFDDRPSMGYLVRMSPSDKWQSRVLADIFQRFHW----SYAAIIVSNTDYGINSVSEFRNVA-AGRDWK----ILAFETFNT 204
 Bra XP_019617116 sp (E6) -QGVVAIMGPILSSQVKA-SSPVSTGLSLPQIAPEATDP-TLGNAQQYPQLIRISWPDSVLSKALIDLLEHFSW----DQMSIFVSNDDYGVHGLVDFQLIA-GENGWR----IHTIQSFDP 196
 Bra XP_019616632 db (E8) -QGVVTIIGPMTSSQVKA-SSPVSSGLSLPRIVPEATDP-TLENTRQYRQLVRISWPDSVLSKVLIDLLEHFSW----DQMSLLVSNDDFGVHGLLKFQLLA-GQKRWR----IHTIQSFDP 192
    Bra XP_019645125 sp -EGIVTILGPHGSSQVKA-TQLVSSSLNIPQISYAATDP-YLANQEINEYLLRMSPSDASQGLALAELIEHFGW----SQMSIIMSTDDFGTHGIVEFEKLA-GMKGWL----IRSVQGFTP 195
         Nem 5500937 -HQAVAIVGPLTSPMVRA-TQPLCSGFHVPQVAPYATDPAFEFSPSSYKYLLRMRSSDSIENRAIADFIGHFNW----TRLGLFTSRDDYGLNGVAAIKDIA-SRMGWV----IAAVDSFRQ 204
       Nem 5511254 db -QGVLATVGPMTSPDVKV-TLGLFKRFKIPLLAPSATDTAFIYDNYRYKFLLRMLTHNAAQDIALVDLINQFKW----ERMGILTSRTDYGMNGLTDFKEYGVGQFKWV----ILATEHFAV 196
         Eup AQX17743 -NKVSAIVGPFRSSHATI-ASYILTELRIPMITPTSTDP-FLRNPLWSSYLLHMQPLDNYQSEALLDLVKYMDW----NSVALISSMDSYGINGLTTFQKYS-NERKIK----IMANEMIPL 201
                                                                      ...Extracellular N-terminal domain...
            Rat GluA2 NNDKKDETYRSLFQDLELKKERRVILDCERDKVNDIVDQ---VITIGKHVKGYHYIIANLGFTDG------------------DLLKIQFGGANVSGFQIVDYD-DSLVSKFIERWST---- 279
  Lin GluK-XP_013416771 ----EDGDYRPLLKDLKSQNQFRFIVDCDYKKIPTLLAQ---AMSLSMLSQYYHFHFTSLDLDLL------------------DLGDYRYGGANLTGFRLIDWD-DPEVKIAMLDLAS---- 289
   Hof GluK-98026516 ----NEDTSMQILKDFRKTDRQRIIVDYQSNYLIDFLQK---LQKMQMLSEYHHYIFSSYELSSE------------------SVENFTYGDVNFTTFQIVDFR-KQKYIDHLDQFKK-YLS 313
            Aca GluD DPASGALGMINILHKAKDSDVQHMITFCDRAQSMRLIRQ---AARFGMAVGKYQWIITTQDWDMMGDGEPGSFTTAGGDISEEEGMRFLNGSEGIITLMKQQVKVQTHPLQFYSAWQMLYPD 323
       Nem 5513056 db -VIGDDSQNTKALTELLRSGLSYVILVCPPKDMRALMEK---AMRLGCVNKESKWITANMEIPGN------------------ISALALRG---LLGIRLHATDTGRLRREVGNNR------ 281
       Exa 110238381 -NTKNEIETQRALSVLLRTNSRNVILVCKAEDTRQFIER---AMRNGCVFSDSKWFVVDLELTGN------------------ISALAYRG---LLGLRLYYNNQHKLKRDAEKSRNG---- 284
         Nem 5503753 SDSWLCGDTKELLGRLNNAPTEVIALFCDSYLVAEVIDKVHCACSFKVEKP-HIWLIADMIPSVS------------------MPVQSSQV---VLGFLPQVTD-SYLTRHIDRELNL---- 298
          Tri 19383# NYTTKSVAISIIINRINRADIRVAYLCVDRTLIDDIVTY---VVRQDMHTFKHTWIIKDEVFQDA-----------------EDDFASLKR---LYSIGYSPDT------KMEQKWKN---- 289
       Tri 18262 (E3α) EETNNFPIIDDHLMKVKNSGAKVIILSCLSDQAAIVIKR---AHRLGLTGNGFQWVLFDNTITEG------WLNL--------DPPGYLTG---AIGIMSSNDK-GELYGQVETAWKR---- 301
 Bra XP_019617116 sp (E6) TENAADIVVQSQLGVIKDTGARIIVLHCLASYAAEILRV---ASTMDMTGAGWAWVVSDGITGLD------LFDT---TNGTANVPDYLQG---LLGPIPPSTN-GAHSQDFMTKWRA---- 298
 Bra XP_019616632 db (E8) TEIAVDIDVRTQLKVIKNTGARIIILHCLASYAAEVLRQ---ASTMDMTGEGWAWVVSDGITGFD------LYDN---DNGKAVVPDYLRG---LLGPIPPATN-GAHSEEFMAKWRA---- 294
    Bra XP_019645125 sp TKNAEDLDISQQLRAIKSSGARIIILNSVVEYGKVILKQ---ANDHGMVGAGWQWIVTVGITGSI------LFP------GVEDMPDYYEG---LLGVYIVDDA-GELKGEFLRRWMA---- 294
         Nem 5500937 FEDPLRVNATQQLVQLRARGIRIIILNCLASYARVILKQ---ASELNMIKD-YVWIVKNGAFSFK-----GLFD------SEDNVPDYMQG---VVGMRTSFRG-GVLQDEVKRAWVS---- 303
       Nem 5511254 db PTDYTKINATKQLLRLREKGTRVVILNCMSKYVEYIMKQ---AERLDMLNE-WVWILTDGAIAKE------------------GSTKYKEG---LIGVRYPQTGKGRLSGPVNKAWSA---- 289
         Eup AQX17743 TKNMSRLDVSQQVKNLKDSEARIFIMNVHSGYAKPVLDI---AQQMGITGPDYAWVVTDATIKDA----DGLIN------KYNEFPEAIQG---MVGYMSNVCC-SKKKAEFMDKWRTESGG 306
                                                      ...Extracellular N-terminal domain...
            Rat GluA2 ----------LEEKEY-------------PGAHTATIKYTSALTYDAVQVMTEAFRNLRKQRIEISRRGNAGDCLA--NPAVPWGQGVEIERALKQVQ-VEGLSGNIKFDQNGKRINYT-IN 374
  Lin GluK-XP_013416771 -------PKALPGTPF-------------VGAVQTKTKVQAAVMVDAVMVFARAITDL-SRSQTIQTRSLG--CS---NPGQTWTQGSSLFNFLTRVG-LTGLTGRIKFDGDGKRRDFD-LG 383
   Hof GluK-98026516 SQASGQTKLRTDPLMY----------------NDKLITLEMALIIDAVNFLQSSFESM-ANVRDIQSQSLR--C----DSDTSWSHGYTVMNYMRQMKYFEGLSGNLLFNPYGQRVEVF-TD 411
            Aca GluD IDR----EEDANSTYL-------------SPGYLNTIQFKAAYMYDAVRVLAVALNEQVERDKYIEPEIQH--CYD--NKPKSWKGGIRLRKMLHRTE-TTGLMGRMRFNHSSLNDEIA-VD 422
       Nem 5513056 db -------------------------------GYPEITGLTYAVLNDSMYVVGLAIRKMMLSPIIVDETKSNKYCPK--NLVTTPGKRHNILNFLSKVN-YQGFTGPVVFKQGSNERVVQIFD 369
       Exa 110238381 -------------------------------RAKKISMASYAVLNDSLYAVSEAIRQT---KQITFDNRGKDDCPI--KGAMDDRGQSNFINSLMGVR-FEGLTGPVEFDQKTRTRNVKIID 369
         Nem 5503753 --------------------------------SKTAIYNSVYFAYDAVLATAHALDKVILEDRWVSLNRSH--------GLTTSLNGPSIFEEMKKLK-INSTTGILDIAHDGSRRNLE-LE 378
          Tri 19383# -------------------------------------VRVEQAISYDAVHIIAKAISST-IQLNQFRHQKIN--CNR--HPLIPLTSGHAIMRQVKKLY-FSGATGKIEFNDRGERLHWN-PK 368
       Tri 18262 (E3α) -------------------------------KYKTSLEPVFLRYYDAVIAIAYGLRESFSQLQSIAPIKLS--C----LNLSKWENGQILLQNIVKSK-GPGSSGPIRFDARHAPYNPN-YE 384
 Bra XP_019617116 sp (E6) ----------ADPAVY-------------PGAGVGDIGPYTARWADAVLALAQALRNLQEDGVTVTPQPLD--CGCDGSDSQPWADGPTMLQYLKQVE-TDGVTGNIRFDSTSARLDAE-YN 393
 Bra XP_019616632 db (E8) ----------ADPAVY-------------PGAGGADIGPYTARWADAVLTLAQALRNLQTDGVTVTPQPLD--CACDGGESQPWADGPTMLHYLKQVE-TDGVTGYIRFGQTAARLDAE-YN 389
    Bra XP_019645125 sp ----------ADPVLY-------------PGVGNGTMVAYAGKAMDAVLLLAYGLRDMLAAGETVDTESLN--CSL--IPHPEWSRGETFLQYLKQVD-GPGVTGRVSMKSDGSSRNAV-YD 387
         Nem 5500937 ----------------AG-----------YGEMAIENEDAVGHTFDAVLVLAHALHNMLNDGHNISNVQPQ-FGFYDGLSTEPRPDGATLLDYISQVN-TTGVMNQLGFDSNRSPVDVA-FD 395
       Nem 5511254 db ----------------------------R--KEKTKIEGRTGRIFDAVLTIAYGVNLLRDNVTITDPPKGDSFCSSSNLTAQPWSLGEQFYNYLKEVK-GDGFMHQIAYSPMGQPRARS-FD 379
         Eup AQX17743 VQA--KVSFPADQMRYWSGNTESTPTDNDESELRSQWKSRASLTYDSVWVIAQAINKL-AYLDNIPLSPPN--VDYWSSPVRYFNEGQTILDYISRVQ-LDGITGMIEFDATGGPVRKL-YD 421
                                      ...Extracellular N-terminal domain                       Segment one (mostly the upper lobe) of ligand-binding domain (S1)...
            Rat GluA2 IMELKTN-GPR-----KIGYW-----SEVDKMVVT----------------------LTELPSG-NDTSGLENKTVVVTTILESPYVMMKKN---HEMLEGNERYEGYCVDLAAEIAKHCG 458
  Lin GluK-XP_013416771 IVKMGKR-TLE-----RIGFW-----TLEGGVNITAN--------------------FTELKDQ--LHDTLQNKTLIVTTIQEPPYLMLRKAQ-PGVELKGNARFYGYCVDLLAEISKLLN 470
   Hof GluK-98026516 ILEVRSK-DLA-----VVGNW-----SQSTSVNITT---------------------LNEIINV---QELLHNRTLRITTILNEPFTFWKEAE-HDKQLKGNDRFDGYCIDLLKKIKEQLG 496
            Aca GluD VITLESG-RNQT-KAWKIGGW-----DPENRLNLVK---------------------TPFSRGF--AAFISNNTTFRIVTVVEAPFVNRDET-------VNGYKYSGFCIDMLELIAKELN 506
       Nem 5513056 db ILNLQLQEDEQTTRKIRIGHLVNN-ETRASKITMDTEEKIFWNGEYWGDIQCEFRGKETIKAPPV-------QHKVLIVTVREDEPFMMIKPN---ASLLKGNDRYRGFSKDLMELLSRKLN 480
       Exa 110238381 AVNVQIKAGFPPSFMYPIGYWKPNVDSPIGSVVLKTNDAVFWNGDYWEEIKCSF----TSNSPSFMTNDFVRHHKPLIVTSIVDSPYMMLKKN---HSGLTGNDRYEGFGVDMISKLAEKLK 484
         Nem 5503753 VFNLQND-IFT-----KIGVW-----SASSGVTVSG--SVKQNRP-------------TTDLVPL-----DKLHRKLEVVVVLDSPFVMLNEA----------GVLEGFSIDLIKEISQMLG 459
          Tri 19383# IHDVHSR-MKQ-----SSISK-----REKRSVLAASESNDNGESS-------------NPLSPQN---LQFMANETLRITTVIDAPFIMYKPE---NQRGPGIDAFEGYCIDLLKLLVAELG 460
       Tri 18262 (E3α) VVNLVNK-TWK-----SIGSW-----NKVSGATINGV-AVKFMDN-------------IHDSRKF---DTGLQGKHFNVTTKIDPPFLNYKAD---WAKLNGNDRYEGFITDLLNRLATSLK 475
 Bra XP_019617116 sp (E6) IVNLKSD-GWQ-----KVGTW-----NLTARLDIHPDADIRFSGG-------------ATTVVPY---MSDLSNRTLRVVTIAADGFVEISDVDRDGNNVTGNDRFQGFCMDLFSWMSTELG 488
 Bra XP_019616632 db (E8) IVNLKSD-GWQ-----KVGNW-----NLSTGLNIHSDADIRFAGG-------------ATEVAPY---VSDLTNKTLRVVTIAADGFVMISDTDENGKNVTGNDRFQGFCMDYFKWLSDELG 484
    Bra XP_019645125 sp VVTLDKH-RWK-----KVGRW-----NDSSGLTLPE--SVTFMGG-------------QQEVIDF---ISDLSNRTLRVVTHKEQGFVHIRDTDDWGKNLTGKARFSGFCIDLLEWLSEKLG 480
         Nem 5500937 VVNLRAF-GFQ-----KVGYW-----NVEEGLHLDNKKEIVWPSG-------------RVYVPTD--STHILENRTLKVVTIAEAPFIFAQTQT--NGQGETRVIIEGYCIELLRKLSEMLR 489
       Nem 5511254 db IVNYQTEDGWV-----KVG-Y-----SIGLRVKLEKKRPVIWLNG-------------GIEVPKL--ASLALKYKKLKVLIFESRPFVMAKTGN--ISTSDNKVLYEGFCIDLLEELKKKLS 473
         Eup AQX17743 LVNFRVN-DVV-----KVGEW-----SPNIGLKMNQTEQILWNGD-------------SKLTPSS--SVQSLDGRTLRLGITQEPPFISIDTNC--TAEGRGNECYTGLCIELVQKLAKQLH 515
```

Figure S1. Amino acid sequence alignment of 15 iGluRs investigated here. Continued overleaf with full caption.

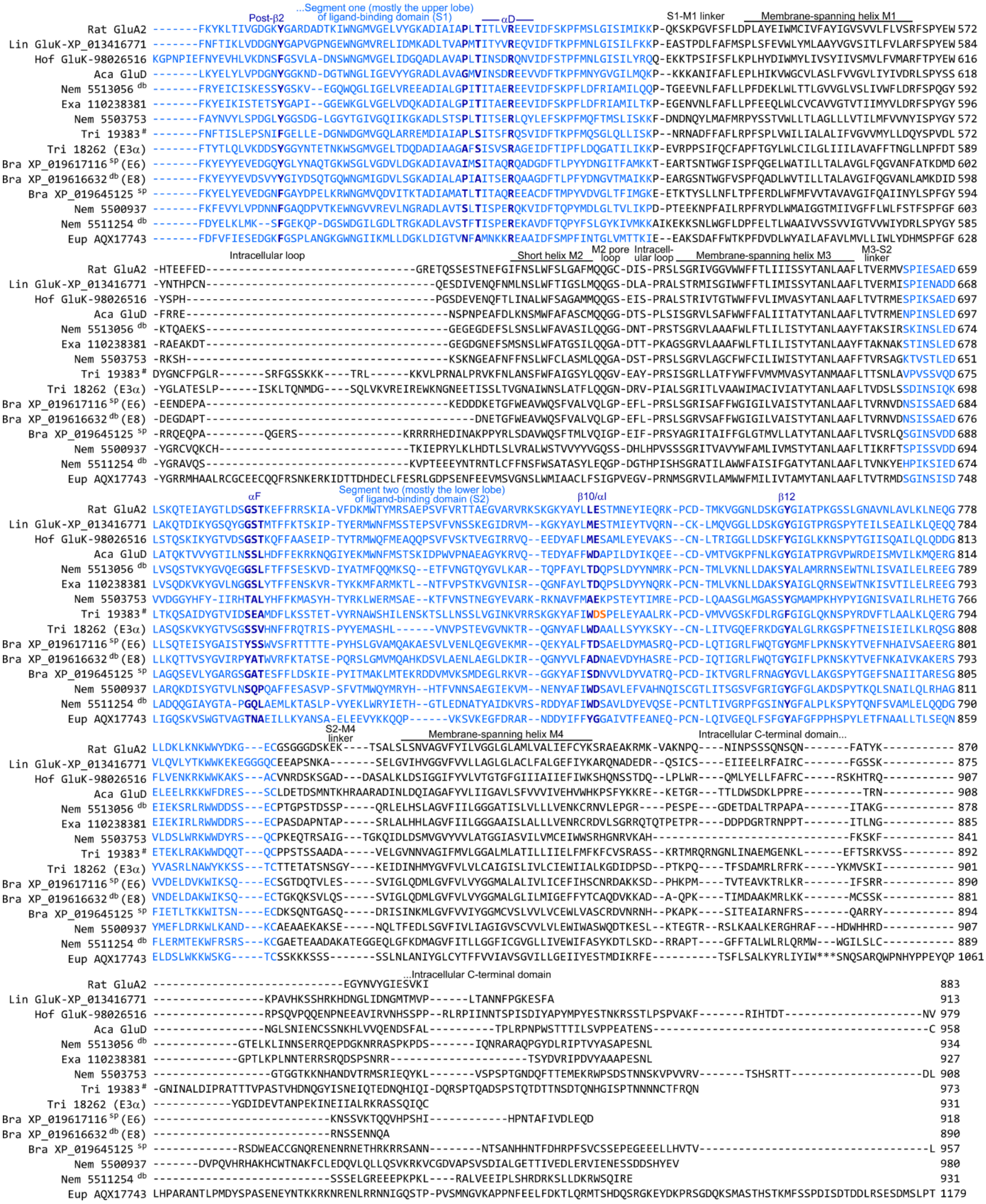

Figure S1. Amino acid sequence alignment of 15 iGluRs investigated here. Lin, *Lingula anatina*; Hof, *Hofstenia miamia*; Aca, *Acanthaster planci*; Nem, *Nematostella vectensis*; Exa, *Exaiptasia diaphana*; Tri, *Trichoplax adhaerens*; Bra, *Branchiostoma belcheri*; Eup, *Euplokamis dunlapae*. Ligand-binding domain (blue font) and secondary/tertiary structure names (αD, etc) inferred from rat GluA2 receptors[1,2] – numbering of α-helices and β-strands, and precise length of membrane-spanning helices probably differs in other receptors. Residues discussed in main text in bold, dark blue. [sp, db] Certain cDNA sequences were designed to include signal peptides and N-terminal sequence (green font) from other receptors to enhance expression (sp) or were based on transcripts from other databases than the one we used to identify and name the receptor (db), detailed in Table S2. [#] The *Trichoplax* 19383 receptor used is a two-residue mutant (orange font in alignment) that is activated by the same ligands that inhibit the constitutively active wildtype 19383 receptor (Fig. S3D). *** in second-last row of alignment, 105-residue segment in C-terminal domain unique to *Euplokamis* AQX17743, not shown for brevity.

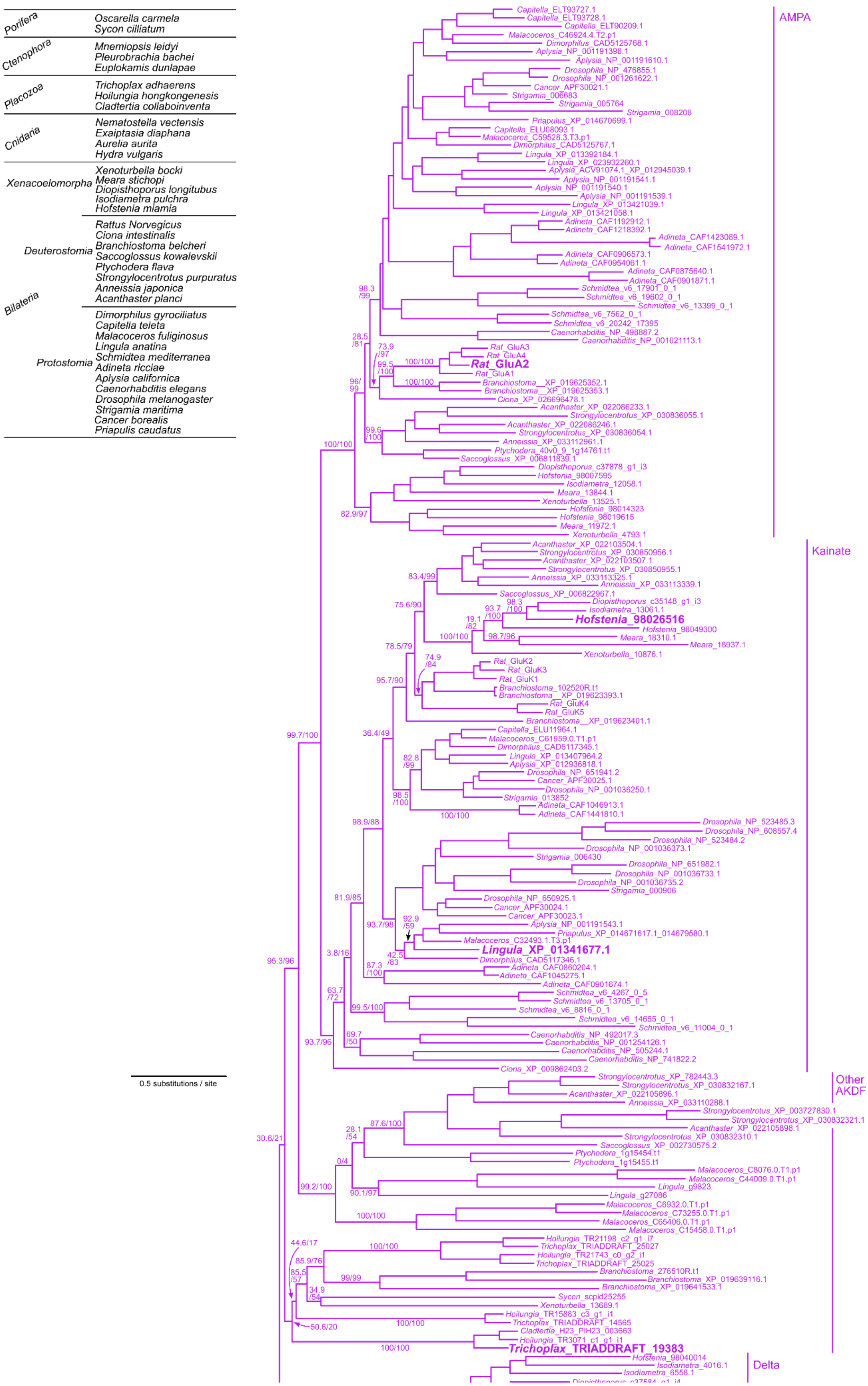

Figure S2. Animal iGluR gene tree. Continued overleaf with full caption.

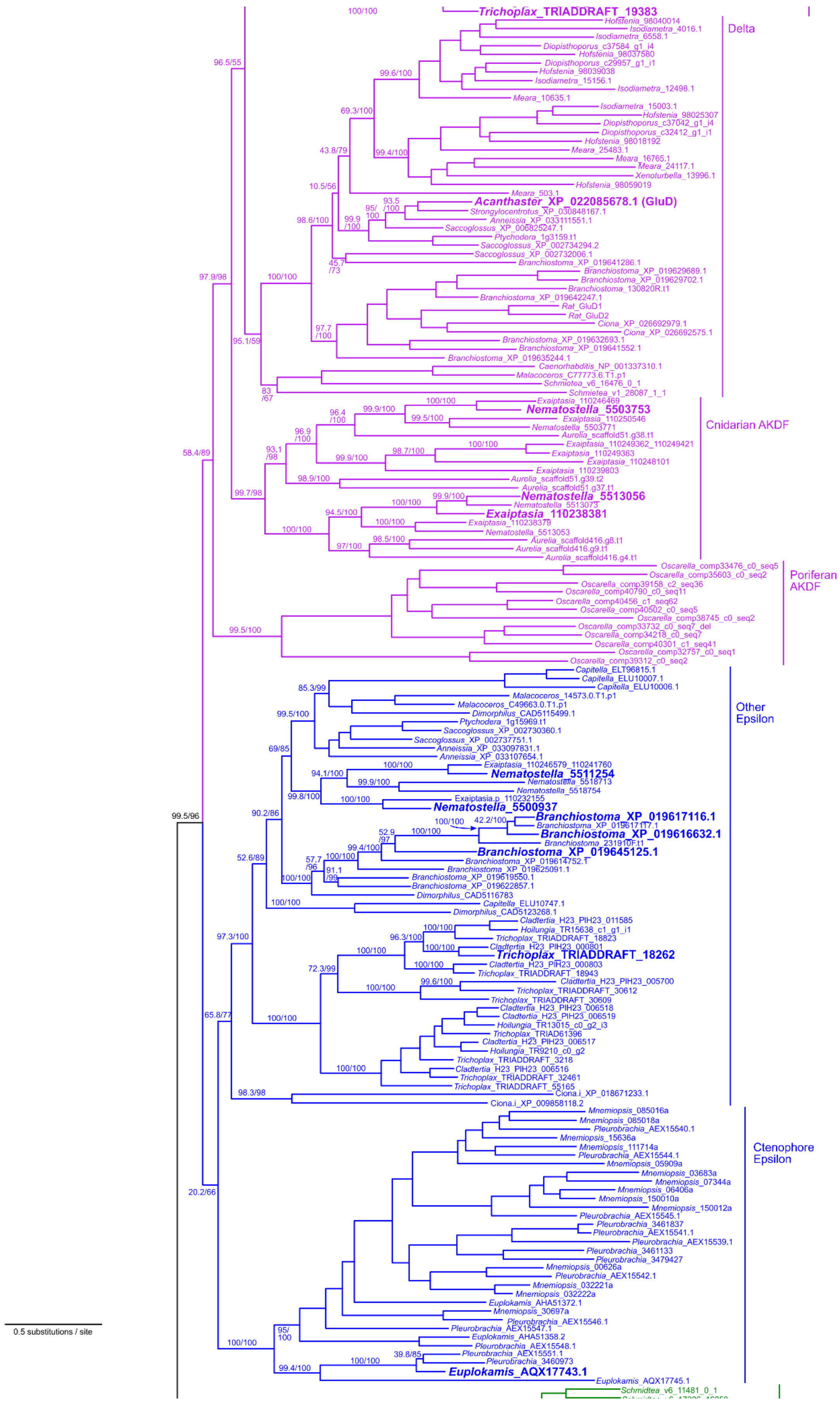

Figure S2. Animal iGluR gene tree. Continued overleaf with full caption.

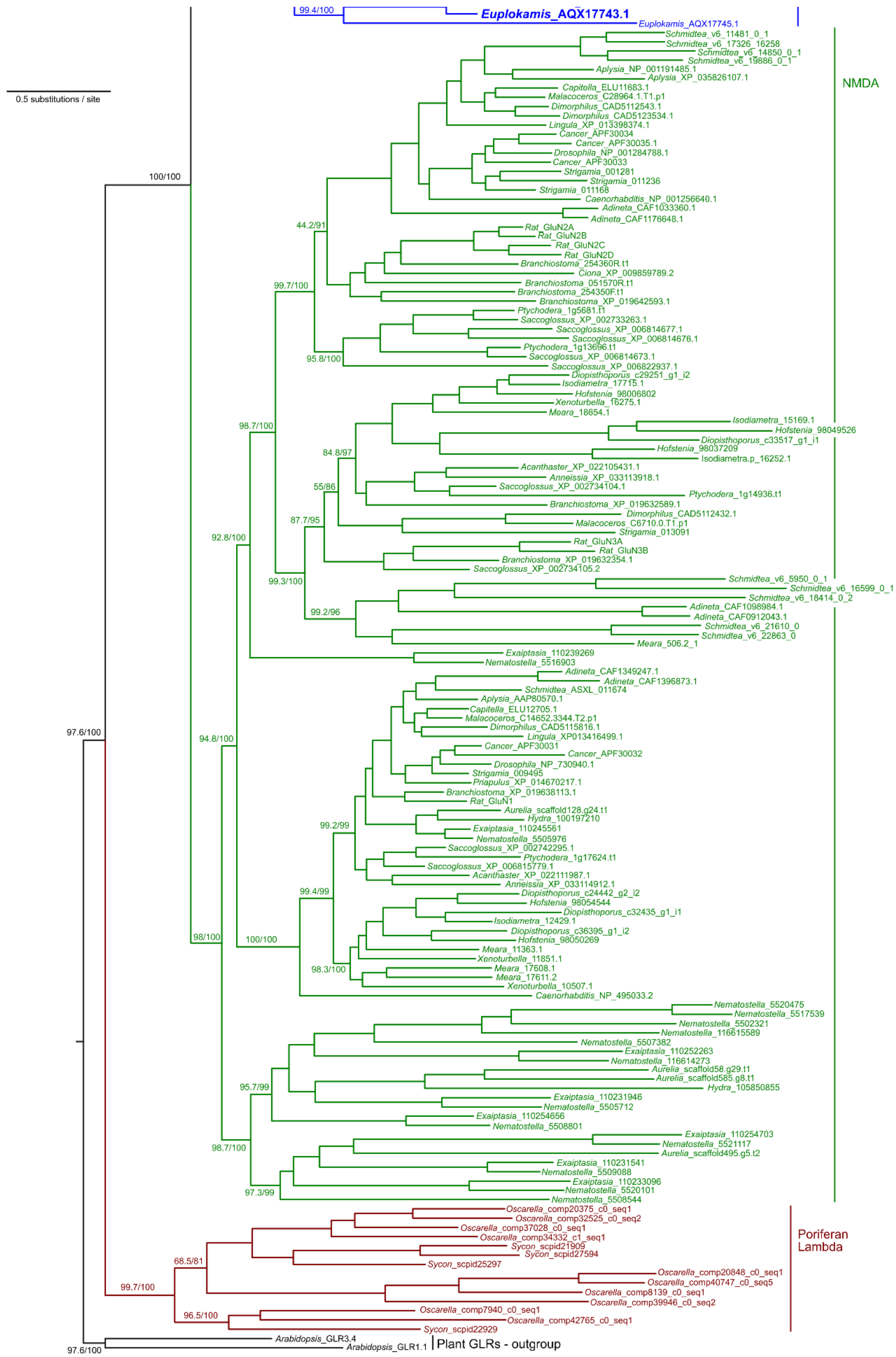

Figure S2. Animal iGluR gene tree. Maximum likelihood animal iGluR gene tree generated in IQ-TREE using a LG + I +G4 substitution model (*Materials and methods*). Full species names in table at top-left. Branch support values, separated by slash, are SH-aLRT and ultrafast bootstrap branch support. For visual clarity, only selected branch support values are shown; tree with all branch support values freely downloadable from https://zenodo.org/records/22765788 . Selected subfamilies colored and labeled at right. Genes studied here in bold font.

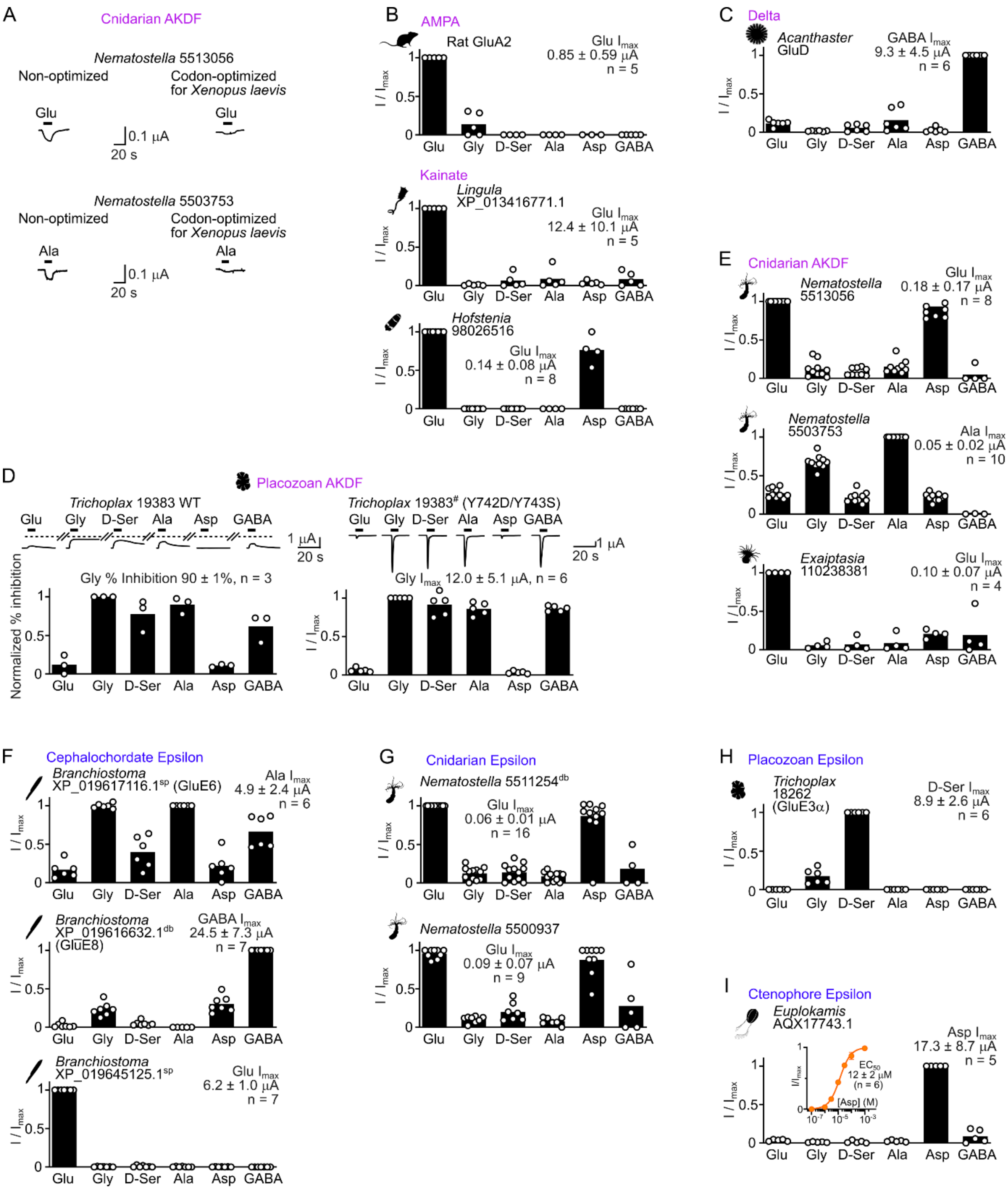

Figure S3. Heterologous expression and experimental characterization of diverse iGluRs. A, two-electrode voltage clamp recordings of currents in *Xenopus laevis* oocytes injected with indicated cRNAs. **B-I**, summary data (columns = mean) of normalized ligand (10 mM) -gated peak current amplitude ("I/I_{max}") corresponding to Fig. 1C-K. Absolute peak currents for all ligands given in Table 1. **D**, Comparison of ligand activity at *Trichoplax adhaerens* AKDF19383 wildtype (% inhibition of leak currents by ligands) and Y742S/Y743S double-mutant (ligand-gated inward currents) receptors. **I**, *inset*, mean, normalized current responses to increasing concentrations of aspartate for oocytes expressing indicated receptor. EC_{50} is mean ± SD from fits to data from 6 cells.

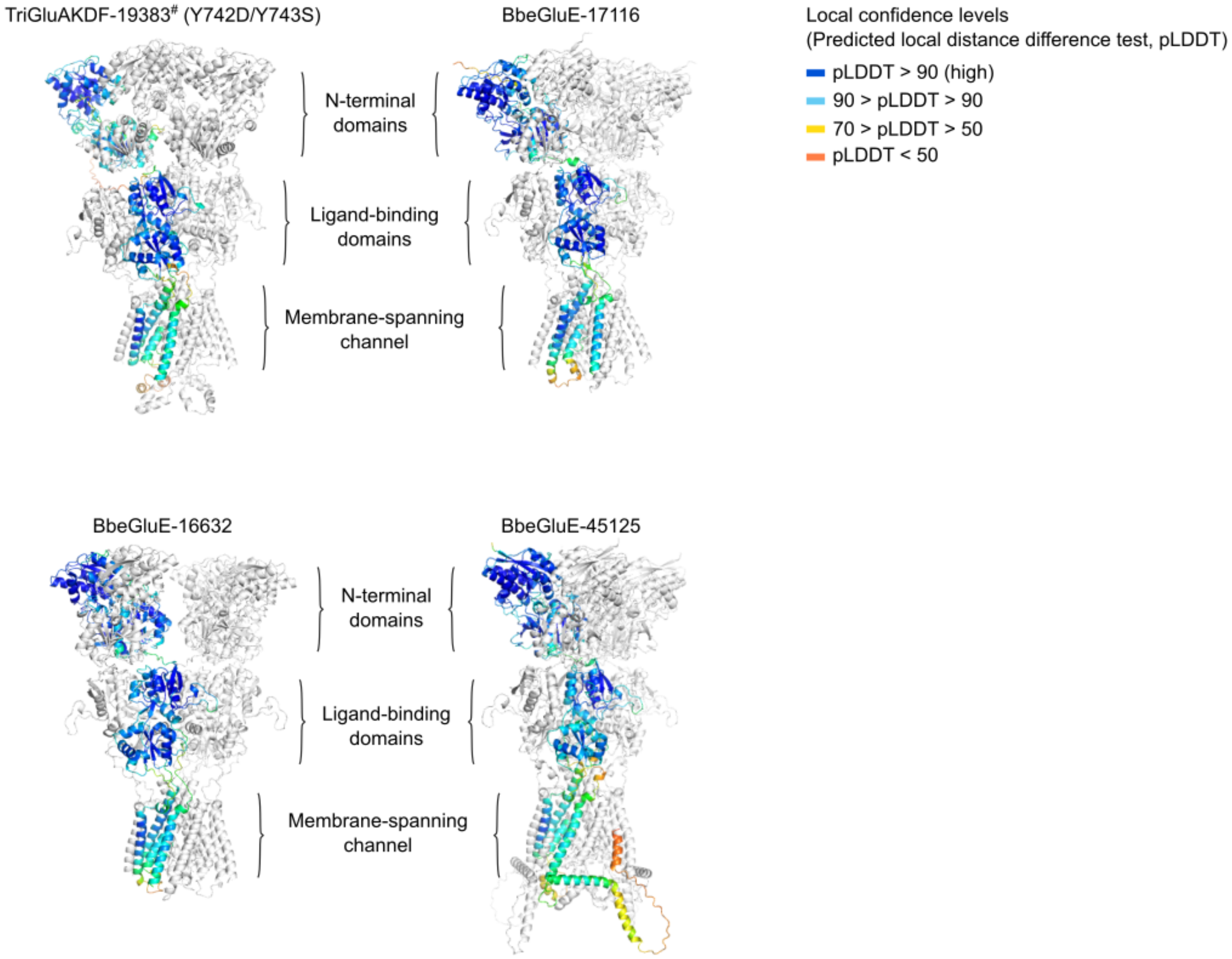

Figure S4. New AlphaFold3 models. Three subunits in each homotetramer colored white, one subunit colored by pLDDT score, indicating local confidence in structural prediction.

Table S1. Databases from which diverse iGluR sequences were retrieved

Lineage and species	Database
**Ctenophora**	
Mnemiopsis leidyi	ENSEMBL Metazoa
Pleurobrachia bachei	NeuroBase [3] & NCBI/GenBank
Euplokamis dunlapae	NCBI/GenBank
**Porifera**	
Oscarella carmela	Compagen
Sycon cilliatum	Compagen
**Placozoa**	
Trichoplax adhaerens	Published genome[4]
Hoilungia hongkongensis H13	Published genome[5]
Cladtertia collaboinventa H23	Published genome[5]
**Cnidaria**	
Nematostella vectensis	KEGG, NCBI/GenBank, NvERTx[6]
Exaiptasia diaphana	KEGG
Hydra vulgaris	KEGG
Aurelia aurita	OIST [7]
**Bilateria - Xenacoelmorpha**	
Xenoturbella bocki	Published transcriptome[8]
Meara stichopi	Published transcriptome[8]
Diopisthoporus longitubus	Published transcriptome[8]
Hofstenia miamia	Published transcriptome[8]
Isodiametra pulchra	Published transcriptome[8]
**Bilateria - Deuterostomia**	
Rattus norvegicus	NCBI/GenBank
Ciona intestinalis	NCBI/GenBank
Branchiostoma belcheri	LanceletDB[9] & NCBI/GenBank
Saccoglossus kowalevskii	NCBI/Genbank
Ptychodera flava	OIST [10]
Anneissia japonica	NCBI/Genbank
Strongylocentrotus purpuratus	NCBI/Genbank
Acanthaster planci	NCBI/Genbank
**Bilateria - Protostomia**	
Dimorphilus gyrociliatus	NCBI/Genbank
Capitella teleta	NCBI/Genbank
Lingula anatina	OIST [11] & NCBI/Genbank
Adineta ricciae	NCBI/Genbank
Aplysia californica	NCBI/Genbank
Malacoceros fuliginosus	Published transcriptome[12]
Schmidtea mediterranea	PlanMine [13]
Drosophila melanogaster	NCBI/Genbank
Cancer borealis	NCBI/Genbank
Priapulus caudatus	NCBI/Genbank
Caenorhabditis elegans	NCBI/Genbank
Strigamia maritima	ENSEMBL Metazoa

Table S2. iGluR synthetic cDNAs used for expression in *Xenopus laevis* oocytes

Blue, restriction sites; pink, spacer and/or Kozak consensus-like sequence;[14] purple, C-terminal c-myc tag; green, homologous sequence from other genes, as described below; orange, other mutations, as described below.

Lingula anatina XP_013416771.1 (GluK)

Based on XP_013416771.1 from NCBI/GenBank. Codon optimized for *Xenopus laevis*.

GTCGACGCTGCCACCATGAATACCGTAAGGATCTTTGGAGTCCTCGCTGGACTGTGCCTTAACCTAGGTACCTTCATCCTGGGGCAAGACACACGCCGGAT
CAAGATCGGCGCTGTGTTCGAAGGAGCTTCTTCGATTGAAGAGCTGGCATTCAAGTATGCAGTAAACAAGGTGAACAGCGAAATCTCTGCCACTCTGAAC
GGGGTAACGCTGGAGTATGAATTACAAAAAGTTACATCTGATAGTTTCCAGACTTCAAAAAAAGTCTGTCGTCTACTAAAGGACGGAAATGGAGTAGGCG
CTATATTTGGTCCTATGGCCACCATCTCGGCCTCCCACGTACAGTCAATCTGTGATACCCTGGAAGTCCCCCACATCGAGACGCGATGGGATTACAAGGAAT
TTAAGGATTATTACTCTGTGAATCTTCACCCACATTATACATCCATTGGGAAGTCACTGCTGGATTTGGTCACGCATTGGAAATGGGGCGCCGTCACCATTCT
GTACGAGGACAACAATGGATTATTGAAGCTGCAGGATTTGCTCCGAGTTCCTGGAAAGAAGAAGTCTCTGCAGACCACAATTCGCAAACTCGAGGCATTT
GAAGATGGAGACTATCGTCCTCTGCTCAAGGATCTGAAGTCCCAAAACCAGTTCCGTTTCATAGTAGATTGTGACTATAAGAAGATACCCACTCTTTTGGCA
CAGGCAATGTCTCTAAGCATGCTGTCACAGTATTACCACTTCCATTTCACAAGTTTGGACTTGGACCTTCTGGACCTAGGAGACTACCGTTATGGAGGTGCC
AATCTGACAGGCTTCCGACTGATAGACTGGGACGATCCTGAGGTGAAGATCGCGATGCTGGATCTGGCCAGTCCCAAGGCTCTCCCGGGAACCCCTTTCG
TCGGCGCTGTCCAAACAAAGACCAAGGTACAGGCCGCAGTTATGGTAGACGCTGTGATGGTCTTCGCTCGAGCTATTACAGACCTGTCGCGCTCACAGAC
CATCCAGACTCGATCTTTGGGCTGCTCTAATCCCGGGCAGACATGGACTCAAGGCAGCTCTCTGTTCAATTTTCTTACACGTGTCGGGTTGACTGGTCTGA
CAGGCAGAATCAAATTTGATGGAGACGGAAAGCGTCGTGATTTCGACCTGGGAATTGTGAAGATGGGGAAACGAACACTAGAACGTATTGGATTCTGGA
CTCTGGAAGGAGGCGTGAATATCACAGCAAACTTCACCGAACTGAAGGATCAGCTTCATGATACTCTTCAAAACAAGACCCTGATTGTTACAACCATCCAG
GAGCCTCCGTATTTGATGCTTCGCAAAGCTCAGCCTGGTGTGGAACTGAAGGGCAATGCGCGTTTTTATGGTTACTGTGTGGACCTCCTGGCCGAGATCTC
CAAGCTGCTGAATTTTAACTTCACCATCAAGCTGGTAGACGATGGAAACTACGGAGCACCTGTTGGACCAAATGGAGAATGGAATGGTATGGTCCGGGAA
CTGATTGACATGAAAGCTGACCTAACTGTCGCTCCCATGACAATCACATATGTCCGTGAGCAAGTGATAGATTTCACCAAACCCTTCATGAACCTGGGGATC
TCCATCCTGTTCAAGAAACCAGAAGCATCAACCCCAGACCTTTTCGCTTTCATGTCCCCTCTTTCGTTCGAAGTCTGGCTATACATGCTGGCTGCCTATGTGG
GTGTCTCCATAACTCTCTTTGTCTTGGCTAGGTTCAGCCCATATGAATGGTACAATACCCACCCTTGCAACCAGGAGTCTGACATTGTAGAGAATCAGTTCA
ACATGCTCAACTCCCTTTGGTTCACCATCGGCTCTCTTATGCAGCAAGGTTCTGATCTTGCCCCTCGCGCTCTTTCCACTCGTATGATCAGCGGCATCTGGTG
GTTCTTTACTCTGATTATGATCTCCTCGTACACTGCTAATCTGGCTGCTTTCCTCACAGTGGAGCGTATGATTTCTCCTATTGAAAATGCTGACGATCTGGCAA
AGCAAACCGATATTAAATATGGATCTCAATATGGCGGTTCAACCATGACATTTTTCAAGACCTCCAAAATTCCAACCTATGAGCGAATGTGGAACTTCATGTC
CAGCACTGAGCCAAGTGTTTTCGTCAATTCCTCCAAGGAAGGAATTGACCGTGTGATGAAGGGGAAGTATGCCTACCTTATGGAGTCCACAATGATTGAAT
ACACTGTGCAAAGAAACTGCAAGCTCATGCAAGTGGGAGGCCTGCTTGATTCCAAGGGTTACGGAATCGGCACACCACGTGGATCTCCTTATACAGAAAT
TTTGTCAGAGGCCATCTTGAAACTCCAAGAACAACAGGTCCTGCAGGTCCTGTACACAAAGTGGTGGAAGGAGAAAGAGGGAGGAGGGCAGTGTGAA
GAGGCTCCTTCTAATAAGGCCAGTGAACTGGGAGTTATTCATGTGGGTGGAGTGTTCGTAGTACTGCTGGCAGGACTGGGGCTGGCATGTCTGTTCGCTT
TGGGAGAGTTCATCTACAAAGCACGTCAGAATGCTGATGAAGACCGTCAATCCATCTGCTCTGAAATCATAGAAGAACTGCGTTTTGCCATCAGATGCTTC
GGATCTTCTAAAAAGCCTGCTGTACATAAGTCCTCCCATCGTAAACATGATAATGGCCTCATAGACAATGGCATGACAATGGTTCCCCTGACGGCTAATAATT
TTCCAGGCAAGGAGTCCTTTGCTTGAGGATCC

Hofstenia miamia GluK-98026516 (GluK)

Based on transcript 98026516 from the transcriptome SRX503688.[8] Equivalent to Codon optimized for *Xenopus laevis*.

GTCGACACCATGGAAAACCGCAAAAGCCTGCTGCCGAAAAGTGCTATTAAAGTGCGCAAAAAATCCCTGGAACCGTTTGGTTTTCCGCTGGCGGTGCTG
TTCTGCGTGCTCCTTCTGCCGAACTCAGTCCAGAGCCAGACCCAGCAGACCCTGAATATTGGCGCAATTTTCCCTGATACCAGCCCTTCCTCCCTGAGCCA
GCTTGCTTTCCAGTATGCGGTGCAGAATGCGAATGAACGCTCCGGCAGCAACGTGAAGCTGCGCGCATTTACCAAAAAGATCGATATTACCGATAGCTTTC
AGGCTAACAAGGCGGCCTGTGAACTGCTGAACGCGGCGGAGGGCACCAGCCCGTCCGGCGTTGTGGCTATTCTGGCTCCGCCTCCGGCTGAAAGCGCT
GCGGCGGTGCAGAGCATTGGCGATGCTTTTGAGATTCCGGTTCTGCATTATGGCCAGGATTACCAGCCGACCTCCCTGAATAGCCTGTATCTGAATCTGTAT
CCACATCATACCAGCATTGGCCGCGCTCTGTTCGATGTGGTCTCCTATTATAACTGGACCAGCTTCGCGCTGTTTTATCAGGACAAACTGGGCCTGCGCCGC
ATGGAACCACTTTTTCAGTTTCCGAAGAAACCGAACACCTCCTTTGTGGCAGTCAAACTGAACGAAGACACCAGCATGCAGATTCTGAAAGATTTTCGTA
AAACAGATCGCCAGCGTATTATTGTGGATTACCAGTCCAACTACCTGATTGATTTCCTGCAGAAACTGCAGAAAATGCAGATGCTTTCCGAATATCATCATTA
CATTTTCAGCAGCTATGAACTGAGCTCCGAGAGCGTCGAAAACTTTACCTATGGAGATGTCAATTTTACCACCTTTCAGATTGTGGATTTTCGTAAACAGAA
ATATATTGATCATCTGGATCAGTTTAAGAAATATCTGAGCAGCCAGGCGAGCGGCCAGACCAAACTGCGTACCGATCCGCTGATGTATAACGATAAACTGAT
TACCCTGGAAATGGCGCTGATTATTGATGCGGTGAATTTTCTGCAGAGCAGCTTTGAATCCATGGCGAACGTCCGCGATATTCAGTCCCAGTCTCTGCGCTG
CGACAGCGATACCAGCTGGTCTCATGGCTATACAGTCATGAACTATATGCGTCAAATGAAGTACTTCGAAGGCCTTTCCGGCAACCTGCTGTTTAACCCGTA
TGGACAGCGCGTGGAAGTGTTCACCGATATTCTGGAAGTGCGTTCTAAAGATCTGGCTGTTGTGGGAAACTGGAGCCAGTCCACCTCCGTGAACATCACC
ACCCTGAACGAAATTATTAACGTGCAGGAACTGCTGCATAATCGCACCCTGCGCATTACCACCATTCTGAACGAACCGTTCACCTTTTGGAAAGAAGCGGA
ACACGATAAGCAGCTGAAGGGCAACGATCGCTTCGATGGATATTGTATTGATCTGCTTAAGAAGATTAAAGAACAGCTGGGCAAGGGCCCGAACCCGATT
GAGTTCAACTATGAAGTGCATCTGGTGAAGGATAACTCTTTCGGCAGCGTGCTGGCGGACAATTCATGGAACGGCATGGTCGGCGAGCTGATTGATGGTC
AGGCGGATCTGGCGGTCGCTCCTCTGACCATTAACAGCGATCGTCAGAACGTCATTGATTTTAGCACCCCGTTTATGAATCTGGGCATTTCTATTCTGTACC
GTCAGCAGGAAAAAAAAACCCCTTCTATCTTTTCCTTTCTGAAACCACTGCATTATGATATTTGGATGTATCTGATTGTGTCCTATATTATTGTGAGCATGGTG
CTGTTCGTCATGGCCCGCTTCACCCCGTACGAATGGTACTCCCCACATCCTGGCAGCGATGAAGTGGAAAATCAGTTTACCCTGATTAACGCTCTGTGGTTT
AGCGCAGGCGCGATGATGCAGCAGGGCAGCGAGATTTCCCCTCGTGCGCTGTCCACCCGCATCGTCACCGGCACCTGGTGGTTTTTTGTTCTGATTATGG
TCGCATCCTATACCGCGAACCTGGCGGCTTTTCTGACCGTCACCCGTATGGAATCTCCTATTAAATCCGCGGAAGACCTGAGCACCCAGTCAAAAATTAAAT
ATGGTACCGTGGATTCCGGTAGCACCAAACAGTTCTTTGCTGCTAGCGAGATTCCGACCTACACGCGTATGTGGCAGTTCATGGAAGCGCAGCAGCCGAG
CGTCTTTGTGTCCAAAACCGTGGAAGGCATTCGCCGTGTGCAGGAAGAAGATTATGCGTTTCTGATGGAATCAGCAATGCTGGAGTACGAAGTCGCGAAA
AGCTGCAACCTGACCCGCATTGGCGGCCTGCTGGACTCCAAATTTTATGGCATTGGTCTGAAAAAAAATAGCCCGTACACCGGAATTATTAGCCAGGCCAT
TCTGCAGCTGCAGGATGATGGCTTTCTGGTGGAAAACAAACGTAAATGGTGGAAAGCGAAGTCCGCGTGCGTAAACCGCGATAGCAAATCTGGAGCGGA
TGATGCGAGCGCGCTGAAACTGGATTCCATTGGTGGCATTTTTTACGTCCTTGTGACCGGAACCGGATTTGGAATAATTATTGCGATTATCGAGTTCATTTG
GAAGAGCCATCAGAACTCTTCCACCGATCAGCTGCCTCTGTGGCGCCAGATGCTGTATGAACTGCTGTTTGCTTTCCGCTGCCGTTCTAAACATACCCGCCA
GAGACCTTCCCAGGTCCCGCAGCAGGAAAATCCGAATGAAGAAGCGGTGATCCGCGTCAACCATTCCTCCCCGCCACGCCTGCGCCCGATTATTAATAAC
ACCAGCCCAATTTCCGATATTTACGCTCCTTATATGCCTTATGAATCCACCAACAAGCGCAGCAGCACCCTGCCTAGCCCTGTGGCTAAATTCCGTATCCATAC
CGATACCAACGTGTAAGGATCC

Trichoplax adhaerens 19383# (AKDF)

Ligand-gated mutant version, orange nucleotides below. Based on TRIADDRAFT_19383 (XP_002107780.1) from published genome.[4] Codon optimized for *Xenopus laevis*. Published previously.[15]

GTCGACACCATGCCTATATCCAAATTGGTTATCCTGCTTTACATTCTTATTTTCGTCTCCGCTGAGGAAATTCCAATCGTAAAAGTAAATCATAATGTCAT
TAAAATCGGCGTTTTTATGGAAAAATCCTCCATAGAGGGACGAACCGTCAGAGAAACCATTGATTTCATCAACAAGAATAAAGTCTATCCTTTACAGAA
TGCTACCCTGACGCCTGTCTTCCAGGCTATCGATAAAGACTCCCCTTACGTCGTTTCTCGTCAAGTGTGCCAATTTATAGAAAATGGTATTGTAGCTATTA
TTGGGTCTCTGCGAAATCCTCAGTCAATGGCAATTGCTGAATCTATTTTCTATCATTATACAATTCCTCATCTGGCTGTCAATTCCGTATTGCACTCTAAAT
ATATAGGTAGCGAATCTTATAGTTTCCACCTGAAGCCATCCAAGCTGGCGGAGTTGGAAAAAATCTTCACCATCTTAGAAATTCACGAATATCGTAGAA
TTGCAGTTCTATACGATCAGACGTGTGATAGCGGAGAGTTGGTTAGTGTGTTACAGCTTGCACATCGCCGAGAAGTCAATGTCCAACTGGAAGAAATG
TCTGATTGGAATTATACCACCAAGTCCGTTGCTATCTCAATTATAATTAATCGCATTAATCGCGCTGATATCAGAGTGGCTTATCTGTGTGTCGATCGTAC
CCTTATTGATGATATTGTTACATACGTCGTCCCGTCAAGACATGCATACTTTTAAACATACTTGGATTATTAAAGACGAGGTTTTTCAGGATGCTGAGGAT
GATTTTGCTTCACTGAAGCGTTTATACAGTATAGGTTACTCCCCCGATACGAAGATGGAACAAAAGTGGAAGAATGTCAGAGTCGAACAAGCTATCTC
GTATGATGCTGTCCATATTATTGCAAAAGCTATCAGTTCCACTATCCAATTAAATCAATTTCGTCACCAGAAGATTAATTGCAATCGTCATCCTCTGATCC
CACTTACATCTGGACATGCTATCATGCGTCAGGTCAAAAAGCTTTATTTCTCTGGAGCTACGGGTAAGATTGAGTTCAATGATCGCGGAGAACGTCTGC
ATTGGAATCCTAAGATACATGATGTTCATAGTCGTATGAAGCAATCTTCGATCTCTAAGCGTGAGAAGCGTAGTGTACTTGCAGCTTCTGAAAGTAATG
ATAATGGTGAATCTTCGAATCCTTTAAGTCCCCAGAATTTACAGTTTATGGCCAATGAAACTCTTCGAATTACCACCGTGATAGATGCACCGTTTATCAT
GTACAAGCCCGAAAATCAACGTGGGCCTGGAATCGATGCTTTCGAGGGCTATTGCATTGACCTTCTGAAGTTACTGGTTGCTGAACTAGGATTCAACTT
CACCATTAGTTTAGAACCTAGTAATATTTTTGGAGAACTTTTAGAAGACGGCAATTGGGACGGTATGGTAGGCCAGCTGGCTCGTAGGGAAATGGACA
TAGCTATCGCTCCATTATCTATTACGTCGTTTAGACAGTCCGTCATTGATTTTACCAAACCGTTTATGCAGTCAGGATTGCAGCTGTTAATTTCCAAGCCC
AATCGTAATGCGGACTTTTTCGCTTTTCTGAGACCATTCTCACCGCTTGTTTGGATACTTATCGCTCTGGCTTTAATTTTTGTCGGAGTGGTGATGTATTT
ATTAGATCAATATTCTCCGGTCGATTTGGATTATGGAAATTGCTTCCCTGGATTGCGTTCTCGTTTTGGAAGTTCTAAAAAGAAAACTCGACTTAAGAAA
GTTCTGCCTCGTAATGCTCTGCCTCGTGTCAAGTTTAACTTAGCGAACAGTTTCTGGTTTGCTATTGGTTCTTACTTACAACAAGGAGTTGAGGCTTATCC
TCGATCTATCTCCGGACGTTTATTAGCTACATTTTATTGGTTCTTCGTTATGGTTATGGTCGCAAGCTATACAGCGAATATGGCTGCTTTTCTTACCACCT
CTAATCTTGCTGTCCCGGTATCTTCCGTCCAAGACCTGACCAAGCAAAGCGCTATCGATTATGGAACCGTTATCGATTCCGAGGCTATGGATTTTTTAAA
GTCATCGACCGAAACAGTCTACAGAAACGCTTGGTCGCATATCCTGGAAAATTCAAAGACTTCCCTGCTCAATTCATCCCTCGTCGGTATAAACAAAGTC
CGTCGATCCAAGGGGAAGTACGCTTTCATTTGGGATTCCCCTGAACTAGAATATGCAGCTCTGCGAAAACCTTGCGATGTTATGGTAGTCGGTTCCAAA
TTCGATCTACGCGGCTTTGGAATTGGTTTACAGAAGAACTCTCCATATAGAGATGTCTTTACCTTAGCTGCCTTAAAATTGCAAGAAAGAGGAGAAACA
GAAAAGCTGCGTGCTAAGTGGTGGGATCAGCAGACTCAGTGTCCTCCTTCAACGTCTAGCGCAGCTGACGCAGTTGAATTGGGGGTCAACAATGTCGC
TGGTATTTTCATGGTGCTTGGTGGTGCATTGATGTTGGCTACCATCCTTCTGATCATTGAACTTTTCATGTTCAAATTTTGCGTCTCACGTGCCAGTTCCA
AGCGAACCATGAGACAAAGAAATGGTAACTTGATTAATGCTGAAATGGGTGAAAATAAGTTAGAATTTACTTCTCGTAAAGTCAGCTCTGGTAATATC
AATGCTCTGGATATACCCAGAGCGACCACCACAGTTCCTGCAAGTACCGTTCATGATAACCAGGGTTACATATCCAACGAAATACAGACCGAAGACAAT
CAACATATACAAATCGATCAGCGATCCCCAACCCAAGCTGACTCTCCATCCACCCAGACAGACACCACCAATTCCGACACACAAAACCACGGTATTAGT
CCTACTAATAATAACAATTGCACATTTAGGCAAAACTAAGGATCC

Trichoplax adhaerens 18262 (Epsilon, GluE3α[16])

Based on TRIADDRAFT_18262 from published genome.[4] Codon optimized for *Xenopus laevis*.

GTCGACACCATGATGAAGCCCCTGAACTCAAGTTTCCTAGTCATTTACGTTTTCGTAACATTACTGATACGAGCGCCTGTCATAGCGGTACAAGCCAACAGA
ATGGAAATTCCTATAGCTGTCCTAGCAGATGGTCGTGAACAGAAATTCCACGATATCATCCAGCTAGCTGCTGATCACATTAACGGAGATCCTGCTTTCGCTT
TTAATATAATGATAAAGCTGAAAATCTACAATGTGACAAATTATCAACCTTTCCTGTGTCAACAAGAAGGATGTCGTGCTGTCGATGATAAAGTCTGGGCTAT
TATCGGGCCTTTAACATCGAGTGATGTCGAAGCTGTCCAGTACATGGCAACATCGCTAAGGGTACCTCAAGTAGCTCCAATTGCAACTTACCCAGGTTTTGA
TGATCGTCCAAGCATGGGCTATTTAGTTCGCATGTCACCTTCAGATAAGTGGCAAAGTCGAGTACTTGCAGATATCTTCCAACGATTTCACTGGTCCTATGCC
GCTATAATCGTCTCTAACACCGATTATGGTATTAATTCGGTTTCCGAGTTTCGTAATGTCGCTGCTGGACGAGATTGGAAAATTCTTGCGTTTGAAACTTTTA
ATACCGAAGAAACTAATAATTTTCCGATTATCGATGATCATTTAATGAAGGTCAAAAATTCCGGGGCTAAAGTGATCATTCTGAGCTGCCTCTCCGATCAGGC
TGCCATTGTTATAAAGCGAGCTCATAGACTCGGATTAACTGGAAATGGTTTCCAGTGGGTCCTCTTCGATAATACTATTACTGAGGGTTGGCTGAATCTTGAT
CCTCCTGGTTATTTGACTGGTGCTATAGGAATAATGTCCTCCAATGATAAAGGAGAACTATATGGACAAGTTGAAACCGCTTGGAAAAGGAAATATAAAACC
AGTTTAGAGCCGGTGTTTCTGCGATATTACGATGCCGTCATAGCTATCGCATACGGTCTGAGAGAATCCTTCTCACAATTACAGTCTATTGCTCCAATAAAATT
ATCATGCCTTAACCTTTCCAAGTGGGAAAATGGTCAAATCTTGCTGCAAAATATTGTTAAGTCGAAGGGCCCTGGCTCTTCTGGACCAATCCGTTTCGATGC
AAGACATGCTCCATACAATCCAAACTACGAAGTAGTTAATCTAGTTAATAAGACTTGGAAGAGTATTGGGTCCTGGAACAAAGTATCAGGTGCTACAATAAA
TGGTGTTGCAGTTAAATTCATGGATAATATTCATGATTCTCGTAAGTTTGATACTGGCCTGCAAGGAAAGCATTTTAATGTCACAACGAAAATTGATCCTCCG
TTCTTGAATTATAAGGCTGATTGGGCTAAATTGAATGGGAATGATAGATATGAAGGTTTCATCACAGATCTCTTGAACCGTTTGGCTACTTCCTTAAAATTTA
CGTATACATTGCAATTAGTCAAAGATGACAGCTATGGTGGATATAATACAGAAACGAATAAGTGGAGTGGAATGGTTGGAGAACTGACGGATCAGGACGC
TGATATTGCTGCTGGAGCATTTTCCATATCTGTCTCCCGAGCTGGAGAAATCGATTTTACCATACCTTTTCTGGATCAAGGGGCTACCATCCTGATCAAAAAG
CCTGAAGTTCGTCCACCTTCAATTTTTCAGTGCTTTGCACCTTTCACTGGATATTTATGGCTGTGTATTTTAGGACTTATAATTATCCTAGCAGTTGCTTTCTTT
ACCAATGGTTTACTTAATCCTTTTGATACGTACGGTTTGGCTACAGAATCCCTGCCTATATCTAAACTGACGCAGAATATGGACGGTAGTCAACTGGTAAAAG
TGCGAGAAATCCGAGAATGGAAAAATGGCAATGAGGAGACCATATCCTCTCTAACCGTCGGAAACGCGATTTGGAACTCCCTGGCTACCTTTCTACAACA
GGGAAATGATCGTGTTCCTATTGCACTGTCAGGACGAATAACGCTGGTCGCAGCCTGGATTATGGCATGCATCGTCATTGCTACCTATACAGCTAATCTGGC
TGCTTTTCTGACGGTTGATTCCCTAAGTTCAGATATCAATTCTATCCAGAAGTTAGCCTCTCAAAGCAAAGTCAAGTACGGAACAGTATCTGGTAGTTCCGTT
CACAACTTTTTTCGGCAGACTAGAATATCCCCATACTATGAAATGGCTAGCCATCTGGTCAATGTTCCTAGTACGGAAGTGGGGGTTAACAAAACTCGTCAA
GGAAATTACGCGTTTCTGTGGGATGCAGCTTTACTGAGTTACTACAAGTCCAAGTACTGCAATCTAATAACCGTCGGACAAGAGTTCCGTAAAGATGGCTA
CGCACTAGGGCTCCGTAAGGGATCTCCATTCACCAATGAAATTAGCATTGAAATTCTTAAGTTACGACAATCCGGTTATGTCGCGAGTCGTCTTAATGCTTG
GTACAAAAAGTCATCCACTTGCACTACCGAGACCGCTACTTCAAATTCCGGCTACAAAGAGATTGATATTAATCATATGTACGGAGTGTTTGTTTTAGTCCTT
TGTGCGATCGGAATCAGTCTTATAGTCCTGTGCATCGAATGGATAATAGCCCTGAAGGGCGATATCGATCCATCCGATCCGACGAAGCCACAAACCTTCTCT
GATGCCATGCGTCTACGATTTCGTAAGTACAAAATGGTCTCTAAGATTTACGGTGATATTGACGAAGTTACAGCAAATCCGGAAAAAATCAATGAGATTATT
GCCTTAAGAAAAAGAGCTTCTTCCCAGATCCAGTGCTAAGGATCC

Acanthaster planci GluD

Based on XP_022085678.1 from NCBI/GenBank. Published previously.[17]

GTCGACACCATGGAAATACCACAGTGGATAGCTTTAACCTGTCTCCTCCTCATGTATATTCTGGAAGGCGTTGTCACATCTGAGAGGCGTGTTTTCCCGTCA
GTCATAAAAGTTGGTGTAATATTAGAACAGCCGTCGTCAGATGAGGACGAGATGATTCGACTCTCATTCCAACATATAAACCAAAACGGGAACATCTTACCG
TCAACCCGATTGGATTATACCGTGGCAAGGATAGCCTCCACAGATCCCTTTGCAGCCGTTAAAGCAGCATGCTCAATGTTAAATACAACTGTCGCTGCACTT
GTGTCTTCTACAAGCTGCGAGACGAGCCTAGCTCTCCAGTCGCTGGCGAACTCTTTCGATGTGCCCCACATTATTGTGCCCGGCGAGGAATGCCTCTCGGA
AAAGCATAACAGTTTTACGGTTAACGTGCGACCAAGTCAAGTCTACCTGAGCGAAGCGGTTCTGGACTTGGTATGGTGGCTGAAATGGAAGAGTGTCTCT
ATGTTCTACGATTCAGAGAGCGCCTATAAGAACGTGCAGCAGTTCCTGCATCTGGCGGCGCAATCGGAGGAGCGCAAGCCGCTGGAGGTGACGCTGTAC
CGGGTGGACGATAGGGACCCGGCCAGCGGCGCGCTTGGGATGATTAACATCTTGCACAAAGCTAAGGATTCGGACGTCCAACACATGATCACCTTCTGC
GACAGAGCCCAGAGCATGAGGCTGATTAGACAGGCTGCACGTTTTGGGATGGCCGTGGGGAAATACCAATGGATTATTACAACACAGGACTGGGACATG

ATGGGAGATGGCGAGCCGGGGAGCTTCACGACGGCTGGCGGGGACATCTCTGAGGAAGAGGGCATGAGATTCCTCAATGGCTCCGAGGGCATCATCAC
CTTGATGAAGCAACAGGTCAAAGTTCAGACCCACCCTCTGCAGTTCTACAGCGCCTGGCAGATGCTCTACCCGGACATCGACAGGGAAGAGGACGCTAA
CTCCACTTACTTGTCGCCGGGGTATCTCAACACGATCCAGTTCAAAGCAGCCTATATGTACGACGCCGTACGAGTATTGGCAGTTGCCTTGAACGAACAGG
TTGAGCGAGACAAGTACATTGAACCCGAGATACAGCATTGCTATGACAACAAACCCAAATCATGGAAAGGGGGCATTCGCCTCCGGAAGATGCTGCACA
GGACGGAAACAACTGGGCTGATGGGACGGATGCGGTTTAATCACAGTAGTCTGAATGATGAGATAGCGGTGGATGTCATTACCTTAGAAAGTGGCCGTAA
CCAGACCAAGGCATGGAAGATTGGCGGCTGGGACCCGGAAAATCGTCTAAATTTGGTCAAAACACCCTTTTCAAGAGGTTTTGCTGCTTTTATCAGTAAC
AACACCACATTTCGCATTGTAACCGTTGTGGAGGCGCCGTTCGTTAATCGGGACGAGACAGTGAATGGGTACAAGTACTCGGGGTTTTGCATCGACATGT
TAGAACTAATCGCCAAGGAGTTGAACCTGAAGTATGAGTTGTACCTTGTACCAGATGGAAACTACGGAGGAAAGAACGACGACGGGACGTGGAATGGCC
TGATTGGGGAAGTTTATTATGGTAGGGCGGACCTAGCGGTGGCCGGCATGGTCATCAACTCCGACCGCGAGGAAGTGGTGGACTTCACCAAGCCCTTCA
TGAACTACGGCGTTGGGATTCTCATGCAAAAGCCCAAGAAGAAGGCCAACATCTTCGCCTTCCTGGAGCCGCTCCACATCAAGGTCTGGGGCTGCGTGCT
GGCCTCCCTCTTCGTGGTCGGGGTGCTGATCTACATCGTGGACCGGCTCAGCCCTTACAGCAGTTTCCGGCGGGAGAACAGTCCCAACCCGGAAGCCTTC
GACTTGAAGAACAGCATGTGGTTTGCCTTCGCTTCCTGCATGCAACAGGGCGGCGACACCTCCCCGCTGTCCATTTCTGGTCGTGTCCTGAGCGCGTTCT
GGTGGTTCTTCGCCCTCATCATCACCGCTACGTACACCGCCAACCTGGCCGCCTTCCTGACCGTCACCCGCATGGAGAACCCCATCAACTCTCTAGAGGATT
TGGCCACGCAGAAAACCGTGGTCTACGGCACCATCCTTAACAGCAGCCTGCATGACTTCTTCGAGAAGCGAAAGAATCAGGGTATCTACGAAAAGATGTG
GAACTTCATGTCCACCTCCAAGATCGACCCCTGGGTGCCCAACGCCGAGGCGGGGTACAAGCGCGTCCAGACAGAGGACTACGCCTTCTTTTGGGACGC
GCCGATCCTAGACTACATCAAGCAGGAGGAGTGTGACGTCATGACTGTGGGCAAGCCCTTCAACCTGAAGGGATACGGCATTGCTACGCCGCGGGGGGT
TCCATGGAGAGACGAGATATCAATGGTAATCTTAAAAATGCAAGAGAGGGGCGAACTAGAAGAACTCCGTAAGAAGTGGTTCGACCGGGAATCCAGCTG
CCTGGATGAGACGGACAGCATGAATACGAAACATCGCGCCGCCCGGGCCGACATCAACCTGGACCAGATTGCCGGCGCCTTCTACGTCCTGATCATTGGG
GCCGTGTTGTCCTTTGTGGTGGTCATTGTGGAGCACGTTTGGCACAAGCCGTCCTTCTACAAGAAGCGGGAAAAGGAGACGGGAAGGACCACATTGGA
CTGGTCAGACAAACTCCCTCCACGGGAAACGAGAAACAATGGTCTTTCTAACATTGAAAATTGTTCTTCCAATAAGCATTTAGTTGTTCAAGAGAATGACT
CTTTTGCGTTAACACCTTTGAGACCAAACCCATGGAGCACAACGACAATTCTTTCTGTCCCCCCTGAAGCGACAGAAAACTCTTGCGGATCC

Branchiostoma belcheri XP_019617116.1 (Epsilon, GluE6[18])

Based on XP_019617116.1 from NCBI/GenBank, but replaced the first 42 amino acids of XP_019617116 with the first 27 amino acids, including signal peptide, of rat GluA2 (green below). Codon optimized for *Xenopus laevis*.

GCTAGCACCATGCAGAAGATCATGCACATAAGTGTTCTGCTGTCTCCAGTGCTGTGGGGACTGATATTTGGGGTATCTTCCAACAGCATAAGTCGTTCCGCT
TTATCCATGTCTGAGTTCCCAGCAGCATCCTCCGCTCTTAAGTTGGCCGTTGAATATGTGAATGAAAATGGGCTCGTACCTAATGTTATACTGGAATATATAGA
AAACACTACCACTGCTCTCGCTTTCCTCGACATGATCCAGCATGAATGCTACCAGGCAAAACAAGGAGTCGTTGCTATTATGGGACCCATCCTCTCCTCTCA
GGTGAAGGCGTCTAGCCCTGTATCAACCGGCCTGTCGTTACCGCAAATAGCTCCAGAAGCTACAGATCCAACCCTAGGCAATGCTCAGCAGTATCCACAGC
TTATTCGCATTAGCTGGCCTGATTCTGTGCTTAGTAAAGCACTAATTGATTTGCTCGAGCATTTCAGCTGGGATCAGATGAGTATTTTTGTCAGCAACGATGA
CTATGGTGTGCATGGCCTCGTTGACTTCCAGCTCATTGCTGGAGAAAATGGCTGGAGAATCCACACAATCCAATCCTTTGACCCAACAGAAAATGCAGCCG
ATATAGTTGTACAGTCACAGTTGGGTGTGATCAAAGACACCGGGGCACGGATAATCGTTTTACACTGCCTCGCCTCATATGCAGCAGAAATTTTAAGAGTC
GCCTCTACCATGGACATGACAGGAGCTGGTTGGGCTTGGGTTGTCTCTGATGGAATTACTGGCCTTGATCTTTTTGATACCACAAATGGGACAGCTAATGT
CCCAGACTACCTTCAAGGCCTTTTGGGACCCATTCCTCCCTCTACAAACGGAGCCCACAGCCAGGATTTCATGACTAAATGGAGAGCGGCGGACCCCGCC
GTGTATCCCGGAGCAGGTGTCGGTGATATTGGTCCTTACACCGCAAGATGGGCAGATGCAGTTTTGGCACTTGCACAGGCTCTTAGAAATTTACAGGAAG
ATGGCGTCACCGTCACTCCTCAGCCTCTAGATTGTGGTTGTGATGGATCTGATTCTCAACCATGGGCAGACGGTCCAACTATGCTTCAATACCTGAAACAGG
TCGAGACTGACGGTGTCACTGGAAACATAAGATTTGATAGTACGTCTGCCCGCCTGGATGCTGAATATAACATTGTTAATCTCAAGAGTGACGGCTGGCAA
AAGGTAGGGACCTGGAATTTGACAGCCCGACTGGACATTCATCCAGATGCTGATATACGCTTCTCAGGAGGTGCCACTACAGTAGTCCCTTACATGTCAGA
CCTATCAAACAGAACATTACGTGTGGTGACTATTGCTGCTGACGGCTTTGTAGAGATCTCTGATGTTGATCGGGATGGGAACAACGTCACGGGGAATGACA
GGTTTCAAGGGTTCTGCATGGATTTGTTTTCCTGGATGTCCACTGAGCTGGGATTCAAATATGAGTATTACGAAGTGGAAGATGGACAATATGGTCTGTAC
AATGCCCAGACGGGAAAGTGGAGCGGTCTAGTGGGAGATGTGCTAGATGGCAAAGCAGACATTGCTGTCGCCATAATGAGCATCACAGCCCAGAGACA
GGCCGATGGGGACTTTACACTCCCCTACTATGATAATGGTATTACATTTGCAATGAAGAAAACGGAAGCCAGGACATCTAATACATGGGGCTTTATCAGTCC
ATTCCAGGGTGAACTTTGGGCTACTATCCTGTTGACTGCACTGGCTGTTGGATTGTTTCAGGGGGTGGCCAACTTTGCCACCAAGGATATGGATGAAGAA
AATGATGAACCAGCCAAAGAAGATGATGACAAAGAGACCGGCTTCTGGGAGGCAGTTTGGCAGTCCTTTGTAGCGCTTGTGCAGCTGGGACCTGAGTTC
TTACCGAGGTCTCTGTCGGGACGAATATCAGCATTCTTCTGGGGTATAGGAATCCTGGTGGCAATTAGCACGTACACAGCAAATTTAGCTGCTTTTCTTACT
GTTAGGAATGTAGACAATAGTATCTCATCAGCAGAAGACCTGCTGAGCCAAACAGAGATTTCATACGGTGCAATCAGCACATACTCAAGTTGGGTGAGTTT
TAGAACCACCACAACTGAACCATATCACTCTTTGGGAGTTGCCATGCAAGCCAAGGCGGAGTCTGTGTTGGTCGAAAACCTACAGGAGGGAGTAGAAAA
AATGCGCCAAGAGAAGTATGCTTTGTTCACTGACTCAGCTGAGCTGGATTATATGGCATCCCGGCAGCCGTGTGACTTGCAGACCATTGGCCGGTTGTTTT
GGCAAACAGGATATGGCATGTTTCTGCCCAAAAACAGCAAATACACAGTGGAGTTCAACCATGCCATTGTGAGTGCTGAGGAGCGAGGGGTAGTAGATG
AGCTTGACGTAAAGTGGATTAAATCTCAAGAGTGCTCGGGGACTGATCAAACTGTCCTTGAAAGCTCTGTGATCGGACTTCAAGATATGCTGGGAGTTTTT
GTGCTAGTTTACGGCGGAATGGCTTTAGCGTTAATTGTGCTCATCTGTGAATTTATTCATTCGTGTAATAGAGACGCCAAGAAGAGCGATCCTCATAAACCA
ATGACCGTAACAGAGGCTGTAAAAACACGTCTGAAGAGGATCTTTTCAAGGCGAAAGAACAGTAGTGTTAAAACTCAGCAAGTTCATCCTTCTCACATTC
ACCCTAACACAGCATTTATAGTAGATTTAGAACAGGACTGAGAATTC

Branchiostoma belcheri XP_019616632.1 (Epsilon, GluE8[18])

NCBI/GenBank XP_019616632.1 is incomplete, therefore based on homologous sequence 229810F.t3 from LanceletDB. Codon optimized for *Xenopus laevis*.

GTCGACGCTGCCACCATGGCGGCTGGACTTGTCCTGCTGCTTGTGGCGTCGGCAGCATCTTTGGCTACAGCGGAAACCACCGTCCGTTTTGGTGTCCTGT
CCCTTTCAGACTTCCCTGCTGCTTCTTCCGCAGTCAAGTTGGCTGTGCAGTATGTGAACGAGAAGCAGCTCGTCCCCAACGTCACTCTCAAGTACATCGAG
AACTCAACAGCATCACTGACCTCCCTGGATCTGATCAAAGACGAATGTTACCAGGCATCACAAGGAGTTGTTACAATTATCGGGCCGATGACTTCGTCACA
GGTAAAAGCCAGCAGTCCTGTGTCCTCTGGATTGTCTTTACCCCGTATCGTCCCAGAAGCTACCGATCCTACCCTGGAGAACACCCGACAGTACCGTCAGC
TGGTCCGCATCTCCTGGCCGGACAGTGTGCTCAGCAAGGTTCTCATCGACCTGCTGGAACATTTCAGTTGGGACCAGATGTCCCTTCTGGTCTCTAATGAT
GACTTCGGCGTGCACGGCCTGTTGAAGTTTCAGCTCCTGGCAGGACAGAAACGTTGGCGCATCCACACCATACAGTCTTTCGACCCGACAGAGATTGCTG
TAGACATCGACGTTCGTACGCAGCTCAAAGTTATCAAGAACACAGGAGCCCGTATCATCATCCTTCACTGTCTGGCATCTTACGCGGCTGAAGTCTTACGAC
AGGCTTCCACCATGGACATGACCGGAGAAGGTTGGGCGTGGGTGGTCAGCGACGGGATAACTGGATTTGATCTATACGATAACGACAATGGAAAAGCGG
TCGTTCCAGATTATCTTCGAGGTCTTTTGGGGCCGATTCCTCCTGCTACTAATGGTGCCCACAGTGAGGAGTTCATGGCGAAGTGGAGAGCTGCTGACCCG
GCGGTGTACCCCGGGGCAGGAGGTGCTGACATCGGGCCTTACACGGCGAGATGGGCTGATGCGGTCTTGACTCTTGCACAAGCCTTACGTAACCTCCAG
ACAGATGGAGTCACGGTGACTCCTCAGCCTCTGGATTGTGCTTGTGATGGCGGAGAATCACAGCCATGGGCTGACGGGCCTACAATGCTGCACTATCTAA
AGCAGGTGGAAACAGACGGAGTGACCGGTTATATCCGCTTTGGCCAGACGGCTGCACGTTTGGACGCGGAGTATAACATCGTCAATCTCAAAAGCGATG
GATGGCAAAAGGTCGGTAATTGGAACCTGTCCACCGGACTGAACATCCACTCCGACGCAGATATAAGATTCGCGGGTGGGGCTACTGAAGTCGCTCCTTA
TGTCTCCGACCTGACGAACAAAACCCTGCGAGTTGTAACCATCGCAGCGGACGGCTTCGTGATGATTTCAGATACCGATGAAAATGGGAAGAATGTAACG
GGGAACGACAGATTTCAGGGATTTTGTATGGACTACTTCAAGTGGCTGTCCGACGAACTAGGCTTTAAGTACGAGTACTACGAAGTTGACTCCGTATATTA

CGGCATTTACGATTCGCAAACTGGCCAGTGGAATGGGATGATCGGCGATGTATTGTCTGGGAAAGCTGACATCGCACTGGCTCCTATAGCTATCACCTCCG
AACGTCAGGCAGCCGGAGATTTTACTCTGCCGTTCTACGACAACGGAGTCACCATGGCCATAAAGAAACCAGAAGCAAGGGGTTCCAATACATGGGGCT
TCGTCAGCCCGTTCCAGGCTGATCTGTGGGTCACTATACTGCTGACGGCATTGGCGGTCGGAATCTTTCAGGGCGTCGCCAATCTGGCTATGAAGGATATA
GATGATGAGGGTGATGCACCTACGGATAATGAGACAGGTTTCTGGGAAGCTGTGTGGCAATCGTTCGTGGCTTTAGTCCAGCTGGGTCCGGAGTTCCTGC
CAAGGTCGCTGTCTGGTCGCGTTTCCGCTTTTTTCTGGGGAATCGGAATCCTGGTGGCCATCTCTACATACACTGCCAACCTGGCTGCGTTCCTGACTGTAC
GAAACGTGGATAATTCTATCAGCTCCGCGGAAGACCTTCTTAAACAGACCACCGTTTCCTACGGTGTTATAAGACCCTACGCGACATGGGTGCGCTTTAAG
ACAGCTACAAGTGAGCCTCAGCGGTCACTGGGAATGGTCATGCAGGCACACAAAGACTCCGTTCTGGCAGAAAACCTGGCAGAAGGTCTGGACAAGAT
TAGGCAAGGGAACTATGTGCTGTTTGCGGACAATGCTGAAGTGGACTACCATGCCAGCCGTGAGCCCTGTGACATCCAGACCATCGGGCGGCTGTTTTGG
CAGACAGGATACGGAATCTTTCTGCCAAAAAACTCCAAGTACACCGTGGAATTTAACAAAGCCATTGTCAAGGCAAAGGAACGCTCAGTTAATGATGAAC
TTGACGCTAAGTGGATAAAAAGCCAAGAGTGTACCGGCAAGCAGAAGAGTGTTCTCCAGTCGTCAGTGATCGGACTACAGGACATGTTGGGTGTGTTCG
TGTTGGTGTATGGAGGCATGGCGCTCGGACTCATCCTGATGATTGGTGAGTTCTTCTACACCTGCGCTCAAGATGTGAAGAAGGCTGATGCTCAGCCAAA
GACAATTATGGATGCTGCAAAGATGCGGCTGAAGAAGATGTGCTCCTCCAAGAGAAACAGTAGTGAGAATAACCAGGCTTAGGGATCC

Branchiostoma belcheri GluE XP_019645125.1
Based on XP_019645125.1 from NCBI/GenBank, but replaced the first 34 amino acids with the first 23 amino acids of *Branchiostoma floridae* orthologous (but incomplete) sequence XP_035694464.1 (amino acid sequence identity 86% for the length of XP_035694464.1), resulting in better predicted signal peptide. Codon optimized for *Xenopus laevis*.

GTCGACGCTGCCACCATGGAGGGTACGCGCGCCACAGTATTAGCTTTTCTAATTGTTGCCATGACCTTTACAACAAGCATGGCCGCTGTCGCGCGGTTTGG
GGCCATATTTCCTACTAACAGGTTAAGAGAACTCTCAGCTATGAGACTTGCCGTGGACTATGTGAACGAAAAGGACATCGTTCCGGGGGTCACGTTACAAT
ATCTTTTAAGTACTGCGGAGAGCCTTGACCACTTTGAGATGGTATTAAAAGGCTGCCGACAGACTGAGGAGGGGATCGTAACCATCCTCGGGCCGCATGG
GTCTAGTCAAGTCAAGGCCACTCAACTCGTCAGCTCTAGCCTCAACATACCTCAAATTTCGTATGCGGCGACAGACCCGTACTTAGCAAATCAAGAGATCA
ACGAATACTTACTCCGCATGAGTCCGTCAGACGCCTCCCAGGGCTTAGCACTGGCAGAGCTGATCGAACACTTTGGCTGGAGCCAAATGTCGATCATCATG
AGTACTGACGACTTTGGAACGCACGGGATAGTAGAATTTGAGAAATTGGCGGGAATGAAGGGTTGGCTGATTCGCAGTGTTCAAGGCTTCACCCCCACC
AAGAACGCCGAGGACTTAGACATCAGCCAACAGCTCCGTGCTATAAAGTCATCAGGTGCGAGAATCATCATTCTGAACAGCGTAGTTGAGTACGGTAAAG
TAATCTTAAAACAGGCGAACGATCACGGCATGGTGGGAGCTGGGTGGCAGTGGATCGTAACTGTCGGCATCACCGGATCTATATTATTCCCTGGCGTTGAG
GACATGCCTGATTATTATGAGGGGCTACTCGGCGTATACATAGTAGATGATGCGGGGGAGTTGAAAGGGGAATTTTTACGCAGGTGGATGGCAGCCGACC
CTGTGTTGTACCCTGGGGTTGGGAACGGGACGATGGTGGCGTACGCAGGAAAGGCCATGGACGCCGTGTTACTGCTCGCGTATGGGTTACGGGACATGC
TCGCTGCGGGGGAGACGGTGGACACCGAGAGCCTCAACTGTTCCCTCATCCCGCATCCGGAGTGGTCCAGGGGGGAGACTTTCTTACAGTACCTGAAAC
AGGTAGATGGCCCCGGTGTGACCGGGCGTGTAAGCATGAAGAGCGACGGCAGCTCAAGGAACGCGGTATATGATGTAGTAACTCTCGATAAACACCGTT
GGAAAAAGGTCGGACGGTGGAACGACAGCTCGGGACTAACCTTACCCGAAAGCGTGACTTTCATGGGAGGACAGCAAGAGGTAATAGACTTCATCAGC
GACCTCAGCAACAGAACTTTAAGGGTGGTGACTCACAAGGAGCAAGGGTTTGTCCACATCAGGGACACGGACGACTGGGGGAAAAACCTAACCGGCA
AAGCGAGGTTTAGCGGGTTCTGTATAGACCTCTTAGAGTGGTTAAGTGAAAAACTTGGCTTTAAATACGAGCTGTATGAAGTAGAAGATGGGAACTTCGG
GGCGTACGATCCTGAGCTGAAACGTTGGAATGGCATGGTTCAGGACGTTATTACAAAAACAGCGGATATAGCGATGGCCACTCTGACGATCACGGCCCAG
CGGGAAGAGGCCTGTGACTTTACCATGCCGTATGTAGATGTCGGCTTAACCTTCATAATGGGCAAAGAGGAGACTAAAACCTACAGCTTGTTAAACTTCTT
AACACCGTTCGAGAGGGATCTGTGGTTCATGTTTGTAGTGACGGCGGTAGCCGTGGGCATATTTCAAGCGATAATCAACTATTTGAGTCCATTCGGCTACC
GGCGACAGGAACAGCCCGCGCAGGGGGAAAGGAGTAAACGCAGGAGGCGGCATGAGGACATCAACGCAAAGCCGCCGTACCGCCTGTCTGACGCCG
TGTGGCAGAGTTTCACGATGCTGGTACAGATCGGACCGGAGATCTTCCCCCGGTCTTATGCAGGACGTATCACCGCGATTTTTTTCGGCCTGGGCACTATG
GTGCTGTTAGCGACCTACACTGCCAACTTAGCCGCCTTCTTAACAGTAAGCAGGTTACAAAGTGGCATCAACAGTGTAGACGACTTAGCAGGGCAGAGCG
AAGTATTATACGGAGCGCGAGGCAGCGGGGCAACTGAGAGTTTCTTTTTGGACTCGAAAATCGAGCCTTATATAACCATGGCCAAACTGATGACGGAGAA
GAGAGACGACGTCATGGTCAAGAGTATGGATGAGGGGTTGAGGAAAGTGCGGGGGGGGGAAATATGCCTTCATTAGCGACAATGTTGTACTGGACTACGT
AGCCACTAGGCAACCGTGCGACATCAAAACAGTGGGCCGTCTGTTCCGGAATGCTGGCTACGGCGTACTATTAGCCAAAGGTTCGCCTTACACAGGGGA
GTTCTCTAACGCCATAATTACAGCCAGGGAGAGCGGCTTTATAGAAACTCTAACGAAAAAATGGATTACAAGTAACGAGTGTGACAAGTCCCAGAACACT
GGCGCGTCTCAGGACCGCATAAGCATTAACAAAATGTTAGGCGTATTCGTTGTAATATATGGTGGTATGTGTGTCAGCCTGGTGGTGCTGGTATGTGAGTG
GCTAGTGGCGTCATGTCGGGACGTCAATAGAAATCACCCCAAGGCACCGAAGTCGATAACTGAAGCGATTGCCAGAAACTTTAGGTCCCAGGCGAGGAG
GTATAGGTCCGACTGGGAAGCGTGTTGTGGCAACCAAAGAGAGAACGAAAATAGGAATGAGACGCACAGGAAGAGGAGGTCGGCAAACAACAACACG
TCTGCGAATCACCACAACACTTTTGATCACAGGCCCTTTAGCGTGTGTTCGAGCGAACCAGAGGGAGAGGAGGAGTTATTGCATGTAACCGTATTGTAAG
GATCC

Nematostella vectensis 5513056 (AKDF)
Refers to nve:5513056 from KEGG database, but based on transcript NvERTx.4.76412,[6] which has a better predicted signal peptide (93.8% amino acid sequence identity between the two sequences).

GCTAGCGCTGCCACCATGGGTATTATACAAGTATGTCTGTACTTTACCTTTATTTTCTGGAATGGCTTCAGTTCAGGAGAAGATGTTCAACAAAATATTGCAG
GTGTCGTCTATCACAAAACAGATTCCGTGCTGTTCAAAGCTTTCAAAGCGGGTATTAAGCGACTTAACGAAGACAAGATGTTACCCCACAACAAGACACTT
GAAATAGCCCAATTCAAAACCGATGGGAGGAATTTGCTCGGATGCGATGCGGTCAAGAAACTACGAGAGGCTTCTTGCATCTTTGACCTGTCATTCGGCG
ATGAGCGATCGTTTGCTTTAGCGGAGCTTCTCCGAGTGCCAATGGTCGTGCTGAGACCCATATATGGAAGTCGGTGGAAAAGTGAGTTCGTGTTCACGAT
GCAGCCGCCGGAGGATTCCACTGCTATGGCTACGGTTAATCTGCTACATTACCTGACCAAAGAATCCAATGTCAAGAAGGTCGCTATACTGGCTGAGAGGA
AAAGATCTCACTTGGGAGCACGCATCTTCTCGCTCCTGCGTAGACGAGAGCGTGTCATGCCTTACATGATGCCAGAGCTTGTGATAGGCGACGACTCTCAG
AACACCAAGGCTCTCACCGAGCTGCTCAGGTCTGGGTTAAGCTACGTCATACTCGTATGTCCTCCAAAGGATATGAGGGCTCTCATGGAAAAGGCAATGA
GGCTAGGGTGTGTCAATAAAGAATCAAAATGGATCACAGCAAATATGGAAATCCCAGGGAACATTTCGGCCCTCGCTCTTCGCGGTCTCCTTGGCATCCGT
TTACACGCGACTGATACTGGGAGACTGAGGCGAGAGGTCGGCAACAACAGGGGCTACCCAGAAATCACTGGGCTGACCTATGCCGTGCTTAACGACTCC
ATGTACGTTGTTGGGCTGGCTATAAGAAAGATGATGCTGAGTCCGATTATTGTGGACGAGACAAAAAGTAACAAATACTGCCCTAAGAATTTGGTCACTAC
GCCAGGGAAGAGACACAACATACTCAACTTCTTGAGTAAGGTCAACTATCAAGGTTTCACTGGACCGGTGGTTTTCAAGCAAGGATCAAACGAAAGAGT
CGTCCAAATATTTGACATTTTGAATCTGCAACTTCAGGAAGACGAGCAAACGACTAGAAAAATCAGGATTGGACACTTAGTAAATAACGAAACTAGAGCC
AGCAAGATCACGATGGACACAGAAGAGAAAATATTTTGGAACGGAGAATACTGGGGTGATATTCAATGCGAGTTCCGCGGTAAAGAGACCATCAAAGCG
CCTCCAGTCCAGCACAAAGTTCTTATCGTCACCGTGAGAGAGGACGAACCGTTCATGATGATCAAACCCAACGCGTCGCTTCTTAAAGGTAACGACCGTTA
CCGTGGCTTTAGTAAGGACCTGATGGAGCTGCTCTCAAGAAAGCTCAATTTCCGGTACGAGATTTGCATCAGCAAGGAGAGTTCATATGGCTCCAAGGTA
GAGGGCCAGTGGCAGGGGCTTATTGGAGAGCTAGTCCGAGAGGAAGCGGACATTGCACTTGGTCCAATTACCATAACAGCCGAGCGGGAGGAGGTGAT
CGACTTCAGTAAGCCTTTCCTTGATTTTCGCATCGCCATGATCCTACAGCAGCCTACTGGCGAAGAAGTCAACCTGTTCGCTTTTCTTCTTCCTTTCGACGA
AAAGCTTTGGCTCACCACATTAGGAGTGGTCGGTTTAGTGTCTCTTATTGTCTGGTTTTTGGATCGGTTCAGCCCTCAAGGTTACAAGACCCAGGCAGAGA
AGTCGGGTGAGGGTGAAGGCGACGAGTTCAGCTTGTCCAACAGTCTCTGGTTCGCTGTTGCCTCAATTCTTCAGCAGGGTGGGGATAATACACCTCGCTC
GACTTCTGGAAGGGTGTTGGCGGCGGCATTCTGGCTCTTCACGCTCATCTTAATCTCCACCTACACTGCAAACTTGGCGGCGTATTTCACAGCCAAGAGCA
TTCGATCAAAGATCAACTCTCTGGAGGATCTTGTCAGTCAAAGCACAGTAAAGTACGGCGTCCAGGAAGGGGGGTCACTCTTCACATTCTTTAGCGAGTC

CAAGGTGGACATCTACGCCACAATGTTCCAGCAGATGAAATCCCAGGAGACATTTGTAAATGGCACACAGTACGGCGTCCTTAAGGCTCGTACGCAACCG
TTTGCTTACTTGACAGACCAACCCTCACTCGATTACTACAACATGCGTAAGCCCTGCAACACGATGTTAGTGAAAAACCTGCTTGACGCCAAGAGTTATGC
GCTGGCGATGAGGAGAAACTCGGAGTGGACAAACTTGATTTCTGTCGCCATCTTAGAGCTGCGTGAGGAGGGTGAGATCGAGAAGAGTCGTCTGCGCT
GGTGGGATGACAGCAGCGAGTGTCCGACGGGGCCATCAACGGACTCATCGCCGCAAAGACTGGAGCTGCACAGCTTGGCCGGAGTGTTTATCATCCTAG
GCGGCGGCGCGACCATCTCACTGGTGTTACTGCTGGTGGAGAATAAATGCAGAAACGTTCTCGAACCGGGCAGGCCAGAGTCTCCTGAGGGCGACGAG
ACGGACGCTCTGACACGACCCGCTCCGGCGATCACAGCAAAAGGCGGCACAGAGCTGAAGCTAATAAATAACTCCGAGCGACGCCAGGAACCGGACGG
GAAGAACAGGAGGGCATCACCCAAGCCGGACAGCATACAAAACCGTGCCCGCGCGCAGCCTGGGTACGACCTCAGAATCCCAACCGTCTACGCATCCGC
CCCAGAAAGTAATCTCTAGGAATTC

Nematostella vectensis 5503753 (AKDF)

Based on nve:5503753 from KEGG database.

GCTAGCGCTGCCACCATGACAATAAGAGCATCATGGGTAATCCGGCAGACCGTGATGCATCAGGGGCCGTGTGTCTGTGTCATGTTGATGTTGTTGTTCCA
CCTCCCAGAGTCTGCAAGCGTCCCCAGTGTAGTATACATAGCCCAGCTAGGGATGTCACCATCCCAGCAACCACTCAATGCAGTAGAATCCGCCATTCGCTA
TGTCAACAAGCGATCAGATCTTTTGCCAAGTACGACAATCAAGCTTCTCCAGGAGACGTATTCAAAAGATAATCTATTACAATATGCTCTCAACCTCTCCTCC
TATGACGTCATCGCGCTGTTGGTTGATGCTCACCCGCAGCCTTCCCAGCAGGCCATGAGCGCAATGTTTCGTATCCCCATGGTAACCCTACATGAAGCACAC
ACGTACACCTCTCAGACCCAATCTTTGCTGCAATATGGAATATGCGTCGGTCCTAGCGGATATGACGTCACAAGAGCGATGAATGACGTCATAACACACCTG
AATCGTAACAAAATCCTCGTTTTATATGACGGACGCTGGCGCAGTCAAGCGATGCTTTTTATTTCGCGGCTTCCGTCAAACTCGAGTTACTCCAAGATGTCC
CTGGAGATCGAGTCTGACTCGTGGTTGTGCGGAGACACCAAGGAGCTGCTTGGTAGACTCAACAATGCACCGACAGAAGTCATCGCGCTCTTCTGTGACT
CGTACCTTGTGGCGGAAGTCATTGACAAGGTGCACTGCGCATGCTCATTCAAAGTGGAGAAACCGCATATCTGGCTGATCGCAGATATGATCCCTTCAGTA
TCGATGCCAGTGCAGTCGTCTCAAGTCGTACTTGGTTTTTTGCCTCAAGTTACAGACAGCTACCTAACACGACACATCGACCGGGAACTGAACCTCAGCAA
AACCGCGATATATAATTCGGTGTACTTCGCGTACGACGCCGTTTTAGCCACCGCGCATGCTCTGGATAAAGTCATTCTGGAAGACCGCTGGGTGTCATTAAA
TCGGTCTCACGGGCTCACAACTTCTCTAAATGGGCCTTCAATATTTGAAGAAATGAAGAAGTTGAAGATCAACTCAACAACAGGAATCCTGGACATCGCTC
ACGACGGCAGTAGACGAAATTTAGAGCTGGAGGTCTTTAACCTCCAAAACGACATTTTTACTAAGATTGGCGTTTGGAGCGCCAGCAGTGGTGTGACGG
TCAGCGGCTCTGTGAAGCAGAACAGGCCCACCACCGACCTAGTCCCTCTCGATAAGCTCCACCGGAAGTTGGAGGTGGTCGTAGTACTGGATTCCCCTTT
TGTGATGCTGAACGAGGCGGGTGTACTCGAAGGTTTCAGCATTGACCTAATCAAAGAGATTTCACAGATGCTCGGCTTTGCCTATAACGTGTATTTAAGTC
CTGATGGCCTATACGGAGGGAGCGATGGCCTGGGAGGCTACACGGGAATCGTAGGGCAGATCATAAAAGGGAAAGCGGATTTATCGACATCTCCGCTGA
CCATTACTTCGGAGCGACTTCAGTATCTCGAGTTCAGCAAGCCCTTTATGCAGTTCACAATGAGCTTGATTTCCAAGAAATTCGACAATGATAATCAGTATCT
GATGGCCTTCATGCGTCCCTATTCTTCTACTGTCTGGCTTCTGACGCTGGCAGGTCTGCTTCTGGTAACGATTCTCATGTTTGTCGTGAACTACATCAGTCCG
TATGGCTACCGTAAGTCGCATAAGAGCAAGAATGGAGAGGCGTTTAACTTTTTCAACAGCTTGTGGTTCTGCCTTGCCTCCATGCTACAACAGGGCGCGG
ACAGCACCCCCCGGTCACTATCCGGTCGTGTCCTAGCGGGATGCTTCTGGTTCTGTATTCTAATCTGGATCTCGACATACACTGCCAATCTGGCGGCGTTTTT
CACGGTCCGCAGCGCGGGGAAAACTGTGAGCACTTTGGAAGATGTTGTTGATGGTGGCTATCACTTCTACATCATACGTCACACTGCACTCTACCACTTCT
TTAAAATGGCTTCTTATCATACCTACAGAAAGTTATGGGAGCGTATGAGTGCAGAGAAAACGTTTGTGAACTCGACGAACGAGGGCTACGAGGTGGCACG
GAAGAGGAAGAATGCGGTGTTTATGGCAGAGAAGCCGTCCACCGAATACACCATCATGCGGGAACCCTGTGATCTTCAGGCAGCAAGCGGCCTTATGGG
CGCCAGTAGCTACGGCATGGCGATGCCCAAGCACTACCCTTACATTGGGAACATTTCTGTGGCTATTCTACGGCTCCATGAAACTGGTGTCTTGGACTCCCT
GTGGCGCAAGTGGTGGGACTACCGTAGCCAGTGCCCCAAGGAGCAGACTCGGTCGGCTATCGGTACGGGCAAGCAAATCGACCTGGACAGCATGGTGG
GCGTGTACGTTGTCTTGGCAACAGGCGGCATTGCCTCCGTCATTCTGGTGATGTGTGAGATCTGGTGGAGTCGCCATGGCAACAGGGTAAAAGCGCACTT
CAAAAGCAAGTTTGGCACAGGAGGAACGAAGAAAAATCATGCCAATGACGTCACTCGGATGTCAAGAATAGAGCAGTATAAACTGGTATCACCCAGCCCT
ACCGGTAACGATCAGTTCACTACCGAGATGGAAAAGAGGTGGCCTTCTGATAGCACCAACAATAGCAAAGTTCCAGTAGTGCGAGTCACGTCTCATTCAA
GAACCACCGATCTGTAGGAATTC

Exaiptasia diaphana 110238381 (AKDF)

Based on epa:110238381 from KEGG database.

AAGCTTACCATGGCGTTGGGAGTGTGTTTTATTCCAGTGGTGTGGTTTTCCATTTTATTGACTTGTGTTCAGTCTTCACTCAAGTCCAAAGGAGATTGGCAG
CCCATTATAGGTGTTCTTTACCATCCCCACGACGAGCCTTTGTATGAATCGCTCAGGCAAACCATCAATCGTATAAACAAACAAAATACATTACCTGTAGCAA
GGCTAACCACGATCAGCTTTAAGACCCACAGTGACAAGATCCTTGGTTATGATGCAGCCAAGAAGCTCAAGAAAAGCACGTTTATTTTCGACCTGTCGTTC
AACAACCAGTACTCGTATGCACTGGCAGAATTGCTACAAACACCTATGATAACACTGGAAACCATGCATGATAACATAAGGAATTACTTTATCTTTTCTTTGC
GACCATCAGAGGAAACGGTGGCCAGAGCAACTGTAGATCTAGTACAGTACTACATGAAGTTCAATGCTCAAGTCGCAATTTTGGTGGACGCCAAGATGTC
TCACCTTGGAGCACGTATCTTCTCTATTGCTCGTTATCGTAATCGCATTAGACCAATCATGATGCCAGAAGCCAACACAAAGAATGAAATAGAAACACAACG
AGCTTTGTCAGTTTTACTTCGAACCAACAGCAGGAATGTTATACTGGTATGCAAGGCAGAAGACACTCGGCAATTCATTGAAAGGGCTATGAGAAATGGT
TGCGTGTTTTCGGACTCAAAGTGGTTTGTCGTTGATTTGGAATTAACGGGCAATATCTCCGCGTTAGCTTATCGCGGTCTGTTAGGACTTCGTCTTTACTATA
ACAACCAACACAAACTCAAACGTGATGCAGAGAAAAGTAGAAATGGACGCGCAAAGAAGATATCAATGGCGTCCTATGCAGTACTAAACGACTCTCTGTA
TGCAGTCAGTGAAGCTATAAGACAAACAAAACAAATCACTTTCGATAACAGAGGGAAAGACGACTGTCCCATCAAAGGTGCCATGGACGATAGAGGGCA
AAGTAACTTCATTAACAGTCTTATGGGGGTTCGATTTGAGGGTCTCACTGGACCAGTTGAGTTCGACCAAAAGACGCGAACTCGTAACGTTAAAATCATCG
ATGCTGTTAACGTACAAATCAAAGCTGGTTTTCCGCCTTCGTTCATGTATCCGATTGGTTATTGGAAGCCGAACGTTGACTCACCGATTGGATCTGTCGTACT
GAAGACTAACGATGCTGTGTTCTGGAATGGTGATTACTGGGAGGAAATCAAGTGCAGTTTTACGTCCAATTCTCCGTCATTTATGACCAATGACTTTGTACG
GCACCACAAACCTCTAATTGTTACATCCATTGTGGATTCTCCATACATGATGCTGAAGAAAAACCATTCAGGTCTCACAGGAAACGACAGATATGAGGGTTT
TGGTGTTGACATGATTTCAAAGCTGGCTGAAAAACTAAAATTTAAGTATGAAATCAAAATCAGCACTGAAACTTCTTACGGCGCTCCGATTGGTGGAGAAT
GGAAGGGTTTGGTTGGAGAACTTGTAGATCAGAAAGCTGATATAGCACTGGGACCTATTACCATCACAGCAGAGAGAGAAGAAGTTATTGACTTTAGTAA
ACCTTTTCTTGATTTCCGCATTGCGATGATTCTAACCAAACCTGAAGGAGAAAACGTCAACTTGTTTGCTTTCTTGTTGCCATTCGAGGAACAGCTCTGGTT
ATGTGTATGCGCTGTGGTTGGTACGGTGACCATTATAATGTACGTTCTGGATCGTTTTAGTCCGTATGGATATAGAGCCGAGGCTAAAGATACCGGAGAAGG
AGATGGAAATGAGTTCAGCATGTCAAATAGTCTTTGGTTCGCGACAGGATCTATTTTACAACAAGGTGCTGATACTACTCCTAAGGCTGGCTCAGGACGTG
TCCTGGCTGCCGCGTTCTGGCTATTTACATTGATATTGATTTCAACTTATACAGCAAACCTGGCTGCATATTTTACAGCTAAAAACGCGAAATCTACGATTAAC
TCACTCGAGGATCTGGTTAGCCAAGACAAAGTGAAATATGGCGTTCTTAACGGAGGATCTTTGTACACATTCTTTGAGAACTCAAAAGTCAGAACCTACAA
AAAGATGTTTGCCAGGATGAAAACTTTGAATACATTCGTACCCAGTACAAAAGTCGGTGTGAACATTTCCAGACAGGGTAACTTTGCTTACCTCACAGATC
AGCCATCATTGGATTACTATAACCAGAGAAAGCCTTGTAATACAATGCTGGTCAAGAATCTACTGGACGCCAAGAGTTACGCTATAGGCCTACAGAGGAAC
TCTGAATGGACAAACAAAATATCAGTCACTATTCTTGAGTTACGTGAGGAAGGAGAGATTGAAAAGATACGATTGCGATGGTGGGACGATCGAAGTGAAT
GTCCTGCATCTGATGCACCAAACACTGCACCATCACGATTGGCTCTTCACCATCTTGCAGGCGTGTTTATCATCCTTGGTGGTGGTGCAGCGATTTCGTTAG
CTTTACTTCTTGTGGAAAATAGATGTCGAGATGTATTGAGTGGCAGAAGACAAACACAGACTCCAGAAACTCCTCGGGATGACCCTGATGGCAGAACCCG
TAACCCGCCAACAATCACACTTAATGGCGGGCCGACCCTCAAACCTCTAAACAACACCGAGCGAAGATCCAGGCAGGATAGTCCATCCAATAGACGAACG
AGCTATGATGTCCGTATCCCGGATGTGTATGCAGCCGCGCCCGAAAGCAACCTCGGATCCGAGCAGAAGCTCATCAGTGAGGAAGATCTCTAA

Nematostella vectensis 5511254 (Epsilon)

Refers to nve:5511254 from KEGG, but based on transcript NvERTx.4.104052,[6] identical to nve:5511254 but eight amino acids shorter at the end of the C-terminal domain.

GCTAGCGCTGCCACCATGTATCTGCCCTTCTTGGCACTCTTTTTCTGTTTTTCGATCGCTTCTGTTAAAGCCCAAGGCAAAAGAATACTTAAAATAGGTGGC
ATTTTCGAGAAAGAAAATGACATTGATGACAGGGTCTTCCGCTTCGCCGTGGACTGGGTGAACGCTAATAATATAACTAAAGATGTTGTATTTGAGCCCGT
TGTGTCTTACGCTAGTCAATATAGCTCGTACAAAAACATCGAAAGTGCAAGCAAGCTGATTTCGCAGGGCGTCTTGGCCACCGTGGGGCCTATGACCTCGC
CTGATGTTAAAGTCACTCTCGGTCTGTTTAAACGCTTCAAGATTCCTCTCTTAGCTCCTAGCGCAACGGATACTGCCTTCATCTACGACAACTACCGTTACAA
ATTCCTACTACGGATGCTCACACATAACGCCGCGCAGGACATTGCGCTTGTAGACCTGATCAACCAGTTCAAGTGGGAGAGAATGGGAATACTGACGTCA
CGCACTGATTACGGCATGAATGGGTTAACGGACTTCAAGGAGTACGGCGTAGGTCAGTTCAAATGGGTTATACTCGCTACCGAGCACTTCGCGGTGCCCA
CGGACTACACCAAAATCAACGCCACCAAGCAGCTTTTGCGTCTACGTGAGAAGGGAACCAGAGTCGTGATCTTAAATTGCATGTCAAAGTACGTCGAGTA
CATCATGAAACAAGCAGAAAGACTTGACATGCTCAATGAGTGGGTCTGGATACTTACCGACGGAGCAATTGCCAAGGAAGGAAGCACGAAATACAAAGA
GGGTCTTATAGGGGTTCGATACCCACAGACTGGCAAGGGGCGACTCTCTGGACCTGTGAATAAGGCATGGAGTGCAAGGAAGGAAAAGACTAAAATTG
AGGGACGAACTGGGCGCATTTTCGATGCAGTGCTAACTATAGCGTATGGTGTAAATCTCCTACGTGATAATGTCACCATCACGGACCCACCCAAAGGAGAC
AGTTTCTGTTCATCCAGCAACTTAACCGCTCAGCCTTGGTCCCTTGGTGAACAGTTTTACAATTATCTAAAAGAGGTCAAGGGAGATGGTTTTATGCACCA
GATCGCCTATTCACCCATGGGACAACCACGTGCGCGTTCATTTGACATCGTTAACTACCAGACGGAAGATGGATGGGTCAAGGTTGGTTACTCCATTGGAT
TGAGGGTGAAACTGGAGAAGAAAAGACCAGTGATATGGCTCAACGGTGGGATTGAAGTACCCAAACTCGCCAGCCTTGCGCTCAAATACAAGAAGCTCA
AAGTGCTAATATTCGAGAGTCGTCCGTTTGTAATGGCAAAGACAGGAAACATCTCTACATCGGATAACAAGGTCCTGTATGAAGGGTTTTGCATCGATTTGC
TGGAGGAATTGAAAAAAAAGCTGAGTTTTGATTACGAGCTGAAATTGATGAAGTCATTCGGCGAAAAGCAACCTGACGGCTCTTGGGACGGTATCCTTG
GCGACCTGACTAGAGGCAAAGCAGACCTTGCAGTGTCCACGTTTACGATCAGCCCCGAGCGCGAGAAAGCGGTGGATTTTACTCAGCCCTACTTCAGCCT
CGGCTACAAGATCGTGATGAAGAAGGCGATCAAGGAGAAGAAATCCAATCTGTGGGGCTTCCTTGATCCCTTTGAACTGACGCTCTGGGCCGCTATCGTA
GTGTCATCGGTCGTTATAGGGACGGTGGTGTGGATCTACGATCGCTTGAGTCCCTATGGCTACTATGGTCGCGCTGTGCAGTCTAAAGTTCCCACGGAGGA
AGAGTATAACACAAGGAACACGCTGTGCTTTTTTAACTCCTTCTGGTCAGCTACCGCCTCCTATCTCGAGCAGGGTCCGGACGGTACTCACCCCATCTCGC
ATTCCGGGAGAGCTACGATACTCGCATGGTGGTTTGCAATATCTATATTTGGAGCCACCTACACTGCCAACCTAGCGGCCTTTTTAACTGTTAACAAATACG
AACATCCAATCAAAAGCATTGAAGATCTCGCTGATCAACAAGGCATAGCGTATGGCACTGCCCCAGGCCAGCTGGCGGAGATGTTGAAGACGGCTTCTTT
ACCCGTCTACGAGAAGCTCTGGAGATATATCGAGACACACGGGACCTTAGAAGACAACGCTACTTACGCCATCGACAAAGTTCGCTCTAGGGATGACTATG
CATTCATCTGGGATTCAGCGGTGTTGGACTACGAGGTTCAAAGCGAGCCGTGTAACACGCTCACTATAGTAGGACGGCCGTTCGGCAGTATAAATTATGGT
TTTGCGTTGCCGCTCAAATCTCCATACACGCAGAACTTCAGCGTGGCCATGCTCGAGCTACAGCAAGATGGCTTTCTTGAGCGCATGACAGAGAAGTGGT
TCCGCTCTCGCAGTAAGTGCGGCGCGGAGACTGAGGCTGCGGACGCCAAGGCAACCGAGGGAGGGGAGCAGCTAGGGTTCAAGGACATGGCGGGCG
TGTTCATCACACTACTGGGAGGATTCATCTGCGGAGTGGGGCTACTGATTGTCGAGTGGATCTTTGCGTCTTATAAGGACACTCTCTCTAAGGACAGGAGG
GCACCAACAGGTTTCTTCACTGCCTTGTGGTTACGTCTTCAGAGAATGTGGTGGGGCATACTAAGCTTGTGCAGTTCCAGCGAGCTGGGACGCGAGGAA
GAGCCTAAGCCGAAACTACGGGCGCTGGTTGAGGAAATACCTCTAAGTCATAGAGACCGAAAGAGCTTGCTCGACAAGAGGTGGTCACAAATACGCGAA
TAAGAATTC

Nematostella vectensis 5500937 (Epsilon)

Based on nve:5500937 from KEGG database.

GCTAGCGCTGCCACCATGCGTGGATGGCGCGCAGCTTTGCGCGTGGCACTTATTGTATCGTTAGTAGAGTGCGCATGCGTGAACTCTACCCAATCAGCGCC
GACGGAAATCAAAATTGGCGGGATATTTTGCCAGGAGAACAACAACCACGAGTACAAAGCCTTCAATCTGGCTATTGAGTACATAAACAACGACACCAGC
ATCCTTCCTGGTACAACCCTGGTACCAATGACCAACAGCACAAAGTGGCTTAACGCCTTTAACAACATCGAGGCCGTGACTTGGCAGATCTTCCATCAGGC
TGTGGCCATCGTCGGACCTCTCACCTCCCCCATGGTGCGCGCGACGCAGCCGTTATGCAGCGGGTTTCATGTACCACAGGTAGCCCCTTACGCGACAGACC
CCGCCTTTGAGTTCTCGCCAAGCTCCTACAAGTACTTACTGCGCATGCGTAGCAGTGATTCCATAGAGAACCGGGCGATTGCGGATTTTATTGGACATTTTA
ACTGGACGCGTCTAGGACTCTTCACATCACGTGACGATTATGGGCTGAATGGCGTCGCTGCCATTAAGGACATCGCCTCTCGCATGGGATGGGTGATAGCG
GCGGTGGACAGCTTTCGGCAGTTTGAGGACCCCTTGAGGGTAAATGCCACCCAGCAGCTTGTGCAACTACGAGCTAGAGGAATACGGATAATCATACTAA
ACTGCCTGGCCTCGTATGCCCGCGTCATCCTCAAGCAAGCGAGTGAACTTAACATGATTAAAGATTACGTGTGGATCGTCAAAAACGGAGCATTTTCGTTC
AAAGGCTTGTTTGACTCTGAAGATAATGTCCCGGACTATATGCAAGGGGTGGTTGGTATGCGGACCTCTTTTCGTGGTGGTGTGTTACAGGATGAAGTTAA
GAGGGCCTGGGTGAGTGCCGGTTATGGAGAGATGGCGATCGAGAACGAAGACGCAGTCGGGCATACTTTTGATGCGGTTTTAGTGTTGGCGCATGCGCT
CCATAACATGTTAAACGATGGACACAATATCTCGAACGTCCAACCCCAATTCGGCTTTTACGACGGCCTCTCGACAGAACCCCGACCAGATGGCGCCACAC
TTCTCGACTACATTTCCCAAGTCAATACCACTGGAGTGATGAATCAATTAGGCTTCGATAGCAATCGATCACCCGTGGACGTCGCTTTCGACGTTGTCAATTT
GCGGGCGTTTGGCTTTCAAAAGGTCGGCTATTGGAATGTAGAGGAGGGGCTTCACCTTGATAACAAAAAGGAGATCGTGTGGCCGTCTGGGAGGGTGT
ATGTTCCGACAGACAGCACCCACATATTAGAAAACAGAACGCTGAAAGTTGTTACCATCGCAGAAGCTCCGTTTATATTTGCGCAAACGCAAACAAACGG
CCAGGGTGAAACCAGAGTCATCATAGAGGGATACTGCATAGAACTTTTGCGTAAGTTGTCCGAAATGCTACGCTTTAAGTTTGAAGTCTACCTCGTTCCTG
ACAACAACTTTGGCGCTCAAGATCCAGTAACAAAAGAGTGGAATGGAGTGGTAAGGGAAGTTCTGAACGGACGTGCGGATCTAGCTGTGACCTCCCTAA
CGATCAGCCCTGAAAGACAAAAGGTGATCGACTTCACACAGCCCTACATGGATCTGGGTCTTACAGTCCTAATCAAACCTGATCCAACAGAGGAAAAAAA
CCCCTTTGCTATCCTACGCCCATTCCGTTATGACCTCTGGATGGCTATAGGAGGAACTATGATCATCGTCGGGTTCTTTCTATGGCTGTTCAGTACTTTTAGCC
CGTTTGGATTTTACGGCCGATGCGTTCAGAAGTGTCACACGAAGATTGAACCGAGGTATCTGAAACTACATGACACGCTCTCTCTTGTCCGGGCACTATGG
TCCACGGTGGTCTACTACGTCGGACAGAGCTCTGATCATTTGCATCCGGTCAGTTCCTCGGGTCGTATCACTGTGGCGGTTTACTGGTTTGCGATGCTCATT
GTAATGTCTACATACACAGCTAACCTCGCGGCGTTTTTGACAATCAAACGATTCACTTCACCAATATCGAGCGTCGATGATCTTGCAAGACAAAAGGATATT
TCGTATGGCACGGTGTTAAATAGTCAGCCTCAAGCGTTTTTCGAGTCAGCGTCCGTACCCAGCTTTGTCACGATGTGGCAGTACATGCGCTATCACCACACG
TTCGTGAATAACAGCGCAGAGGGAATCGAGAAAGTCATGAACGAGAACTACGCGTTTATATGGGACTCCGCGGTGCTTGAATTTGTAGCACATAACCAGA
TTTCATGCGGGACTCTGATCACTTCTGGAAGCGTCTTTGGTAGAATCGGGTACGGCTTTGGGCTGGCCAAGGACTCACCATACACCAAGCAACTAAGTAA
CGCAATCCTACAGCTCCGTCACGCTGGCTACATGGAGTTCCTTGACCGGAAGTGGCTGAAAGCAAATGACAAGTGCGCAGAGGCTGCTGAGAAGGCGA
AGAGTGAGAACCAGCTGACTTTCGAGGATTTGTCGGGTGTCTTTATTGTCCTTATTGCAGGTATTGGTGTCAGCTGTGTGGTTCTAGTACTTGAATGGATAT
GGGCTTCGTGGCAAGACACTAAAGAGAGCCTTAAGACAGAGGGGACTCGACGTTCCTTGAAAGCGGCCCTCAAGGAGAGGGGCCATCGGGCCTTCCA
CGACTGGCACCACCGGGATGATGTCCCTCAGGTGCACCGGCACGCCAAACATTGCTGGACAAACGCGAAGTTCTGTCTGGAAGACCAAGTTCTCCAATTA
CTTCAGTCAGTGAAGCGAAAAGTCTGCGGTGACGTAGCACCGTCTGTATCCGATATAGCGCTCGGTGAGACCACCATTGTCGAGGATTTGGAAAGAGTCA
TAGAAAACGAGAGTAGTGATGACAGCCATTACGAGGTGTGAGAATTC

Euplokamis dunlapae AQX17743.1 (Epsilon)

Based on AQX17743.1 from NCBI/GenBank. Codon optimized for *Xenopus laevis*.

GTCGACACCATGGTGTGTATGAACTCCCAGAAGGGCTGCTCTCTGGTGGTTATCCTGCTGCTGCTGTTCGCTAACGCTGAGCCTCCTGAGGAGATCAGGAT
CGGGTTGCTGAGCCACGAGGGACTGCAGAACGTGGAGGCCGCCTTCGACTACGCCATCAAGCTGATAAACGAGGATTGGACCGTACTGTCCAGGACCA
ATGTCGTGGGCTTCAAGCTGAAGATGAAGTCCGCTGAGGATTCTCCCTCTGAAGTGGTGAGGAACATCCTGGACCTTGTGTCCAACAAAGTGAGCGCCAT
CGTTGGCCCTTTCCGTAGCAGCCACGCCACCATCGCTTCCTACATCCTGACCGAGCTGCGAATCCCCATGATCACCCCCACCTCCACGGACCCTTTCCTGCG
TAATCCCCTTTGGAGCTCCTACCTGCTGCACATGCAGCCCCTTGACAATTACCAGTCCGAGGCTCTGCTGGATCTGGTCAAATACATGGATTGGAACTCCGT

GGCTCTGATCAGCTCTATGGACTCTTACGGTATCAACGGACTGACAACCTTCCAGAAGTACAGCAACGAACGTAAGATCAAGATTATGGCAAACGAGATGA
TCCCCCTGACCAAGAACATGAGCAGGCTGGACGTGAGCCAGCAGGTGAAGAACCTGAAGGATAGCGAAGCACGTATCTTCATCATGAACGTGCACTCTG
GATACGCCAAGCCTGTGCTGGATATCGCCCAGCAGATGGGCATTACCGGACCCGACTACGCCTGGGTCGTCACCGATGCTACCATCAAGGATGCCGATGG
ACTCATCAACAAGTACAACGAGTTCCCCGAGGCAATCCAGGGCATGGTGGGCTACATGTCCAACGTGTGCTGCTCCAAAAAGAAGGCCGAGTTCATGGAT
AAGTGGAGGACCGAGTCCGGCGGCGTGCAGGCCAAGGTGAGCTTCCCCGCCGATCAGATGAGATACTGGAGCGGCAACACCGAGAGCACCCCTACCGA
TAATGACGAAAGCGAGCTGAGGTCCCAGTGGAAGAGCCGTGCCAGCCTGACCTACGATTCCGTTTGGGTTATCGCCCAGGCCATCAACAAGCTGGCATAT
CTGGACAACATCCCCCTGAGCCCGCCCAACGTGGACTACTGGAGCTCTCCTGTGCGTTACTTCAACGAAGGCCAGACCATCCTGGATTACATCAGCAGGG
TCCAACTGGACGGCATCACCGGCATGATCGAGTTCGATGCCACCGGTGGCCCCGTCAGGAAGCTGTACGATCTGGTCAATTTCCGTGTCAACGATGTGGT
GAAGGTTGGCGAATGGTCCCCTAACATTGGCCTGAAGATGAACCAGACCGAACAAATCCTGTGGAACGGCGACAGCAAGCTGACCCCCTCCTCCTCCGT
CCAGTCCCTGGACGGCCGAACCCTGAGGCTGGGCATTACCCAGGAACCTCCTTTCATCTCCATCGACACCAATTGCACCGCCGAGGGACGTGGAAATGAG
TGTTACACCGGACTGTGCATCGAACTTGTGCAGAAGCTGGCAAAGCAGCTGCATTTCGACTTCGTGTTTATCGAGAGCGAGGACGGAAAGTTCGGATCA
CCCCTCGCCAACGGCAAGGGATGGAACGGAATCATCAAAATGCTGCTGGACGGCAAGCTGGACATCGGAACCGTCAACTTCGCTATGAATAAAAAGAGG
GAAGCAGCCATCGATTTCAGCATGCCCTTCATCAACACCGGCCTGGTAATGACCACCAAGATCGAGGAGGCTAAGAGCGACGCCTTCTTCTGGACCAAGC
CCTTCGATGTGGATCTGTGGTACGCCATTCTGGCTTTCGCTGTTCTGATGGTGCTGCTGATCTGGCTGTATGACCACATGTCCCCTTTCGGCTTCTACGGCCG
TAGGATGCACGCCGCCCTGCGTTGTGGCTGCGAAGAATGCCAGCAGTTCCGTTCCAACAAGGAGCGTAAGATCGATACCACGGATCACGATGAGTGCCTT
TTTGAGAGCCGTCTGGGTGACCCTAGCGAGAATTTCGAGGAGGTTATGTCCGTCGGCAACTCTCTGTGGATGATTGCCGCATGTCTGTTCAGCATCGGCCC
CGTGGAAGGTGTGCCACGTAACATGAGCGGAAGGGTGATACTGGCCATGTGGTGGTTCATGATCCTGATTGTGACCGCTATGTACACCGCCAATCTGGCT
GCCTTCCTGACCGTCACCAGGATGGACAGCGGCATCAACAGCATCAGCGATCTGATCGGTCAGAGCAAGGTGTCCTGGGGTACCGTGGCTGGCACCAAT
GCCGAAATCCTGCTGAAGTACGCCAATAGCGCTGAGCTGGAAGAGGTGTACAAGAAGCAGCAGCCCGTGAAGAGCGTGAAGGAAGGATTCGATCGTGC
CCGAAACGACGACTACATCTTCTTCTACGGTGGTGCTATCGTGACCTTCGAGGCTAACGAGCAACCATGCAACCTGCAGATCGTCGGCGAGCAGCTCTTCT
CTTTCGGCTACGCCTTCGGATTCCCCCCCACTCCCCTTACCTGGAGACCTTCAACGCCGCACTTCTTACCCTGAGCGAACAGAACGAACTGGATTCCCTGT
GGAAGAAGTGGAGCAAGGGTACCTGCTCTAGCAAGAAAAAGAGCAGCTCATCTAGCCTGAACCTGGCCAATATCTACGGACTGTGCTACACCTTCTTCGT
CGTGATCGCCGTGAGCGGCGTGATCCTGCTGGGAGAGATTATCTACGAGAGCACCATGGACATCAAGAGGTTTGAGACCAGCTTCCTTAGCGCCCTGAAA
TACAGGCTGATCTACATCTGGAAGCGTCCTAACTACCAGCAGTGTAAGTATCAGGACCTGGCCCCCGACGAGGGCTCCCAAACCAACACCATCAGGACCG
GCGACGGCAAGGATCTGATCCAGATGGTGGAGTACGACGGCTCGAAGAAGCCTACCGACCCCTACAAGATCAAAGACCCTGACGTGAAGCAGATGAGG
CAAGACATGGATCTGTCTTACCTGACCCCCGATCCCGACACCATTGTTCTGATAGGAGGAAATGGCTACCACAACAAGATGAACGGCTACGAGCTGGATCA
GAAGCCCATCCTGGACATCGATAAAGGCAGCCTGAAGTCTAACCAGTCAGCCAGGCAGTGGCCCAACCACTACCCCCCCGAATACCAACCGCTGCACCCA
GCTCGAGCCAATACCCTTCCCATGGACTACTCGCCCGCCAGCGAAAATGAGTATAATACCAAGAAGAGGAAGAACCGTGAGAACCTGAGGCGAAATAAC
ATCGGACAGAGCACCCCCCCCGTGAGCATGAACGGCGTCAAGGCCCCCCCTAACTTCGAGGAGCTGTTCGACAAGACCCTGCAGAGGATGACCTCCCAC
GACCAGTCCAGGGGCAAGGAATATGATAAGCCCAGAAGCGGCGACCAGAAGAGCATGGCTAGCACCCACAGCACCAAGATGTTTTCCAGCCCAGACATC
TCCGACACCGACGATCTGAGGAGCGAGAGCGATATGAGCCTGCCAACCTAAGGATCC

Supporting Information References

1 Armstrong, N., Sun, Y., Chen, G. Q. & Gouaux, E. Structure of a glutamate-receptor ligand-binding core in complex with kainate. *Nature* **395**, 913–917 (1998). https://doi.org/10.1038/27692

2 Sobolevsky, A. I., Rosconi, M. P. & Gouaux, E. X-ray structure, symmetry and mechanism of an AMPA-subtype glutamate receptor. *Nature* **462**, 745–756 (2009). https://doi.org/10.1038/nature08624

3 Moroz, L. L., Kocot, K. M., Citarella, M. R., Dosung, S., Norekian, T. P., Povolotskaya, I. S., Grigorenko, A. P., Dailey, C., Berezikov, E., Buckley, K. M., Ptitsyn, A., Reshetov, D., Mukherjee, K., Moroz, T. P., Bobkova, Y., Yu, F., Kapitonov, V. V., Jurka, J., Bobkov, Y. V., Swore, J. J., Girardo, D. O., Fodor, A., Gusev, F., Sanford, R., Bruders, R., Kittler, E., Mills, C. E., Rast, J. P., Derelle, R., Solovyev, V. V., Kondrashov, F. A., Swalla, B. J., Sweedler, J. V., Rogaev, E. I., Halanych, K. M. & Kohn, A. B. The ctenophore genome and the evolutionary origins of neural systems. *Nature* **510**, 109–114 (2014). https://doi.org/10.1038/nature13400

4 Srivastava, M., Begovic, E., Chapman, J., Putnam, N. H., Hellsten, U., Kawashima, T., Kuo, A., Mitros, T., Salamov, A., Carpenter, M. L., Signorovitch, A. Y., Moreno, M. A., Kamm, K., Grimwood, J., Schmutz, J., Shapiro, H., Grigoriev, I. V., Buss, L. W., Schierwater, B., Dellaporta, S. L. & Rokhsar, D. S. The Trichoplax genome and the nature of placozoans. *Nature* **454**, 955–960 (2008). https://doi.org/10.1038/nature07191

5 Najle, S. R., Grau-Bove, X., Elek, A., Navarrete, C., Cianferoni, D., Chiva, C., Canas-Armenteros, D., Mallabiabarrena, A., Kamm, K., Sabido, E., Gruber-Vodicka, H., Schierwater, B., Serrano, L. & Sebe-Pedros, A. Stepwise emergence of the neuronal gene expression program in early animal evolution. *Cell* **186**, 4676–4693 e4629 (2023). https://doi.org/10.1016/j.cell.2023.08.027

6 Warner, J. F., Guerlais, V., Amiel, A. R., Johnston, H., Nedoncelle, K. & Röttinger, E. NvERTx: a gene expression database to compare embryogenesis and regeneration in the sea anemone Nematostella vectensis. *Development* **145** (2018). https://doi.org/10.1242/dev.162867

7 Khalturin, K., Shinzato, C., Khalturina, M., Hamada, M., Fujie, M., Koyanagi, R., Kanda, M., Goto, H., Anton-Erxleben, F., Toyokawa, M., Toshino, S. & Satoh, N. Medusozoan genomes inform the evolution of the jellyfish body plan. *Nature Ecology & Evolution* **3**, 811–822 (2019). https://doi.org/10.1038/s41559-019-0853-y

8 Andrikou, C., Thiel, D., Ruiz-Santiesteban, J. A. & Hejnol, A. Active mode of excretion across digestive tissues predates the origin of excretory organs. *PLoS Biol* **17**, e3000408 (2019). https://doi.org/10.1371/journal.pbio.3000408

9 You, L., Chi, J., Huang, S., Yu, T., Huang, G., Feng, Y., Sang, X., Gao, X., Li, T. a., Yue, Z., Liu, A., Chen, S. & Xu, A. LanceletDB: an integrated genome database for lancelet, comparing domain types and combination in orthologues among lancelet and other species. *Database* **2019** (2019). https://doi.org/10.1093/database/baz056

10 Simakov, O., Kawashima, T., Marlétaz, F., Jenkins, J., Koyanagi, R., Mitros, T., Hisata, K., Bredeson, J., Shoguchi, E., Gyoja, F., Yue, J.-X., Chen, Y.-C., Freeman, R. M., Sasaki, A., Hikosaka-Katayama, T., Sato, A., Fujie, M., Baughman, K. W., Levine, J., Gonzalez, P., Cameron, C., Fritzenwanker, J. H., Pani, A. M., Goto, H., Kanda, M., Arakaki, N., Yamasaki, S., Qu, J., Cree, A., Ding, Y., Dinh, H. H., Dugan, S., Holder, M., Jhangiani, S. N., Kovar, C. L., Lee, S. L., Lewis, L. R., Morton, D., Nazareth, L. V., Okwuonu, G., Santibanez, J., Chen, R., Richards, S., Muzny, D. M., Gillis, A., Peshkin, L., Wu, M., Humphreys, T., Su, Y.-H., Putnam, N. H., Schmutz, J., Fujiyama, A., Yu, J.-K., Tagawa, K., Worley, K. C., Gibbs, R. A., Kirschner, M. W., Lowe, C. J., Satoh, N., Rokhsar, D. S. & Gerhart, J. Hemichordate genomes and deuterostome origins. *Nature* **527**, 459–465 (2015). https://doi.org/10.1038/nature16150

11 Luo, Y.-J., Kanda, M., Koyanagi, R., Hisata, K., Akiyama, T., Sakamoto, H., Sakamoto, T. & Satoh, N. Nemertean and phoronid genomes reveal lophotrochozoan evolution and the origin of

bilaterian heads. *Nature Ecology & Evolution* **2**, 141–151 (2018). https://doi.org/10.1038/s41559-017-0389-y

12 Kumar, S., Tumu, S. C., Helm, C. & Hausen, H. The development of early pioneer neurons in the annelid Malacoceros fuliginosus. *BMC Evolutionary Biology* **20**, 117 (2020). https://doi.org/10.1186/s12862-020-01680-x

13 Brandl, H., Moon, H., Vila-Farré, M., Liu, S.-Y., Henry, I. & Rink, J. C. PlanMine – a mineable resource of planarian biology and biodiversity. *Nucleic Acids Research* **44**, D764–D773 (2016). https://doi.org/10.1093/nar/gkv1148

14 Kozak, M. An analysis of 5'-noncoding sequences from 699 vertebrate messenger RNAs. *Nucleic Acids Research* **15**, 8125–8148 (1987). https://doi.org/10.1093/nar/15.20.8125

15 Seljeset, S., Sintsova, O., Wang, Y., Harb, H. Y. & Lynagh, T. Constitutive activity of ionotropic glutamate receptors via hydrophobic substitutions in the ligand-binding domain. *Structure* **32**, 966–978.e966 (2024). https://doi.org/https://doi.org/10.1016/j.str.2024.04.001

16 Singh, A., Zhorov, B. S., Yanez-Guerra, L. A., Aleotti, A., Koens, C. C., Yanartas, C. D., Song, Y., Miguez Cabello, F. J., Bowie, D. & Senatore, A. Evolution of iGluR ligand specificity, polyamine regulation, and ion selectivity inferred from a placozoan epsilon receptor. *Communications Biology* **8**, 994 (2025). https://doi.org/10.1038/s42003-025-08402-3

17 Rosano, G., Barzasi, A. & Lynagh, T. Loss of activation by GABA in vertebrate delta ionotropic glutamate receptors. *Proceedings of the National Academy of Sciences* **121**, e2313853121 (2024). https://doi.org/doi:10.1073/pnas.2313853121

18 Ramos-Vicente, D., Ji, J., Gratacos-Batlle, E., Gou, G., Reig-Viader, R., Luis, J., Burguera, D., Navas-Perez, E., Garcia-Fernandez, J., Fuentes-Prior, P., Escriva, H., Roher, N., Soto, D. & Bayes, A. Metazoan evolution of glutamate receptors reveals unreported phylogenetic groups and divergent lineage-specific events. *Elife* **7** (2018). https://doi.org/10.7554/eLife.35774
